# Widespread intra-dependencies in the removal of introns from human transcripts

**DOI:** 10.1093/nar/gkx661

**Published:** 2017-07-29

**Authors:** Seong Won Kim, Allison J. Taggart, Claire Heintzelman, Kamil J. Cygan, Caitlin G. Hull, Jing Wang, Barsha Shrestha, William G. Fairbrother

**Affiliations:** 1 Department of Molecular and Cellular Biology, Brown University, Providence, RI 02903, USA; 2 Center for Computational Molecular Biology, Brown University, Providence, RI 02903, USA

## Abstract

Research into the problem of splice site selection has followed a reductionist approach focused on how individual splice sites are recognized. Early applications of information theory uncovered an inconsistency. Human splice signals do not contain enough information to explain the observed fidelity of splicing. Here, we conclude that introns do not necessarily contain ‘missing’ information but rather may require definition from neighboring processing events. For example, there are known cases where an intronic mutation disrupts the splicing of not only the local intron but also adjacent introns. We present a genome-wide measurement of the order of splicing within human transcripts. The observed order of splicing cannot be explained by a simple kinetic model. Simulations reveal a bias toward a particular, transcript-specific order of intron removal in human genes. We validate an extreme class of intron that can only splice in a multi-intron context. Special categories of splicing such as exon circularization, first and last intron processing, alternative 5 and 3′ss usage and exon skipping are marked by distinct patterns of ordered intron removal. Excessive intronic length and silencer density tend to delay splicing. Shorter introns that contain enhancers splice early.

## INTRODUCTION

The information encoded in eukaryotic genes is stored as sequence fragments throughout a transcript. These fragments are spliced out of pre-mRNA and concatenated into messenger RNA by a process called splicing that is catalyzed by the spliceosome. On average, a eukaryotic gene is interrupted by about 10 introns that comprise 90% of the total length of the gene. There is a wide variance in intron/exon architecture; there exist hundreds of small genes with no introns but also many large complex genes like Titin, which consists of 364 exons that vary widely in size, the largest being 17 106 nt (1).

Out of necessity, much of the initial biochemical characterization of splicing utilized a few model substrates (e.g. Adenovirus, beta-globin). An *in vitro* splicing system that consisted of single intron substrates was developed to define the factors and sequence requirements of spliceosome assembly. The core spliceosome was shown to consist of riboprotein particles called snRNPs that assemble in a stepwise fashion over the 5′ss, the branchpoint and eventually the 3′ss to catalyze splicing across the intron. The spliceosome was proposed to commit to a splicing event early in the assembly process. However, splicing has subsequently been demonstrated to be a reversible process so this commitment cannot be absolute (2). The precise mechanism of how splice sites, exons and introns are recognized is thought to vary depending upon substrate features (i.e. intron length, exon length and possibly the composition of exonic and intronic enhancers). During spliceosome assembly, the 5′ss and 3′ss are recognized in a coordinated fashion across the intron. Mutational and some biochemical evidence suggest that this coordinate recognition can also occur across the exon (3). Exon skipping, the most common consequence of a mutation in either the 5′ or 3′ss, is regarded as evidence of this dependence between upstream 3′ss and the downstream 5′ss recognition (4). This mode of recognition, termed exon definition, is thought to favor a short exon positioned between relatively long introns (5). In addition, there is a variety of other mechanisms hypothesized to influence splice site selection such as secondary structure and kinetic phenomena associated with co-transcriptional splicing (6–9).

While exon definition may describe a mechanism for recognition, it does not effectively explain the fidelity of splicing. Examining the information content of the individual splice signals in small introns suggests that splice signals supply only 50% of information required for a recognition event (10). The additional restrictions of the exon definition model, the requirement of a 5′ss in a 50–250 nt downstream window adds very little information to the 3′ss. Additional cis-elements such as exonic and intronic enhancers and silencers (i.e. ESE, ESS, ISE and ISS’s) can also help specify splice sites. While these elements are typically located near the splice site they modulate, neighboring introns or splicing events can also affect splicing. Experiments with minigenes containing a chimeric optimized splicing construct comprised of duplicated fragments from a beta-globin intron demonstrates that the act of splicing can increase splicing efficiency of a neighboring intron. This enhancement was initially hypothesized to involve the exon junction complex (EJC)—a tightly bound set of proteins deposited on the newly spliced exon by the spliceosome that has been implicated in the splicing, export and translational surveillance of mRNA (11). However, subsequent experiments implicated retained components of the exon definition complex as the source of the enhancing activity (12). Other studies have inferred higher-order effects between introns from measuring the order in which introns are removed from a transcript (13).

Early studies with multi-intron splicing substrates offered evidence that introns were removed in a particular order through a defined pathway that did not always match the order in which they were transcribed (14). A transcript could appear to splice in a particular order because (i) the removal of one intron is dependent on the removal of another or (ii) because the individual introns are spliced at dramatically different rates. Early suggestions of dependencies between splicing events came from the analysis of complex splicing phenotypes that arose from disease causing splicing mutations. Over the years there have been numerous reports of single point mutations in disease genes (e.g. COL4A5, VPS 33B, BTK, COL1A1 and NF1) that disrupt a splicing element and result in processing defects affecting multiple introns (15–19). In the case of NF1, a mutation in exon 37 led to the skipping of exon 36 and exon 37, however the entire genomic region stretching from exon 31 to 38 was necessary to recapitulate this result (19).

Here, a novel genome-wide method is utilized to extract pairwise order of splicing measurements for adjacent introns from RNA-seq data. The order of splicing inferred from sequencing reads is well correlated with the order of splicing determined through traditional methods. In considering all adjacent intron pairs in the genome, certain introns have a strong bias toward splicing before their neighbors (i.e. ‘always-first’ introns). Examining all of the pairwise splicing orders revealed that the number of ‘always-first’ outcomes was in significant excess to that predicted by a purely kinetic model of transcription and splicing. We report an extreme category of nuclear introns that are only able to splice after neighboring splicing events have occurred. These dependencies appear to be utilized by the cell to delay intron removal and enable alternative splicing pathways.

## MATERIALS AND METHODS

### Order of intron removal

In order to determine the order of intron removal in COL4A5, VPS33B and BTK genes, intronic and exonic primers were designed using Primer3. (See Supplementary Table S2 for all primer sequences and figures for splicing patterns observed in mutant). Primers were purchased from IDT. Total RNA from HEK293 cells was used after treatment with DNAse (New England Biolabs). Three cDNA from wild-type samples were diluted to equal concentration of 350 ng per microliter. One microliter of each cDNA samples was used for the subsequent polymerase chain reaction (PCR) reaction using the designed primers with GoTaq Green Master Mix (Promega) following the manufacturer’s instruction.

### Global analysis of order of intron removal

Paired end reads from ENCODE (GSE26284) (20–23) that were 200 nt, non-polyAdenylated and from either the total cell or nucleus to enrich for partially spliced reads were aligned to the human genome using TopHat. Using a combination of custom Perl scripts, samtools and bedtools, the pairs were filtered for reads that contain at least one spliced intron (Supplementary Figure S2, http://fairbrother.biomed.brown.edu/data/Order/). These pairs were then intersected with a bed file of all introns from UCSC hg19 database. Pairs that contained both evidence of a spliced intron and an intronic sequence were then counted as an intermediate read (i.e. a partially spliced transcript). Intron pairs associated with 10 or more intermediate reads per pair were retained for further analysis. Those with <10 intermediate reads were discarded.

### Simulation

Intron pairs were selected where both introns were unique (both 5′ splice sites and both 3′ splice sites unique among all transcripts). For these pairs that also had 10 or more partially spliced reads to support an upstream/downstream first percentage, splicing rates were randomly generated according to a normal distribution with a mean of 0.169 introns/min and a standard deviation of 0.048, as observed in the literature for splicing rates (24). These rates, along with the length of the internal exon and the downstream intron, were used with an average transcription rate of 3.87 kb/min to calculate a fraction expected for upstream intron splicing before downstream based on a purely kinetic model. The same number of reads as had been observed for the gene were used to simulate splicing for the pair with this expected distribution, which were then used to determine the overall distribution of percent splicing either upstream or downstream first. Simulations were also rerun varying parameters (Supplementary Methods).

### Splicing constructs

Two different splicing constructs were used in this paper. For cloning of COL4A5, VPS33B and BTK genes, all primers targeting exon sequences were designed by Primer 3 as described to include more than half of flanking exons. BamH1 and Nhe1 digestion sites and 4 nt overhang to enhance digestion were added at the end of the primers. From human genomic DNA (GenScript), the insert (i.e. introns and flanking exons) were amplified using Herculase 2 (Thermo Fisher) according to the manufacturer’s instruction. For the multiple intron clones, when the insert sequence was longer than 1000 bp, two introns and flanking exons were amplified separately and they were ligated using overlap extension PCR. For the clones containing more than two exons and single intron, exons were amplified from the total HEK cDNA and ligated with the exon using overlap extension PCR. All PCR reactions followed the manufacturer’s instruction. Then all DNA fragments were purified using PCR purification kit (Qiagen). The fragments were subsequently digested with BamH1 High Fidelity enzyme and Nhe1 (both New England BioLab) for 4 h. The digested pieces were cloned into the digested and gel purified (Qiagen) vector pzW4 using T4 DNA ligase (New England BioLab). After 30 min of ligation at room temperature, the vectors were transformed to TOP10 *Escherichia coli* cells (Thermo Fisher). Cells were plated on LB+Ampicilin plates. Colonies were randomly selected and they were inoculated in LB+Ampicilin medium for at least 16 h. Plasmids were then extracted by Miniprep kit (Qiagen) following the manufacturer's instruction. The insertion of the target sequences was verified by Sanger sequencing.

For cloning of later and earlier splicing introns 10 introns <150 nt were randomly chosen. Five introns shorter than 220 nt that splice before their neighbors more than 90% of the time were also randomly selected as positive controls. Primers were designed to amplify 10 nt of flanking exons and intron. Overlapping sequences with Ad81 exon and ACTN exon were added on all forward and reverse primers, respectively, to add the amplified intron to the minigene construct using overlap extension PCR. Using Herculase 2, the targets were amplified from Human Genomic DNA. After amplification and purification with PCR purification kit, second set of PCR was performed in order to increase the length of the overlap regions to increase efficiency of the ligation. CMV promoter, Ad81 exon, ACTN exon and polyA signal were added using overlap extension PCR. The final piece was cloned into PCR2.1 vector using TOPO TA cloning kit for subcloning (Thermo Fisher). Clones were transformed to TOP10 *E. coli* cells and plated on LB+Ampicillin+X-gal plates. Plasmids were extracted using Zippy Plasmid Miniprep Kit (Zymo Research). The constructs were verified by sequencing. A total of 400 ng TOPO vector was transfected into HEK293 cells and harvested after 48 h.

### Cell culture and transfection

HEK293 cells were maintained in Dulbecco's modified Eagle's medium (Life Technologies) supplemented with 10% fetal bovine serum (HyClone) and 1% penicillin/streptomycin. They were cultured in 37°C in 5% CO_2_ and humid condition. Cells were seeded in 12-well plate a day before transfection. When cells reached about 70% confluency, 1 μg of the plasmids were transfected using Lipofectamine 3000 (Thermo Fisher). Three individual transfections were made per construct in order to make biological replicates. After 48 h from transfection, total RNA was extracted using Trizol (Thermo Fisher). DNA was removed by DNase 1 Amp Grade (Thermo Fisher). The first strand was synthesized using random 9mer. Reverse transcription was done using SuperScript IV or SuperScript III (Thermo Fisher) following the manufacturer’s instruction. RT-PCR was performed using GoTaq Master Mix (Promega). Primer that targets 5′ untranslated region of the plasmid was used for the RT-PCR to specifically amplify the transfected gene and not endogenous sequences.

### Skipping

Using ‘knowngene’ annotation from UCSC hg19, all introns where both splice sites exactly matched the 3’ splice site from one intron and the 5′ splice site from the next consecutive intron were included in the analysis. For single and multiple exon skipping events, the transcript model that when fully spliced yielded the inclusion isoform was used for ‘before’, ‘after’ and ‘internal’ splicing events.

### Alt SS

Using all transcripts from UCSC hg19, alternate 5′ splice sites were determined when multiple introns had an identical 3′ splice site and different 5′ splice sites. For these cases, the 5′ splice site that gave the shortest intron was chosen to be used in the pair analysis. Alternate 3′ splice sites were determined from introns sharing a 5′ splice site with different 3′ splice sites, and again the shortest intron was used.

### First/middle/last

Intron number in transcripts was determined according to data from UCSC hg19 knowngene annotation. For any pair that contained the first and second introns in a gene, this was counted as a ‘first’ pair. Pairs containing the second-to-last and last introns in a gene were called ‘last’ pairs. ‘Middle’ pairs were those that did not fit into either category above. Only genes with more than two introns were used for these categories.

### Circles

A list of known circles generated from exonic back splicing junctions from publicly available RNAseq data was used to find circles that contained at least two exons in the dataset. To determine splicing order for before the circles, the beginning coordinate of the circle was matched to the internal exon of an upstream/downstream pair for that exon, matching it to the last (downstream) coordinate of the upstream intron. For after the circles, the end coordinate of the circle was matched to the first (upstream) coordinate of a downstream intron in a pair. Histograms were then generated for both the ‘before’ pairs and the ‘after’ pairs.

### Circle prediction and validation

Total RNA was reverse transcribed using the SuperScript III First-Strand Synthesis System (Life Technologies) with random 9-mer primers (Integrated DNA Technologies (IDT)) according to the manufacturer’s instructions. A nested PCR strategy was implemented using 30 cycle PCR amplification via outer primers (see Supplementary Table S2 for primer sequences) on the Platinum Taq DNA Polymerase System (Life Technologies) according to manufacturer's instruction. Thirty-five additional cycles of PCR were performed on product from the initial PCR reaction using nested primers. Poly-A cDNA was generated with the SuperScript III First-Strand Synthesis System using ReadyMade Oligo dT 16 mer from IDT. RNAse A digestions were performed according to manufacturer(Thermo Fisher) instruction: 37°C incubation for 30 min.

### Length analysis

Length of annotated introns was determined, and the difference in length between the introns in each pair was determined by subtracting the downstream intron’s length from the upstream intron’s length, so that a negative number represented a longer downstream intron and a positive number represented a longer upstream intron. Intron pairs were then binned according to their length difference. For each bin, the fraction of pairs where the downstream intron spliced first at least 95% of the time was determined and then plotted. Splicing percentage histograms were also created for each bin.

### Motif insertion

To test the effect of RBP motifs on splicing, genomic fragment spanning 2 exons and 1 intron from COL4A5 was cloned into pzW4 vector between the BamH1 and Nhe1 sites. Subsequently, the selected RBP motifs were inserted at −45 nt position by PCR. The inserted motifs were CTTTTCT and TTTGGTTT. Two copies of the motif ATCAACG (the only motif enriched in always-first splicing introns) was inserted into position −50 of the intron of the single intron minigenes of COL4A5 (Intron 50) and VPS33B (intron 9). The two copies of motif were separated by 5 nt of endogenous sequence. Upstream exon and motif and downstream exon and motif were amplified separately using Genomic PCR (Herculase 2) and overlap extension PCR was used to generate inserts. BamH1 and Nhe1 insert fragments were cloned to pzW4. After transformation into TOP10 cells and inoculation, the plasmids were extracted (Zippy Plasmid Miniprep Kit -Zymo Research) amplified and sequence. A total of 1 μg of the purified plasmids were transfected to the HEK293 cells and RNA was extracted after 48 or 17 h with Trizol and cDNA was synthesized using SuperScript IV. RT-PCR samples were sequenced using TOPO TA cloning kit (Thermo Fisher).

## RESULTS

### Mutations that cause multi-exon skipping occur in introns that splice last

**Figure 1.**
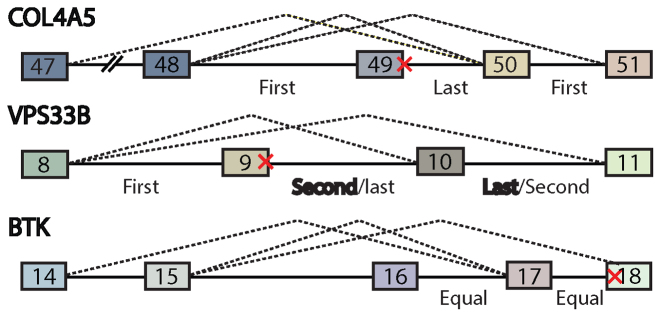
Introns proximal to the reported mutations splice last. Three cases where mutation caused multiple exon skipping were found on HGMD. Red x denotes location of reported mutation. Dashed line shows the reported splicing pathways. All cases led to disease. cDNA was synthesized from random 9mer selected HEK293 total RNA. In all cases, intron that is proximal to the mutation was the last one to be removed. In case of VPS33B, the major species removed intron 10 first. PCR amplification of intron 14 and 15 of BTK gene failed.

**Figure 2.**
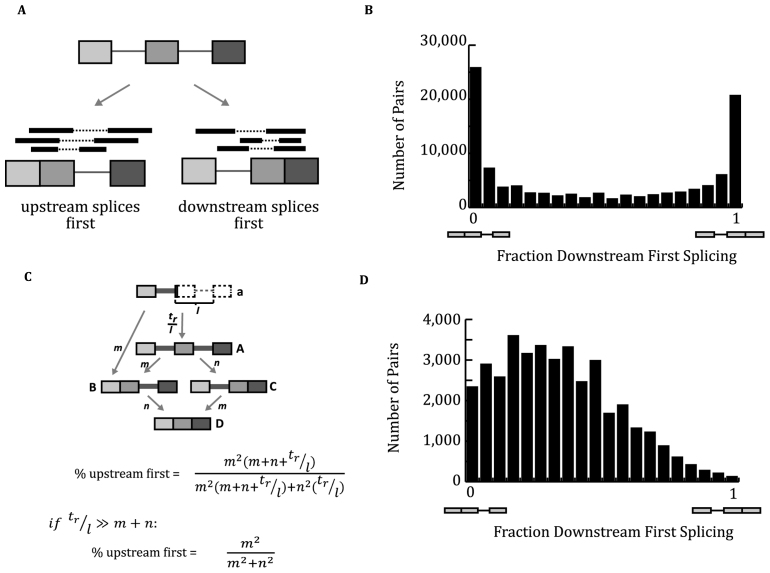
Genome-wide analysis of order of splicing showed excess of always-first events. (**A**) Pipeline to determine the order of intron removal. ENCODE non-PolyA RNA reads were analyzed to count reads that mapped to partially spliced species. Partially spliced species were compared to generate the pairwise fraction downstream intron goes first. (**B**) Determined order of intron removal in genome-wide scale. All pairwise events were used to compute the frequency of downstream intron splicing first. Events were sorted and binned (x-axis). Pairs that have read counts >10 were included and used for further analysis. (**C**) Kinetic model of splicing using steady-state assumptions. (**D**) Simulation of intron removal using purely kinetic model. Using published rates of Pol II elongation and *in vivo* splicing rates, distribution of splicing order was simulated. Average splicing rate of 0.169 introns/minute, standard deviation of 0.048 and transcription rate of 3.87 kb/min were used (24). Contrary to the observed data, the simulation results did not show excess of always-first events.

Base substitutions that result in multi-exon skipping phenotypes are indicative of inter-dependencies between intron processing events. In order to study such events, three disease alleles in COL4A5, VPS 33B and BTK were selected for further examination. The organizing assumption was an intron splicing event that was dependent upon another intron would always splice after that intron. To determine the order of splicing, RT-PCR primers were designed to amplify all potential intermediates in the manner of Kessler and Chasin for the transcript region that contained the mutated site (Figure 1) (14). The wild-type version of all three genes was examined and used to determine the order of splicing. RT-PCR results are shown in Supplementary Figure S1. In all three cases, the intron that contained the disease allele was the last or one of the last to be removed from this region of the transcript in RNA extract from 293 cells. To determine if this order was found in other cell lines and tissues, the entire ENCODE total RNA-seq dataset was downloaded and analyzed for reads that detected partial intermediates. In this study, a partial intermediate is defined by a paired end read with one pair mapping to an intron and the other pair spanning a spliced, exon/exon junction (Figure 2A and Supplementary Figure S2). This data provided pairwise order of splicing data (i.e. the number of counts supporting the upstream intron splicing before versus after the downstream intron). The order of splicing results drawn from 14 different ENCODE cell lines confirmed the results in HEK293 for COL4A5, VPS 33B and BTK. In addition to these three loci, the read data mapped to ∼100 000 additional intron pairs. To verify that inferences drawn from public RNA-seq data were consistent with the conclusions drawn from RT-PCR measurements of splicing intermediates, we compared order of splicing inferred from ENCODE data to previous literature and found 78% agreement with prior reports (Supplementary Table S1).

### A genome-wide comparison of the splicing order of introns returns an excess of ‘always-first’ outcomes

Comparing the pairwise splicing reads enabled the discovery of a patterns in the processing of multi-intron transcript by the splicing machinery. Intron pairs were binned according to the fraction of reads that support the splicing of the downstream intron splicing first. In agreement with prior observations that splicing is co-transcriptional the distribution slightly favors the processing of the upstream intron first (Figure 2B). There appeared to be an excess of data in the first and last bin, which reflect cases where one intron precedes another by a large margin (>20:1). These bins were called ‘always-first’ outcomes and could reflect dependencies between introns or they could arise due to widely different splicing rates across introns. Using assumptions of steady state and post-transcriptional splicing, the ratio of one splicing event occurring before another is a function of the square of the ratio of the individual rates (Figure 2C, derivation in Supplementary Figure S3). Considering co-transcriptional splicing, the ratio of one splicing event occurring before another is also dependent on the rate of transcription across the downstream intron (Figure 2C). As splicing and transcription rates (and their standard deviations across loci) have been recorded *in vivo* by several different groups, an *in silico* spliceosome was programmed to simulated the co-transcriptional splicing of two consecutive introns in a transcript ((24), and references within). This *in silico* spliceosome, parameterized by published kinetic data, was used to generate the distribution of order of splicing data predicted by an assumption of independence between individual intron splicing rates (often referred to as a kinetic model) (Figure 2D and Supplementary Figure S4). These simulated distributions were compared to the pairwise orders observed from the RNA-seq data. The observed data contained a significant excess (*P*-value < 0.001, chi-square test) of ‘always-first’ introns whose removal appears to be a prerequisite of neighboring splicing events. Furthermore, many introns always splice last, with their splicing being observed after both neighbor introns. We named these introns ‘local slowpokes’. The ENCODE data contains 26 000 of introns that always (95%) spliced after their upstream counterpart, 20 000 introns that always spliced after their downstream counterpart and 18 000 introns spliced after both their upstream and downstream counterpart (local slowpokes). Taken together, these results suggest that intron removal in a transcript is not independent and the removal of local slowpokes may be dependent on neighboring splicing events.

### A category of nuclear introns can only splice conditional on neighboring splicing events

**Figure 3.**
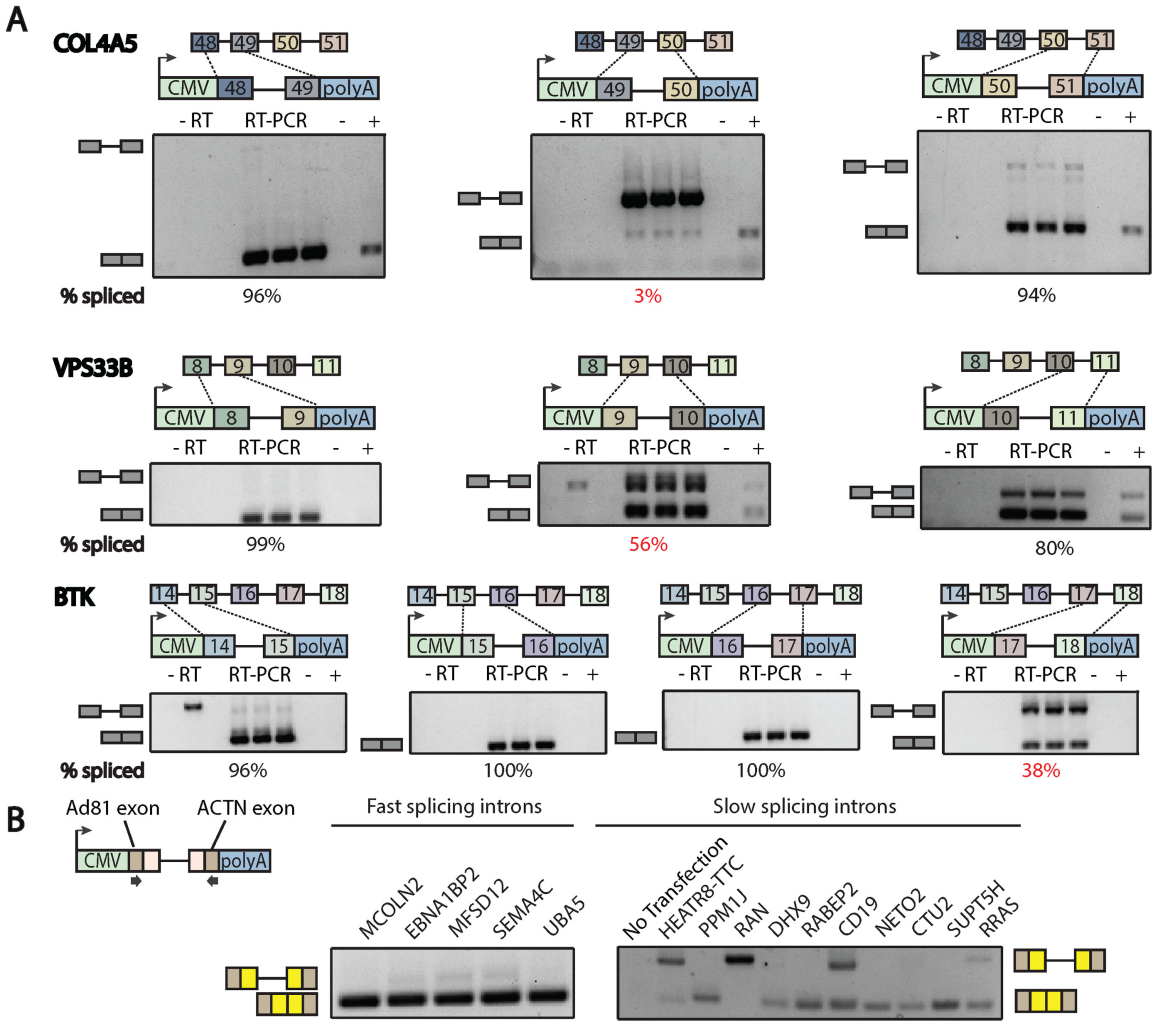
Always-last splicing introns are likely show lower splicing efficiency in a single intron mingene construct. (**A**) Introns that were affected by the mutations and their flanking exons were individually cloned into the Plasmid pzW4 using BamH1 and Nhe1 cloning site. Plasmid-specific forward primer was used to amplify only the construct, not endogenous sequences. Negative control was total 293 cDNA without transfection amplified using specific primers. Positive control was total cDNA amplified using endogenous sequence targeting primers. Introns close to the reported mutations showed lowest splicing efficiency among neighbors (Intron 49 of COL4A5 gene/Intron 9 of VPS33B gene and Intron 17 of BTK gene). (**B**) Always-last splicing introns showed lower splicing efficiency in minigene construct. Five introns that splice first more than 90% of the time and 10 introns that always splice last among neighbors in the genome-wide screen were selected and cloned into minigene constructs. Always-first splicing introns showed high splicing efficiency whereas always-last splicing introns showed varying splicing efficiency.

The simulation suggested the existence of a class of functional introns whose removal required the splicing of adjacent introns. To test whether these introns were competent for splicing but inactive outside a multi-intron context we selected local slowpoke introns and created a panel of splicing reporter constructs to study their processing. We initially focused on introns that spliced after their neighboring introns as determined by both RNA-seq and the RT-PCR determinations (e.g. introns 49, 9, 17 in COL4A5, VPS 33B and BTK, Figure 1). The three candidate introns were tested in isolation as a single intron reporter construct, transfected into human HEK293 cells and scored for inclusion by RT_PCR analysis of extracted total RNA. At all three loci, the introns that were processed after neighboring splicing events did not splice to completion when tested alone (i.e. splicing efficiency was between 0 and 56%) (Figure 3A, red font). Although the minigene has been used in a variety of previous studies (25–29), it is possible that a negative context or some other feature of the minigene system inhibits the splicing of these inserted introns. To see if all introns were processed with reduced efficiency when tested alone in this context, the adjacent introns that spliced first were also assayed as single intron constructs. All but one intron was processed efficiently (≥95%). The exception to this rule was a neighboring intron that also spliced late. Overall, the comparison of the individual intron splicing efficiencies appeared to match the order of splicing observed *in vivo*, however at the endogenous loci, the introns processed last are eventually spliced to completion. Surprisingly, three introns that appear to be processed constitutively in a multi-intron transcript do not splice to completion when tested alone. To determine if this is a general phenomena in the ENCODE dataset, 10 randomly selected introns that match this profile of mostly splicing last were compared to five control introns that splice first (‘Materials and Methods’ section). The early splicing introns spliced well in the mini gene construct whereas the selected always-last splicing introns demonstrated lower splicing efficiency (Figure 3B and Supplementary Figure S5). This result suggests that always-last spliced introns are not merely a kinetic phenomena but may reflect a dependency of later splicing introns on neighboring splicing events.

**Figure 4.**
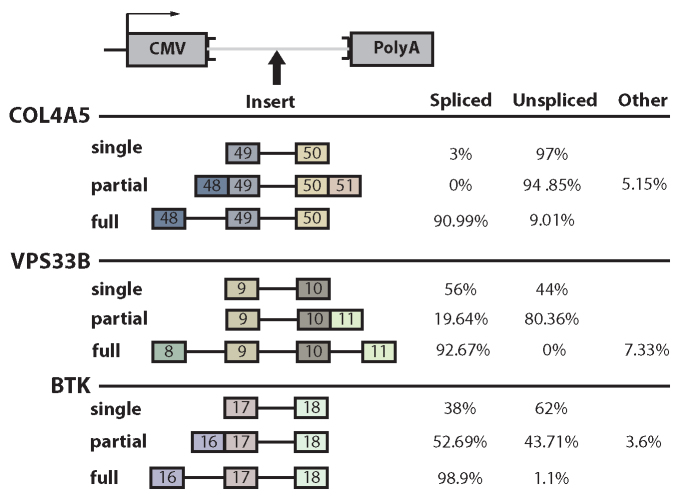
The act of splicing enhances the subsequent splicing of neighboring introns. Various portions of neighbor sequences were included in the minigene construct. Including neighbor introns increased splicing efficiency whereas including neighbor exon sequences and creating synthetic splicing intermediates did not increase splicing efficiency. Multiple intron constructs often led to exon skipping, and the abundance of the skipped product is shown in the ‘other’ column.

To ensure that it was the lack of adjacent introns that was responsible for the inhibited splicing, three constructs were expanded to include the neighboring introns and retested. At all three loci, the inclusion of neighboring introns increased the splicing of the affected intron (>90% percent spliced, Figure 4—marked ‘full’, Supplementary Figure S6). This result suggests that splicing enhancement is obtained by either the process of splicing or perhaps the sequence of the intermediate. The intermediate contains novel sequence (i.e. an enlarged exon created by a prior splicing event) which could conceivably bring an exonic splicing enhancer in close enough proximity to enhance the late-splicing intron. To test whether this splicing intermediate with concatenated exons alters splicing, chimeric cDNA/genomic constructs were made to mimic the intermediates (Figure 4—marked ‘partial’, Supplementary Figure S6). Adding the expanded exon sequence did not result in any consistent increase or decrease in splicing efficiency across the three substrates. However, the presence of an additional intron results in a complete or near complete rescue of splicing in all three cases. These data suggest that the process of splicing, but not the sequences of intermediates, greatly enhances these dependent introns. It is unclear how many human introns are dependent on neighboring splicing events. A dependent intron would have to splice after at least one of its neighbors. Extrapolating the 40% validation rate of Figure 3B on the 18 000 introns that always splice last predicts ∼7200 such introns in the human genome. To determine if these dependencies within pre-mRNA processing pathways are performing a biological function, alternatively spliced substrates were subject to further study.

### Distinct order-of-splicing mechanisms characterize different cellular alternative splicing programs

**Figure 5.**
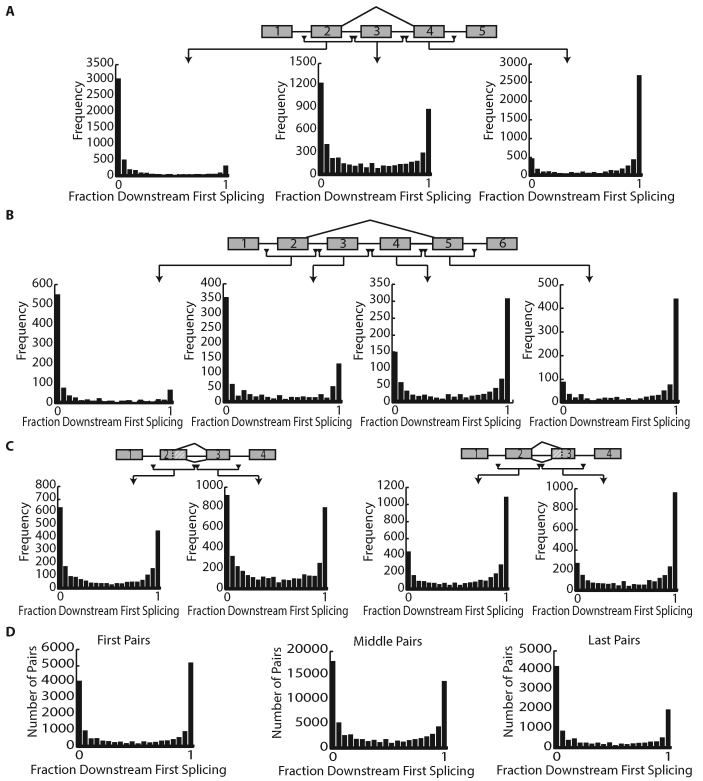
Alternative splicing loci utilize distinct order of splicing patterns. (**A**) Fraction of downstream first splicing surrounding single exon skipping events (from UCSC hg19 knowngenes). The three plots denote the splicing order of the introns surrounding the exon upstream of the conditionally skipped exon (left), the splicing order of the two introns surrounding the conditionally skipped exon (middle) and the splicing order of the introns surrounding the exon downstream of the conditionally skipped exon (right). (**B**) Fraction of downstream first splicing surrounding multi-exon skipping events (UCSC hg19 knowngenes with two skipped exons). (**C**) Fraction of downstream first splicing surrounding alternative 5’ss (left) and alternative 3’ss (right). (**D**) Fraction of downstream first splicing surrounding first introns, middle introns and last introns in a transcript (UCSC hg19 knowngene, see ‘Materials and Methods' section).

Splicing occurs co-transcriptionally at a rate that would predict that many introns splice before the downstream neighbor has been completely synthesized. For alternatively spliced substrates, this observation has been interpreted as an indication that a special mechanism is required to delay splice site selection until both splice sites were available. To determine if specific orders of intron removal are utilized in alternative splicing, we fractionated the data into different categories based on the type of alternative splicing observed. While there was little difference in splicing order between the introns upstream and downstream of a skipped exon, both of these flanking introns exhibited a greater tendency to be spliced after their peripheral neighbors (Figure 5A). In other words, the processing of the introns internal to the skipping event was delayed even when the inclusion pathway was followed (note: the order of splicing can only be determined for transcripts following an inclusion fate). This trend of late splicing in introns that are internal to the skipping event is continued in transcripts that underwent multiple skipping (Figure 5B). Regulated exon skipping requires a choice between splice sites in adjacent introns. However, most introns are spliced prior to the synthesis of the downstream intron. These data suggest that delay mechanisms exist to ensure the availability of competing alternate splice choices. A similar requirement for a delay in splicing exists for exon circularization events where a downstream 5′ss splice site pairs with an upstream 3′ss. In order to discover how a delay mechanisms could govern other types of splicing, alternate 5′ss usage was examined (Figure 5C, left). While introns flanking alternate 5′ss resembled background splicing patterns, alternate 3′ss were associated with a significant deviation from the expected linear processing events. Alternate 3′ss usage was associated with significant retrograde splicing (i.e. downstream introns processed before upstream introns) in the three introns that encompass the alternatively processed region (Figure 5C, right). In addition to alternately spliced introns, first and terminal introns constitute a distinct class of intron that differs from internal introns in the size of the flanking exons and the lower reliance on exon definition for processing. Analyzing splicing orders suggested a higher tendency for first and terminal exons to splice after their neighbor (Figure 5D). Taken together, intron to intron processing dependencies and particular order of splicing appears to be utilized by the cell to orchestrate alternative splicing events.

### Intron length and poly U signals exhibit strong influence on splicing order

**Figure 6.**
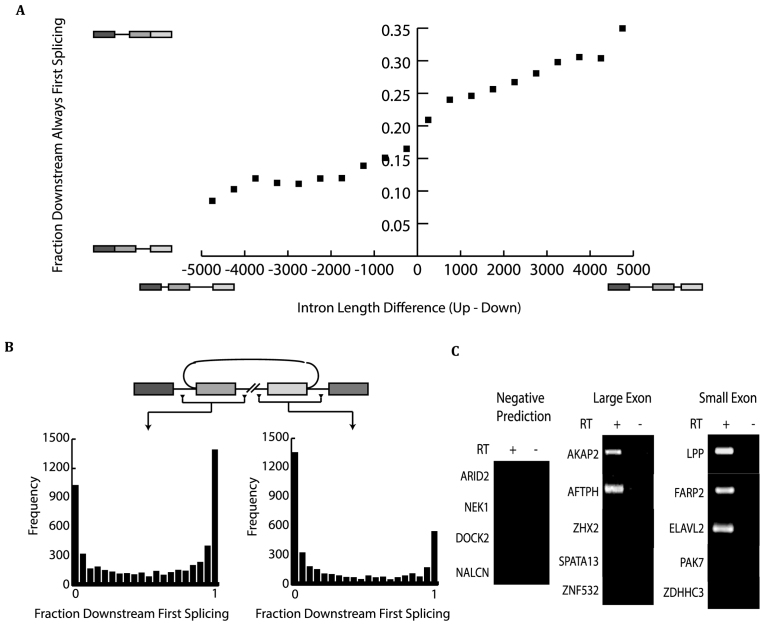
Circular splicing exons are flanked by long, always-last splicing introns and contain short, always-first splicing internal introns. (**A**) Fraction of downstream always-first splicing as a function of intron pair length differences. Intron pair length difference is defined as *length of upstream intron—length of downstream intron*. Pairs are binned by intron length difference and the percent of pairs with at least 95% downstream intron splicing first were plotted. (**B**) Fraction of downstream first splicing around circular splicing events discovered in RNAseq data. (**C**) PCR of predicted circles encapsulating one exon. hg19 genome assembly was scanned for coordinates predicted to be circles based on their ability to fit the following criteria: flanking introns of at least 10 kB and containing CCDS start sites in both the hg19 and mm9 assemblies. Predicted circles were tested using a nested PCR strategy on random 9-mer selected RNA from HEK293 total RNA. Large exon sizes range from between 1900 and 2800 bp and small exon sizes range from between 200 and 340 bp. Negative predictions included five coordinates not predicted to be circles. The prediction was based on their lack of a consensus start site, short (<250 bp) flanking introns and long average interior intron sizes were tested for circularization potential using nested PCR.

To better understand mechanisms that enforced order of splicing, pairwise comparisons of sequence features in upstream and downstream introns were performed. Splice site strength was not a discriminating feature in that similar strengths were observed in introns that splice first or last (data not shown). Intron length is associated with order of intron removal. Longer introns tend to be removed after shorter introns (Figure 6A and Supplementary Figure S7). On alternate splicing pathway, circular splicing (Figure 6B) was characterized by always-last splicing introns flanking the circle. If longer introns delay splicing transcripts that contain two long introns would be expected to circularize. To test this idea, we selected 10 transcripts with that contain two introns >15 kb and no prior evidence of circularization. Remarkably, 60% of these cases generated circular exons whereas control amplifications yielded only linear transcripts (Figure 6C).

In addition to length, it was possible that splicing factor binding could influence the order of splicing. Analysis of the density of 175 RNA binding protein binding motifs in pairwise data, revealed 17 motifs corresponding to nine RBP that significantly influenced splicing. The binding of a single factor, SRSF3, was associated with splicing last (Supplementary Figure S8A). The remaining 16 motifs were enriched in introns that spliced first (Supplementary Figure S9 and Table S3). As the motifs that promoted early splicing were highly similar U-rich sequences, two representative motifs were selected for further study. To test whether the U-rich sequences could influence splicing, two copies of each sequencing motif was inserted into an intron that was previously demonstrated to exhibit minimal levels of splicing when cloned into a single intron construct (Figure 3 and Supplementary Figure S8B). While low levels of unspliced transcript was detected in the uninserted construct, insertion TTGGTTT showed increase in splicing (Supplementary Figure S8B). Finally, the SRSF3 binding motif was tested for a negative effect on splicing as this motif is enriched in introns that splice last. Inserting this sequence into a wild-type intron had no effect. However testing the motif in an intron that was only partially recognized decreased intron removal (Supplementary Figure S8B).

## DISCUSSION

This study mapped the order of splicing with the goal of systematically measuring the synergies between the multiple splicing events that occur in human transcripts. Initially, RT-PCR was used to map the order of intron removal in three wild-type substrates that prior reports suggest may contain dependent introns (16,17,30). Subsequently, a novel method of inferring splicing order from paired end RNA-seq studies performed on total RNA confirmed and extended the initial finding that introns containing mutations that disrupt multiple splicing events often splice last.

While the order of splicing can be influenced by the rate of transcription and splicing, splicing kinetics and commitment order are distinct phenomena. For example, the splicing of a dependent downstream intron that requires an upstream splicing event may splice at a faster rate than the upstream intron because of the lag in synthesis of the downstream intron. The approach described in this work utilized a simulation of co-transcriptional splicing that was parameterized by published transcription and splicing rates and variances. Comparing the simulation to the observed data illustrated several incompatibilities between the experimental data and a purely kinetic model. It allowed several conclusions to be drawn about the observed data. There is an excess of ‘always-first’ events (Figure 2B, upper panel first and last bin). This result is robust to varying the parameters of the simulation (Supplementary Figure S4). Within the always-first category, there is less bias toward upstream splicing first than in a purely independent (i.e. kinetic) model. Intron splicing rates were not dependent on intron length, but the order of splicing is strongly influenced by length with longer introns tending to splice after shorter introns (24). These results were broadly consistent with studies that modulate transcription rate and measure effects on splicing (31). A purely kinetic model would predict that a slow polymerase would favor exon inclusion, however this was true only half the time. The remaining cases (i.e. unaffected or effected in the opposite direction) indicate that splicing events are heterogeneous and include non-transcriptional means of delaying intron processing to allow for downstream intron transcription (32).

To better understand these phenomena, introns were tested in isolation where their relative splicing efficiency was in general agreement with their relative order of splicing. Prior work from the Hertel lab has provided convincing evidence that the splicing of an intron is increased when coupled to the splicing of a downstream intron through an EJC-independent mechanism (13). More striking here was the inability of certain introns to be processed by themselves. Indeed, intron 49 of Col4A5 was almost completely dependent on the splicing of intron 48. Intron 49 falls within a typical size range and does not contain unusually weak splice sites. It is difficult to say how many introns lack the definition to splice with the fidelity observed *in vivo*. One approach was to identify ‘local slowpoke’ introns that splice after both their neighbors and test them in the single intron assay. We find significant overlap between local slowpoke introns and detained introns (*P* < 0.001, resampling) (33). Local slowpokes share some similarity to retained introns; however this study tested 10 local slowpokes and found 40% had compromised splicing suggesting a total number of 7200 cases of neighbor intron dependencies. This suggests certain introns require either the act of splicing or prior exposure to cis-elements in neighboring introns. While some introns appear to require neighboring splicing events for correct processing, it is not clear if this dependency is biologically important.

One function that was discovered was related to alternative splicing. Several kinetic studies of transcription and splicing have concluded that splicing must be delayed in the case of exon skipping. Splicing occurs rapidly and co-transcriptionally, often before the transcription of the downstream intron is complete. In substrates that undergo alternative splicing, both introns must be available to allow a selection between upstream and downstream splice sites. The *in vivo* order of splicing data exhibits a strong bias for both introns that flank the internal exon to splice later. Interestingly, this delay was also seen for transcripts that skip two exons. Here the skipping event encompasses three introns and the delayed splicing is most extreme in the middle intron and less so in the flanking introns. This is in contrast to previous explanations of a two exon skipping event in the COL5A1 gene which find the middle intron (intron 5) to be spliced rapidly. Possible explanations for the difference include different cell lines and the fact that COL5A1 study is performed on a splicing mutant to explain an aberrant splicing event (34).

Finally, cryptic 3′ss usage was strongly associated with retrograde splicing (i.e. the removal of downstream introns prior to upstream introns). Why cryptic 3′ss substrates are associated with the prior splicing of a downstream intron when cryptic 5′ss are similar to constitutive splicing events was not immediately clear. It is possible to speculate that the lack of definition at the 3′ss requires additional enhancement from a prior splicing event. However, a more definitive explanation would require further study. This report finds that first and last introns are likely to be spliced after their more internal neighbor. This observation is consistent with the finding that most introns splice co-transcriptionally however the first and last introns are less likely than internal introns to be removed co-transcriptionally (35).

## Supplementary Material

Supplementary Data
