# Supplementary material for: Widespread intra-dependencies in the removal of introns from human transcripts
Source: Nucleic Acids Res. 2017 Jul 29;45(16):9503–13. doi: 10.1093/nar/gkx661 (PMC5766209; doi:10.1093/nar/gkx661)
Supplement: Supplementary Data [file gkx661_supp.pdf]

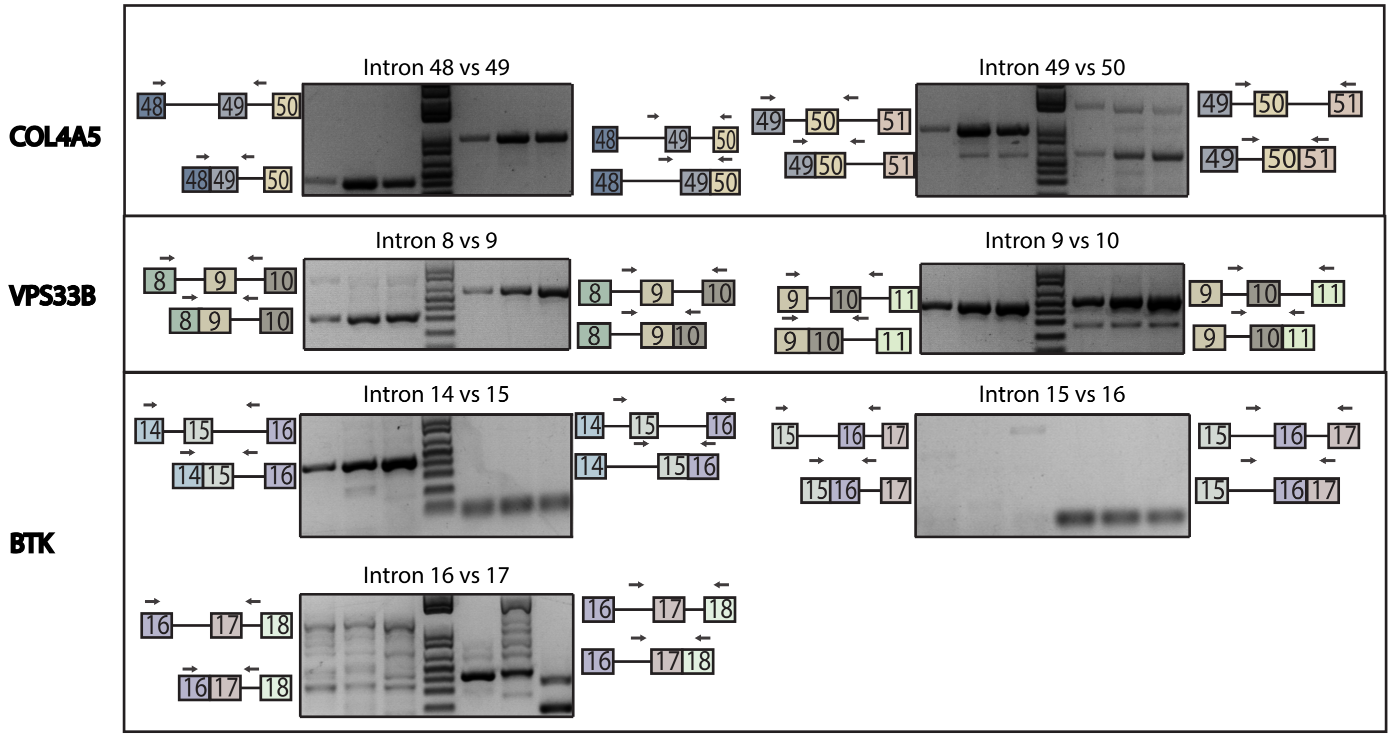

Supplementary Fig S1. Three genes containing HGMD mutations that induce multiple exon skipping. RT-PCR results determining the relative order of intron removal. Primers were designed to visualize all potential intermediates, design similar to (14).

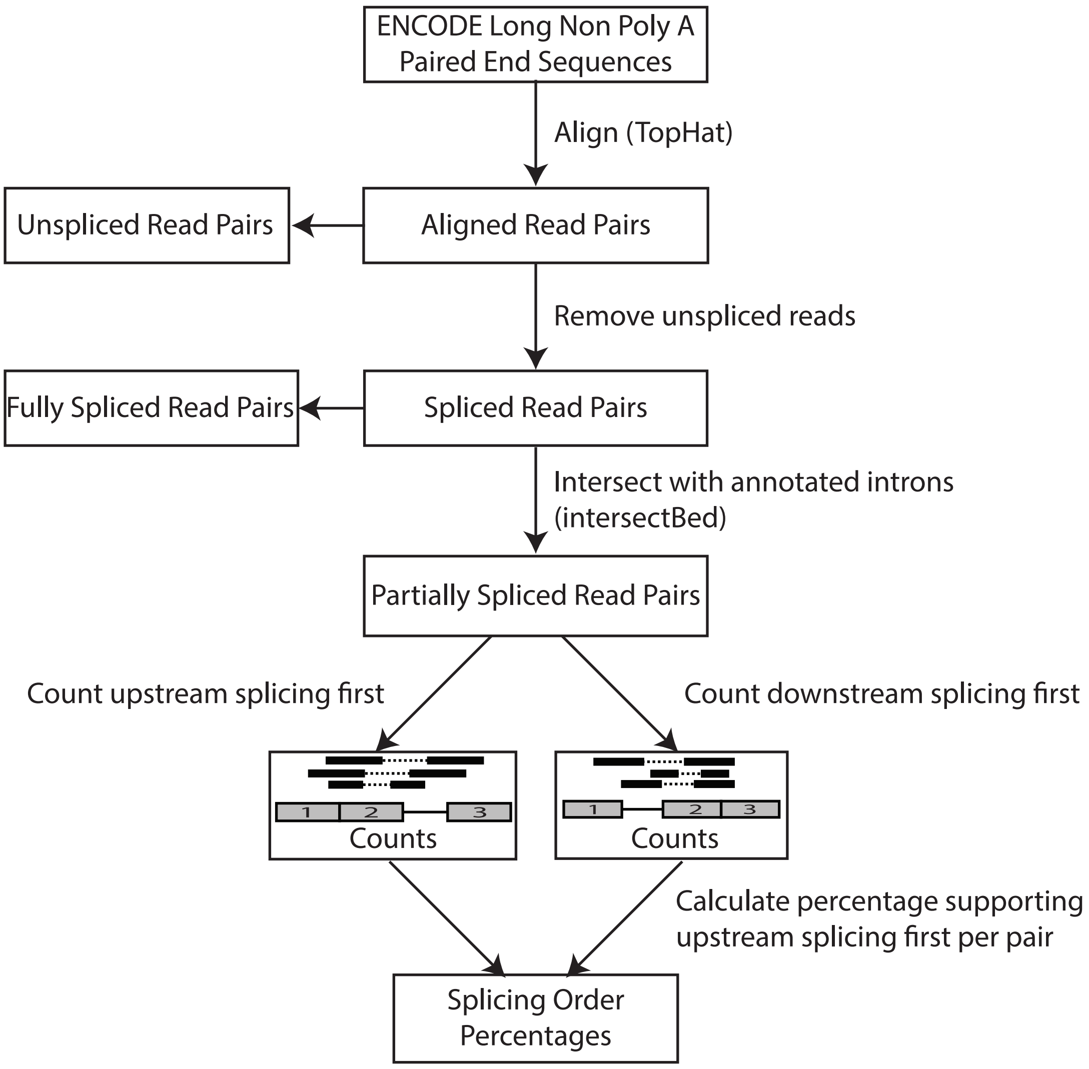

Supplementary Fig S2. Pipeline used to discover order of intron removal using paired end sequencing reads. ENCODE paired end RNAseq reads were screened for partially spliced intermediates, in which one end of the paired end read mapped to an unspliced intron, and the other spanned a spliced exon/exon junction. The number of read counts supporting the upstream intron splicing first vs the downstream intron were counted to calculate pairwise order of splicing data.

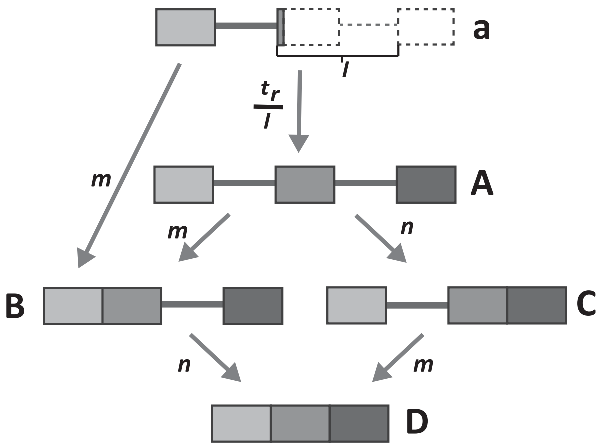

Let a, A, B, C, and D denote the concentration of each species. B and C are transient intermediate species that concentration is in steady state. *m* and *n* denote the reaction coefficient of each splicing step, which are taken from previous literature. t_r/l (transcription rate / length from upstream 3'ss to downstream 3'ss) denotes the rate at which a partially transcribed transcript is transcribed past the downstream 3'ss (species a is transcribed to species A). Here a purely kinetic model is used. Splicing rate only depends on the length of each intron and concentration of reactant. With this model, change of A, B, C, D over time can be described by following equations:

$$\frac{da}{dt} = c_r - ma - \frac{t_r}{l}a = 0 \quad \text{[Eq.1]}$$

$$\frac{dA}{dt} = \frac{t_r}{l}a - mA - nA = 0 \quad \text{[Eq.2]}$$

$$\frac{dB}{dt} = mA + ma - nB = 0 \quad \text{[Eq.3]}$$

$$\frac{dC}{dt} = nA - mC = 0 \quad \text{[Eq.4]}$$

$$\frac{dD}{dt} = nB + mC - d = 0 \quad \text{[Eq.5]}$$

Since we assumed the steady state for the intermediates B and C, using Equations 3 and 4, B and C can be described as the function of A and the reaction rate coefficients, *m* and *n*.

$$B = \frac{m}{n}(A + a) \quad \text{[Eq.6]}$$

$$C = \frac{n}{m}A \quad \text{[Eq.7]}$$

Simarly, assuming steady state conditions for premRNA molecules a and A using Equations 1 and 2, a and A can be described as functions of premRNA creation rate, transcription rate, length, and the reaction rate coefficients, *m* and *n*.

$$a = \frac{c_r}{m + \frac{t_r}{l}} \qquad \text{[Eq.8]}$$

$$A = \frac{a t_r}{l(m+n)} \qquad \text{[Eq.9]}$$

Using these equations, the fraction of upstream intron being removed first can be calculated as a function of *m*, *n*, t_r and *l*.

$$\frac{upstream\ first}{total\ intermediates} = \frac{B}{B+C} = \frac{\frac{m}{n}(A+a)}{\frac{m}{n}(A+a)+\frac{n}{m}A} = \frac{m^2(A+a)}{m^2(A+a)+An^2}$$

$$\frac{m^2(A+a)}{m^2(A+a)+An^2} = \frac{m^2\left[\frac{c_r}{m+{}^{t_r}/_{l}}+\frac{c_r t_r}{\left(m+{}^{t_r}/_{l}\right)(l(m+n))}\right]}{m^2\left[\frac{c_r}{m+{}^{t_r}/_{l}}+\frac{c_r t_r}{\left(m+{}^{t_r}/_{l}\right)(l(m+n))}\right]+n^2\left[\frac{c_r t_r}{(m+{}^{t_r}/_{l})(l(m+n))}\right]}$$

$$\frac{upstream\ first}{total\ intermediates} = \frac{m^2(m+n+{}^{t_r}/_{l})}{m^2(m+n+{}^{t_r}/_{l})+n^2({}^{t_r}/_{l})} \qquad \text{[Eq.10]}$$

In the case of no co-transcriptional splicing, where the rate of transcription is much higher than splicing rates, the fraction of upstream intron being removed first can be calculated as a function of just *m* and *n*.

$$if\ {}^{t_r}/_{l} \gg m+n:$$

$$\frac{upstream\ first}{total\ intermediates} = \frac{m^2}{m^2+n^2}$$

[Eq.10]

Supplementary Fig S3. Derivation of kinetic model of the percentage of time the upstream intron splices first.

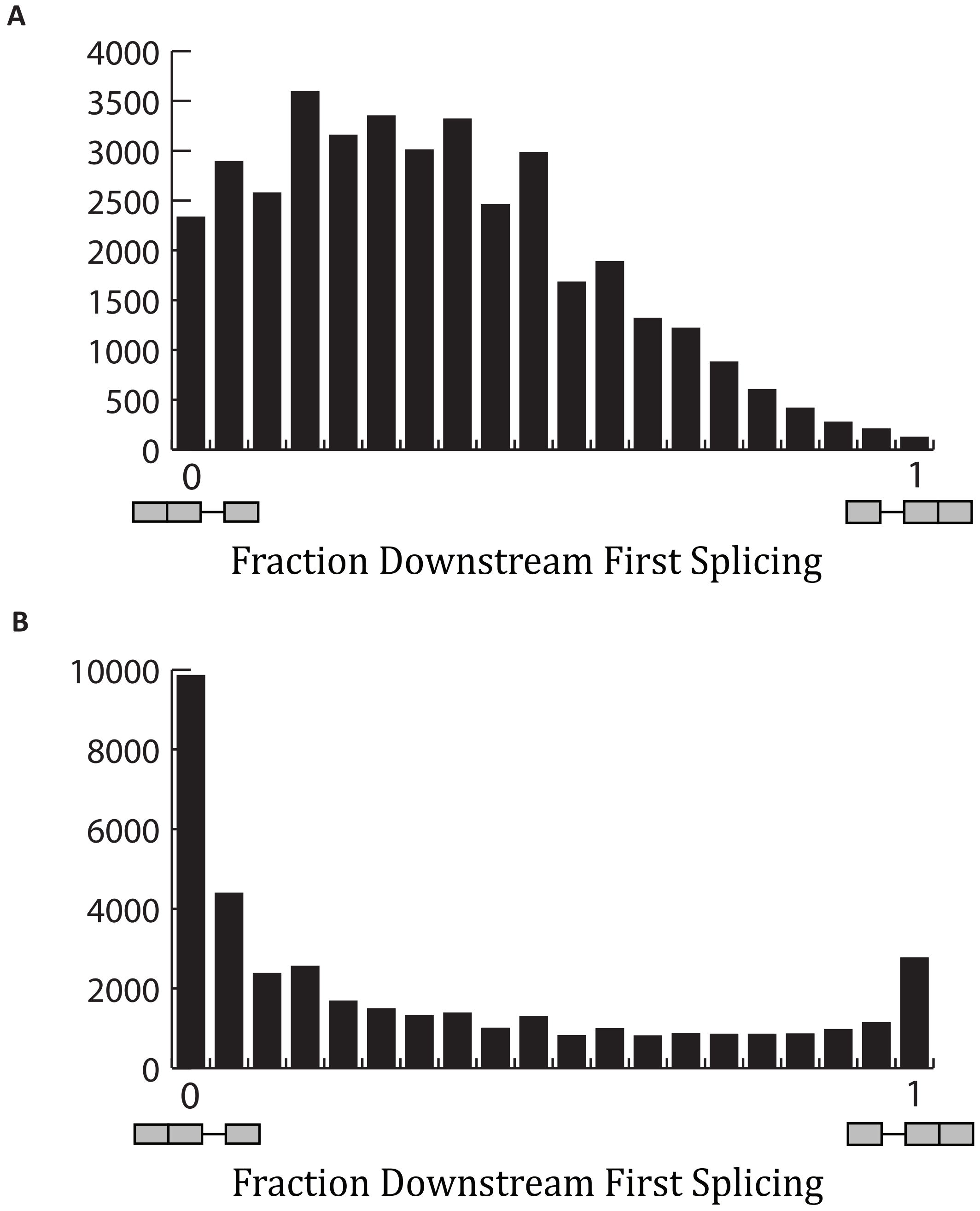

Supplementary Fig S4. Simulation with observed splicing rate variance and 10x elevated variance. Splicing and transcription rates were modeled as normally distributed constants bounded at zero. Read coverage at each pair of introns was preserved A) Simulation using the variance published in (20) was performed as described. Introns were binned according to the degree of upstream intron splicing first. B) Simulation increasing the variance 10x.

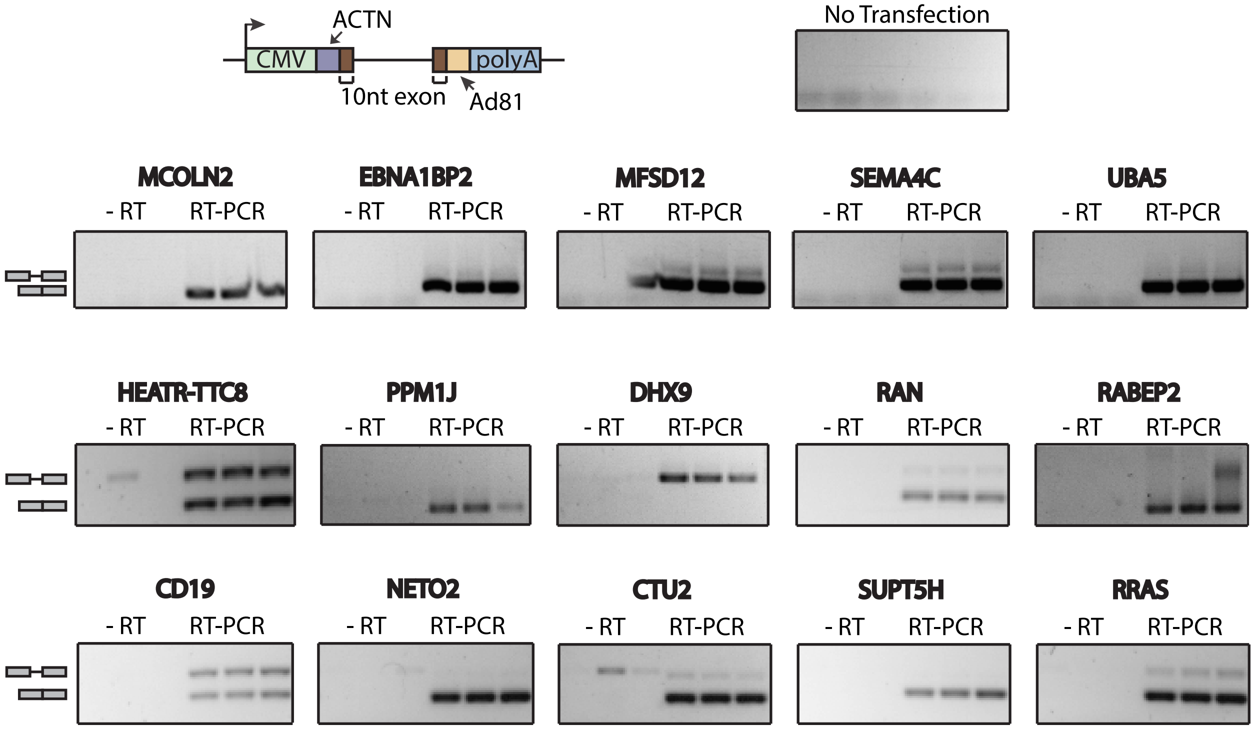

Supplementary Fig S5. Slower splicing introns are more likely to show lower splicing efficiency in a single intron minigene construct. Top row – five introns that splice first > 90% of the time. Middle and bottom row – ten introns that always splice last among neighbors.

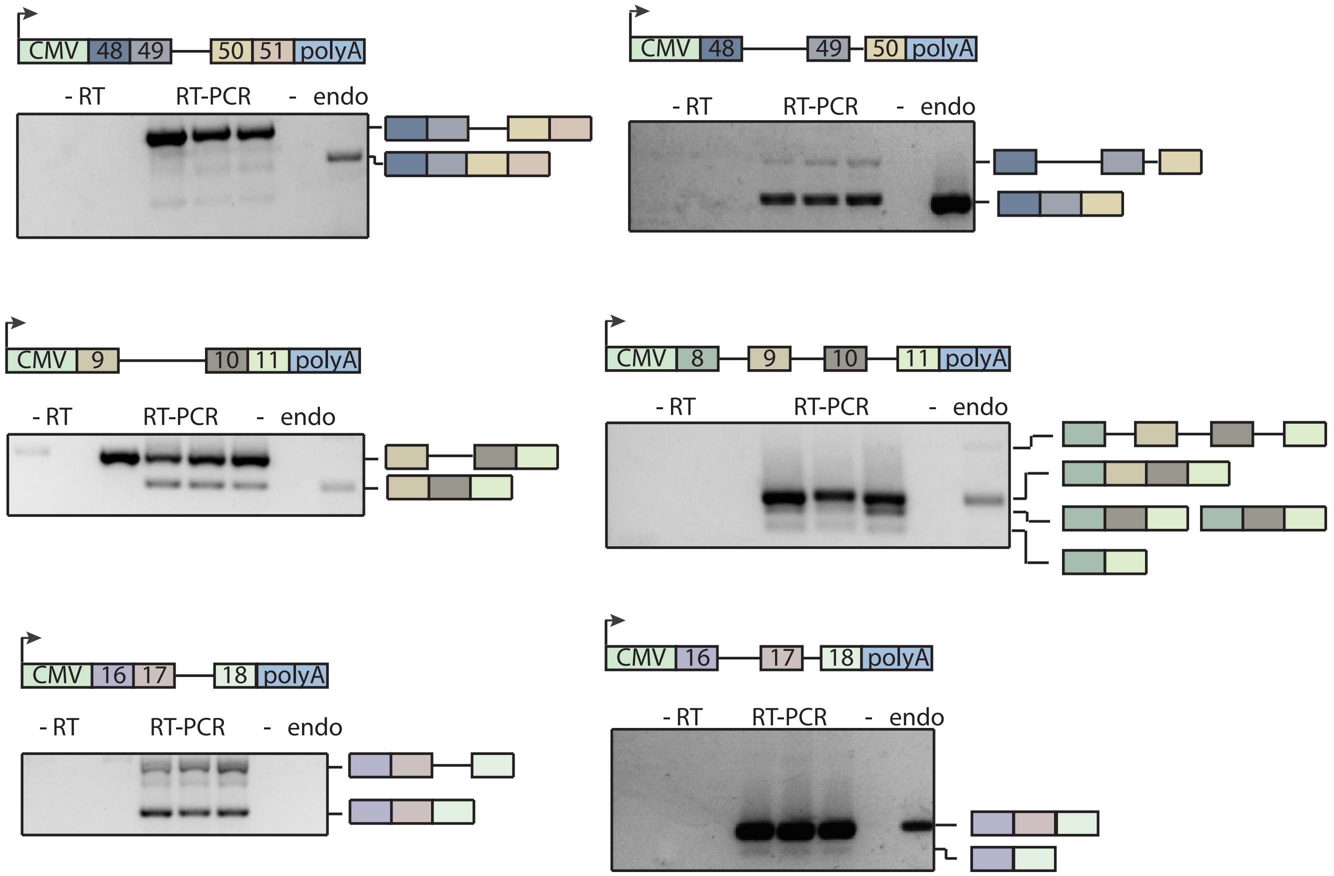

Supplementary Fig S6. The splicing reaction enhances subsequent splicing of neighbor introns. Various portions of neighbor sequences (spliced exonic and unspliced intronic sequences) were included in the minigene construct to determine effect on splicing efficiency.

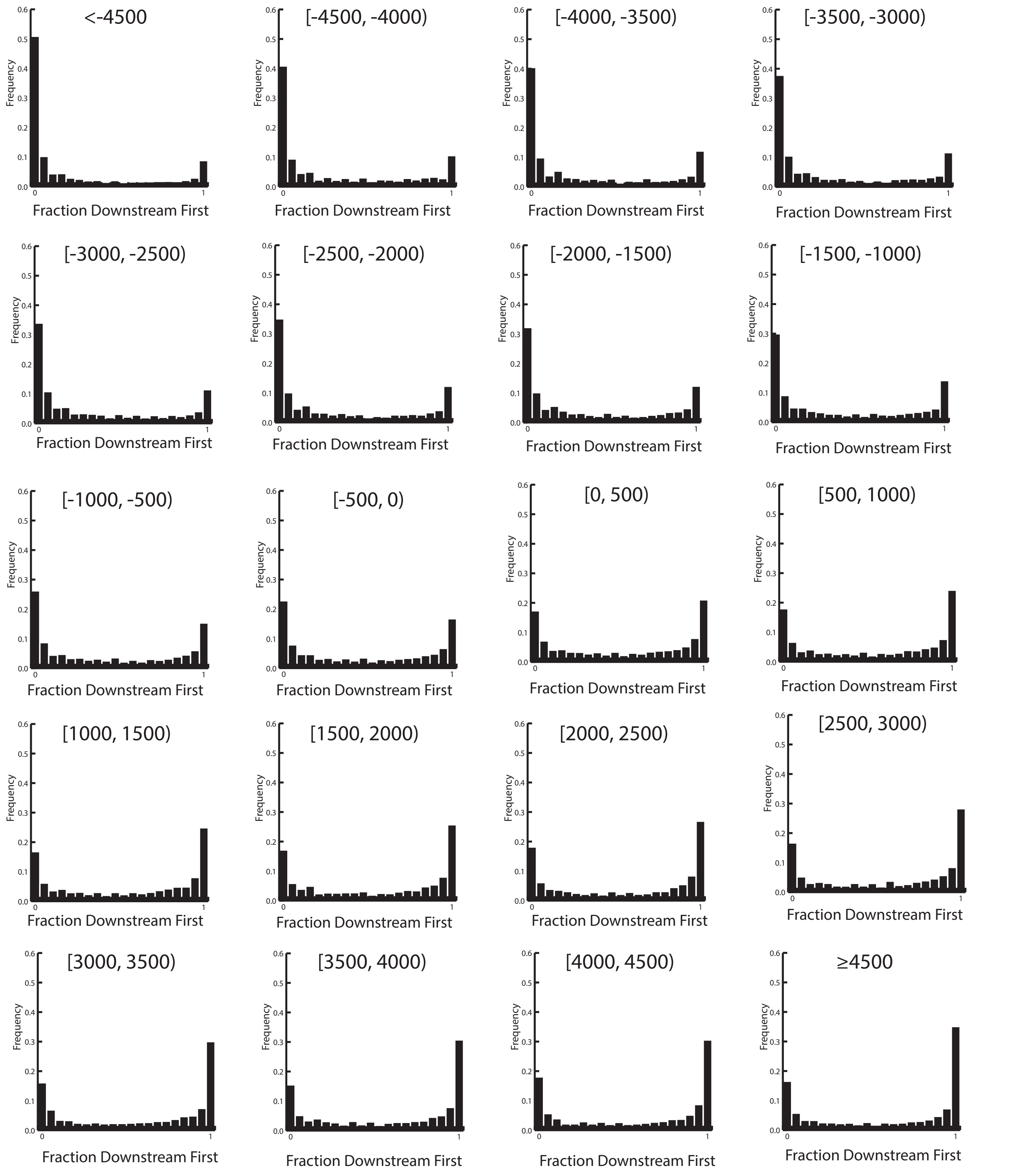

Supplementary Fig S7. Distribution of downstream first splicing fraction for a given range of intron pair length differences. The intron pair length difference is defined as *length of upstream intron – length of downstream intron*.

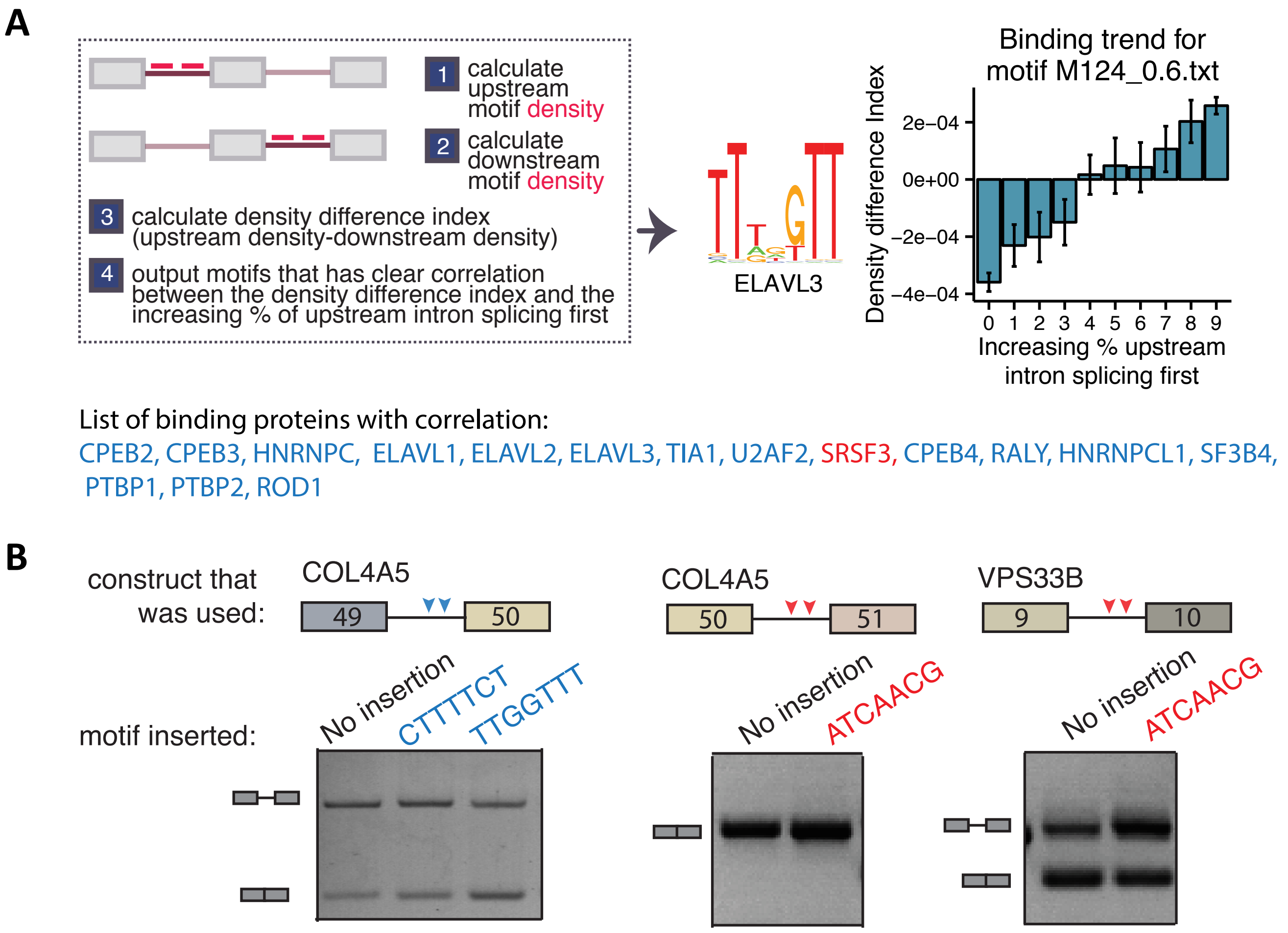

Supplementary Fig S8. U-rich Motifs enriched in always-first splicing introns can increase splicing efficiency in minigene. (a) Methods to find motifs enriched in the always-first or always-last splicing introns. Most of motifs that are enriched in always-first splicing introns were U-rich motifs. There was only one motif that is enriched in always-last splicing intron. (b) Two motifs enriched in the always-first splicing introns were selected and one motif enriched in the always-last splicing introns were inserted at -45nt and -50nt position of the single intron constructs.

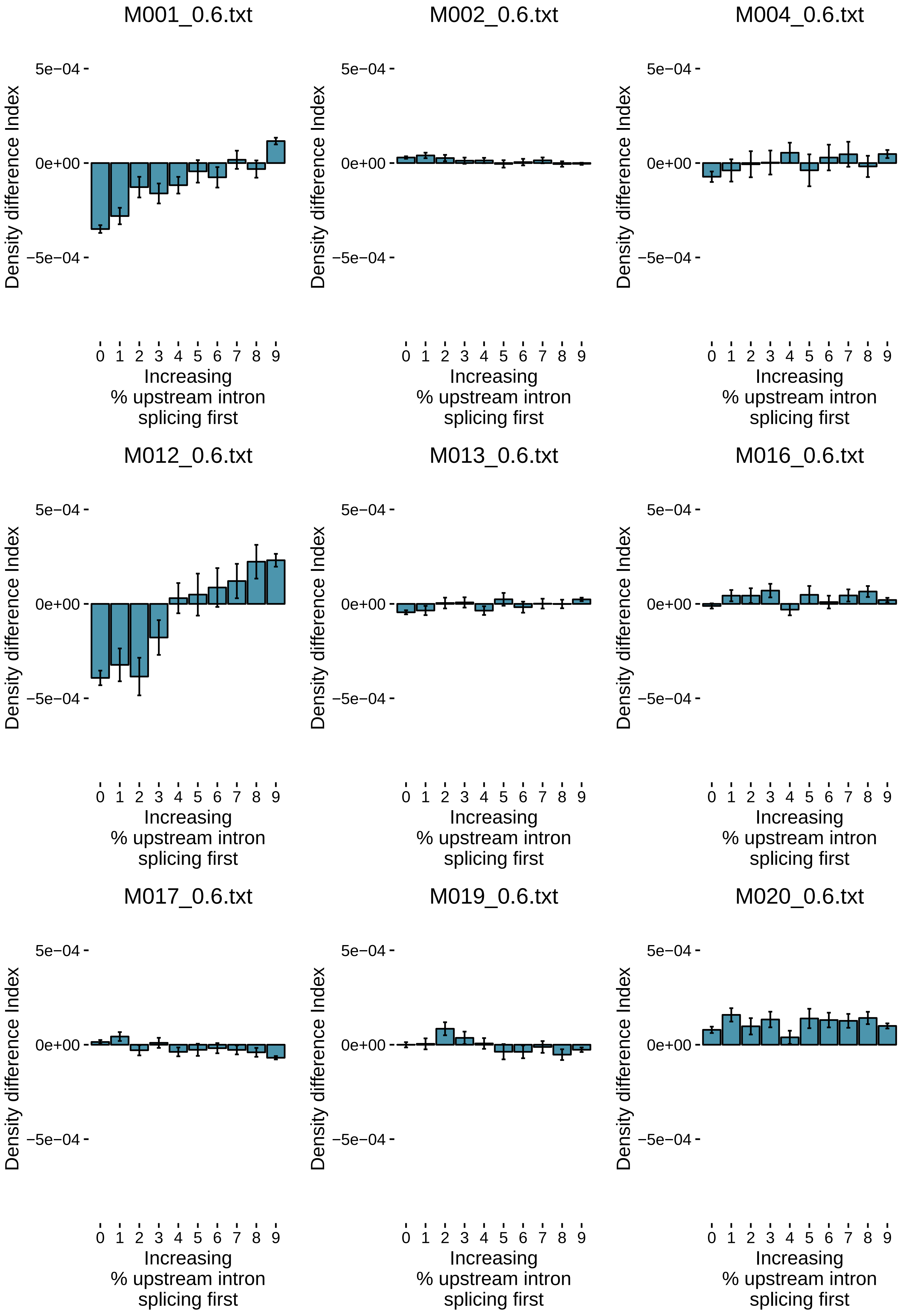
M001_0.6.txt
M002_0.6.txt
M004_0.6.txt
M012_0.6.txt
M013_0.6.txt
M016_0.6.txt
M017_0.6.txt
M019_0.6.txt
M020_0.6.txt
Density difference Index
5e−04
0e+00
−5e−04
0 1 2 3 4 5 6 7 8 9
Increasing
% upstream intron
splicing first

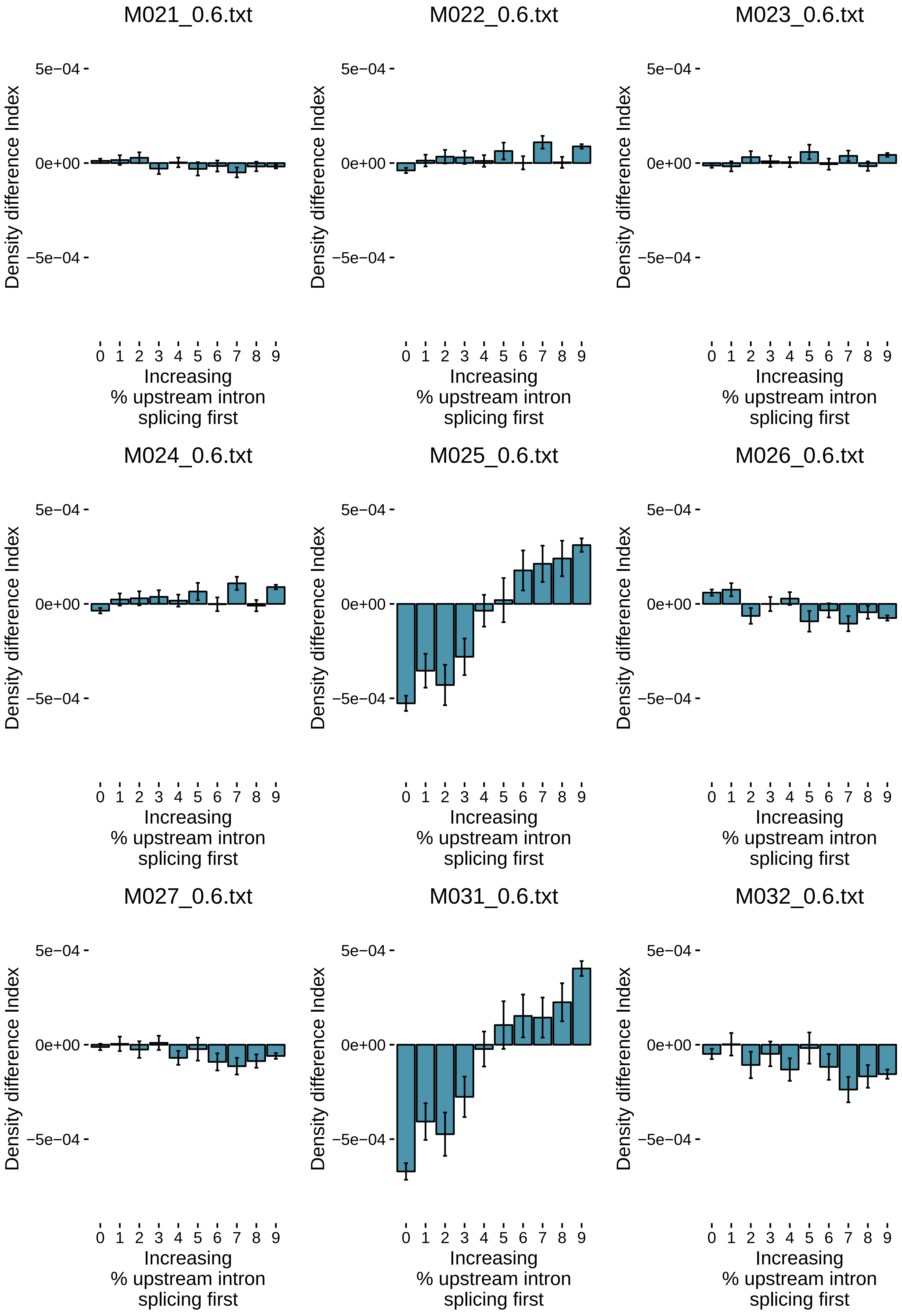
M021_0.6.txt
M022_0.6.txt
M023_0.6.txt
M024_0.6.txt
M025_0.6.txt
M026_0.6.txt
M027_0.6.txt
M031_0.6.txt
M032_0.6.txt
Density difference Index
5e−04
0e+00
−5e−04
0 1 2 3 4 5 6 7 8 9
Increasing
% upstream intron
splicing first

M033_0.6.txt

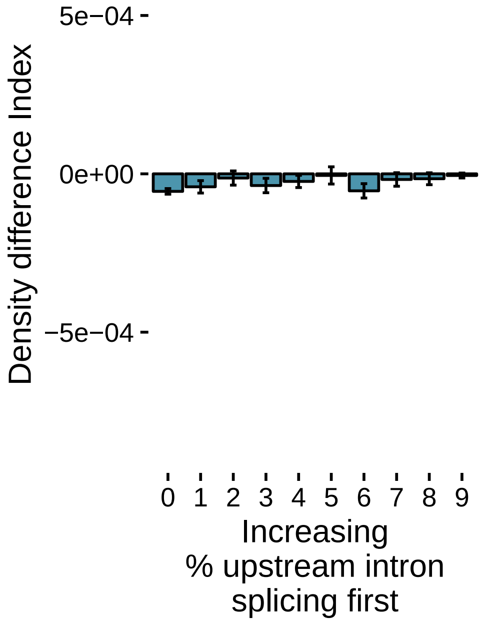

M035_0.6.txt

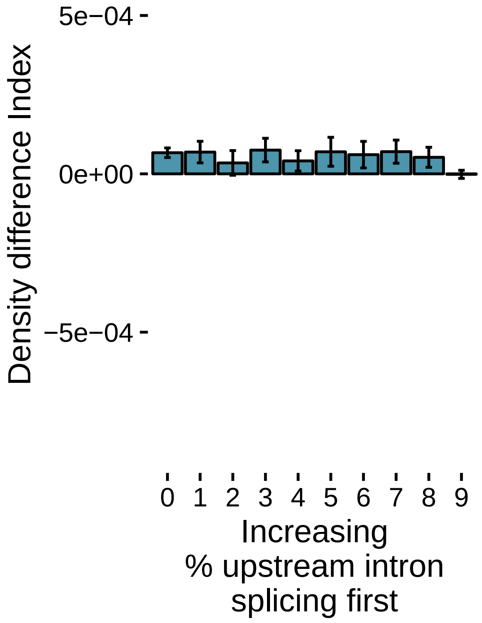

M036_0.6.txt

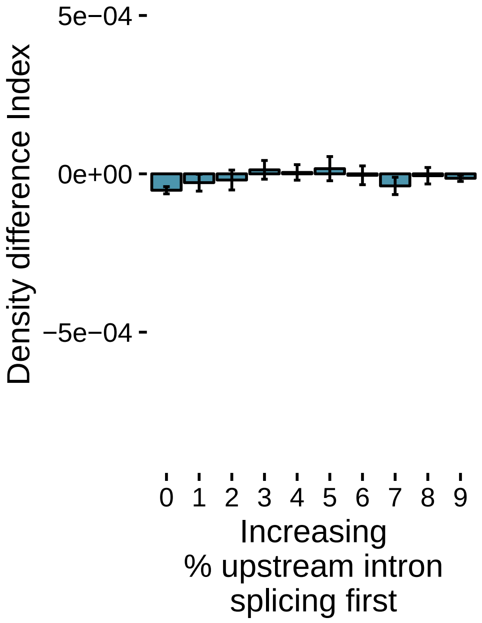

M037_0.6.txt

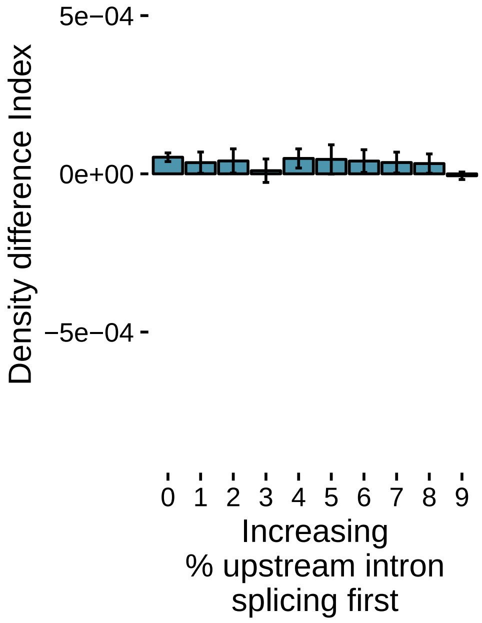

M040_0.6.txt

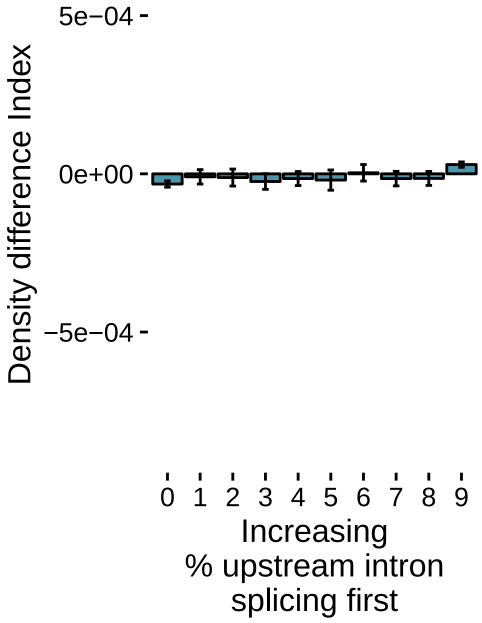

M042_0.6.txt

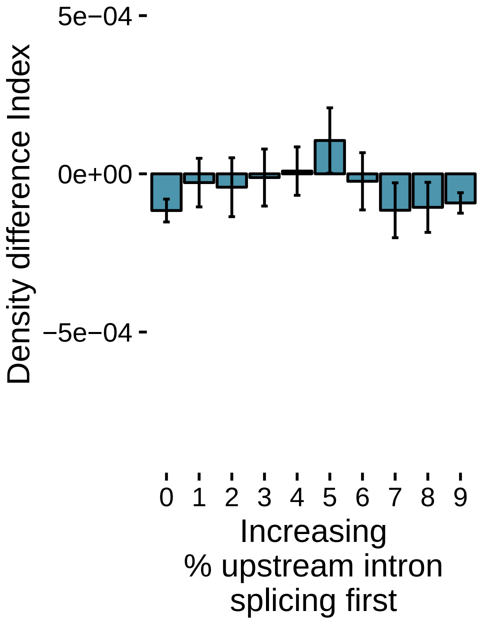

M043_0.6.txt

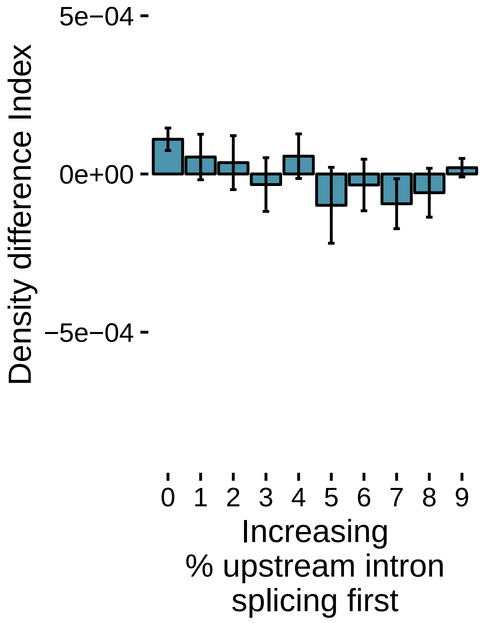

M044_0.6.txt

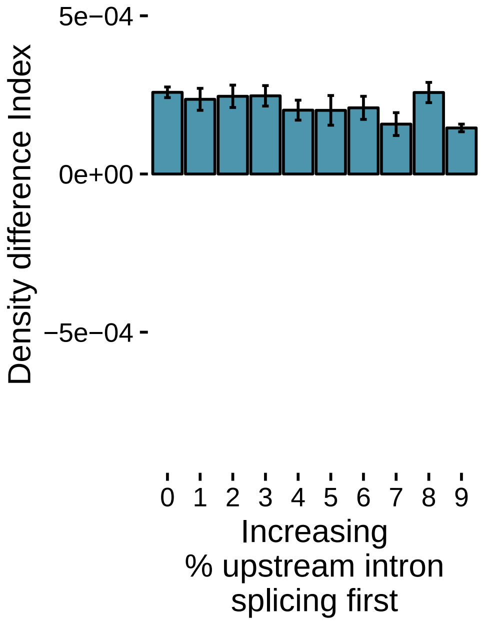

M046_0.6.txt

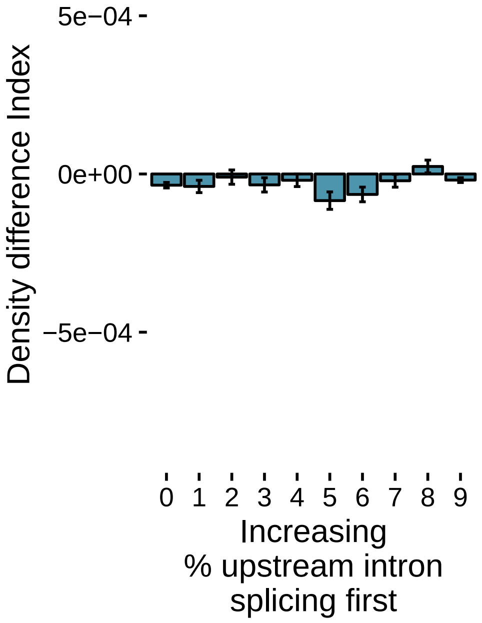

M047_0.6.txt

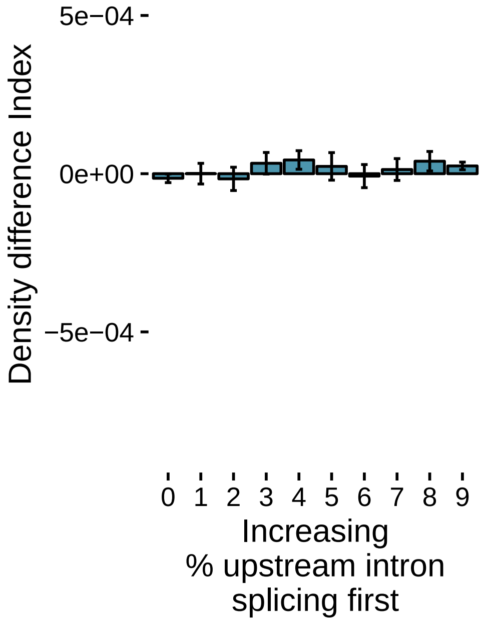

M048_0.6.txt

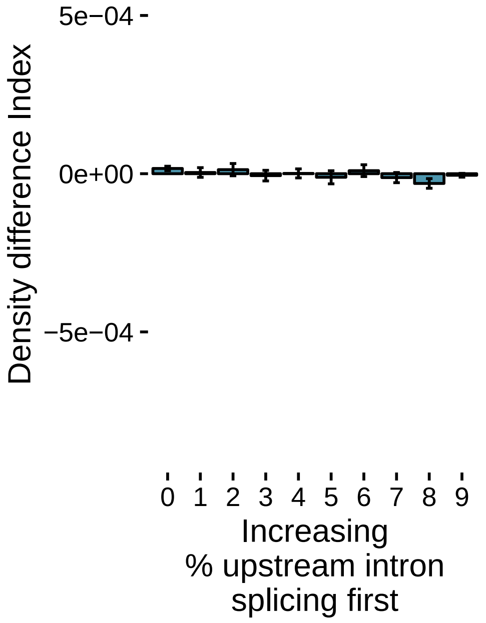

M049_0.6.txt

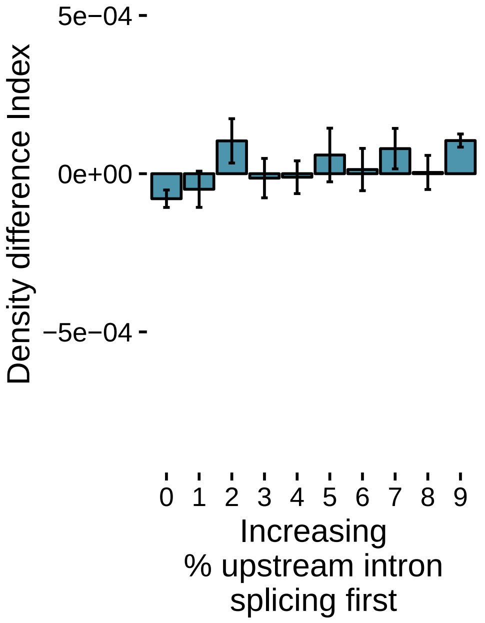

M050_0.6.txt

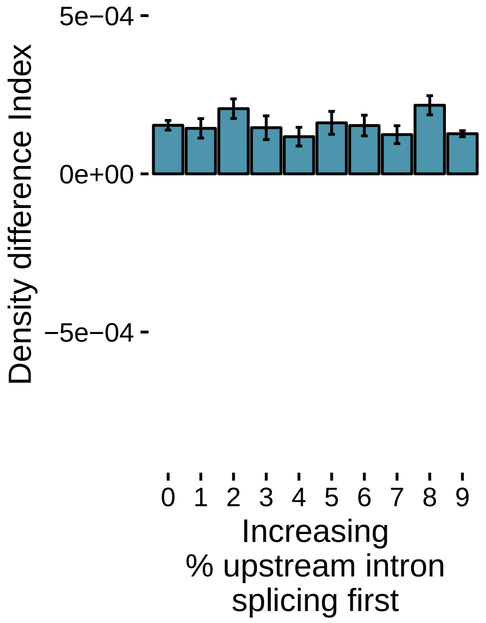

M051_0.6.txt

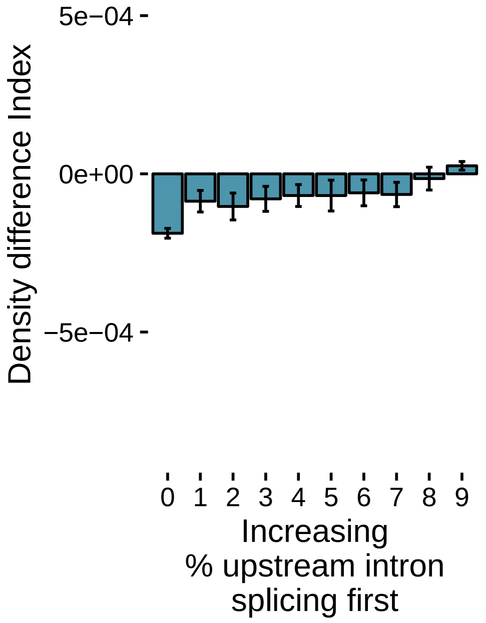

M052_0.6.txt

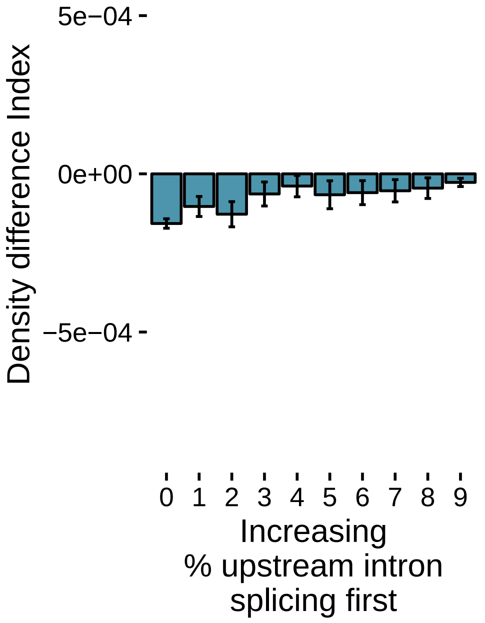

M053_0.6.txt

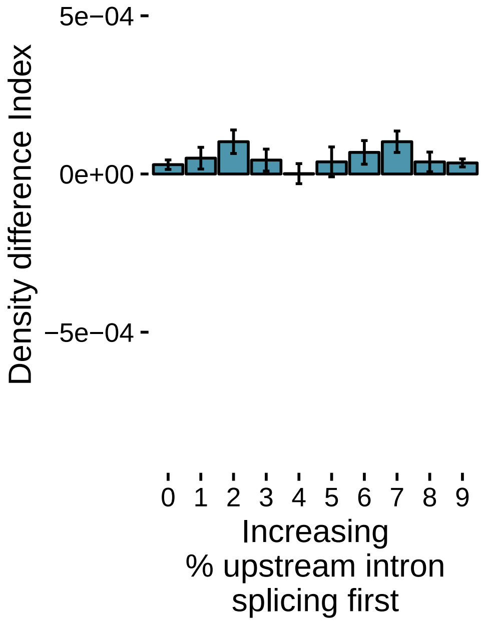

M054_0.6.txt

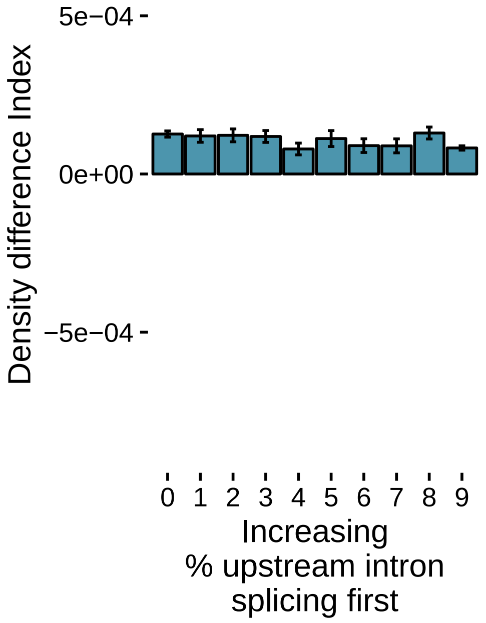

M055_0.6.txt

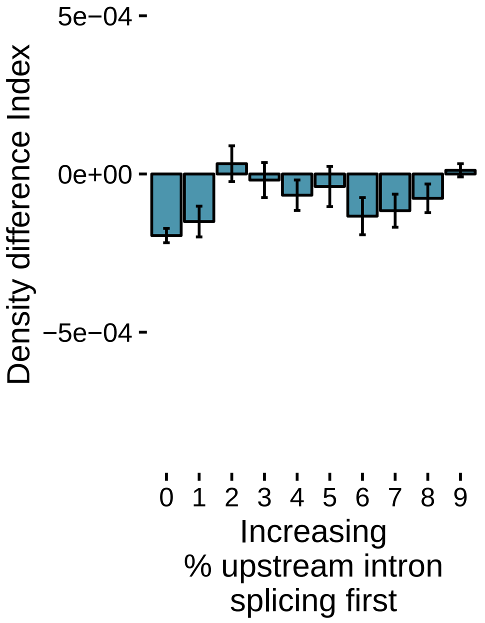

M056_0.6.txt

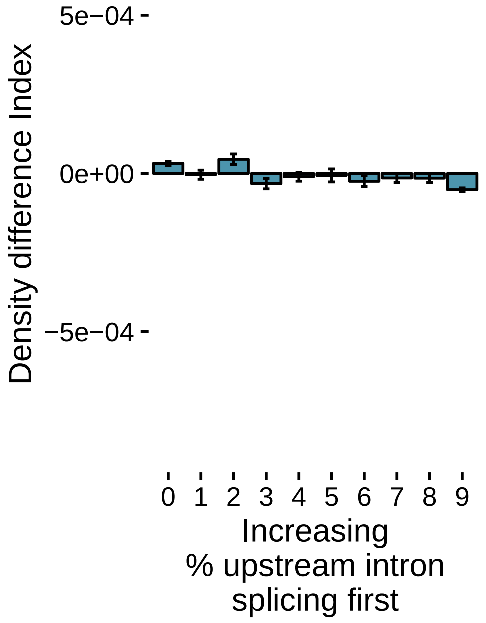

M061_0.6.txt

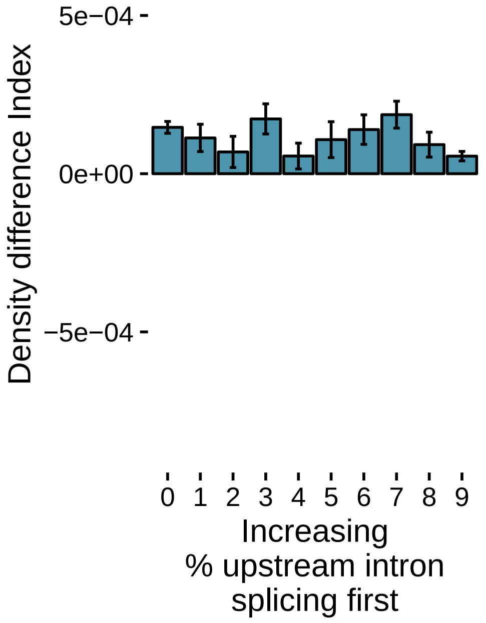

M062_0.6.txt

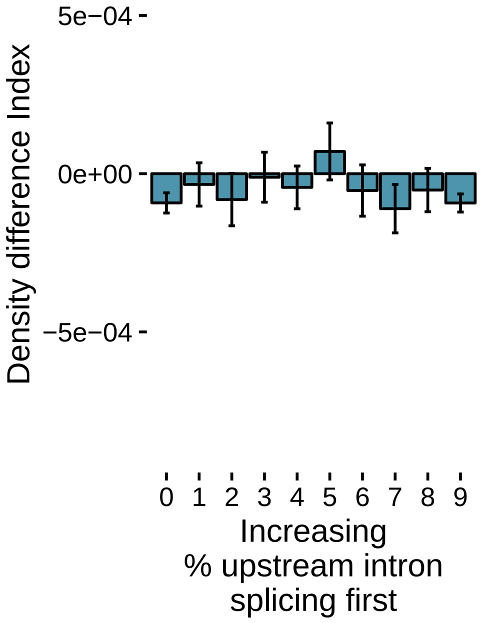

M065_0.6.txt

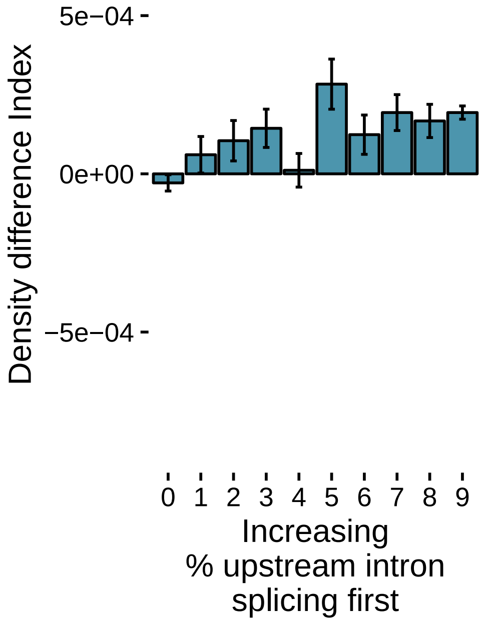

M068_0.6.txt

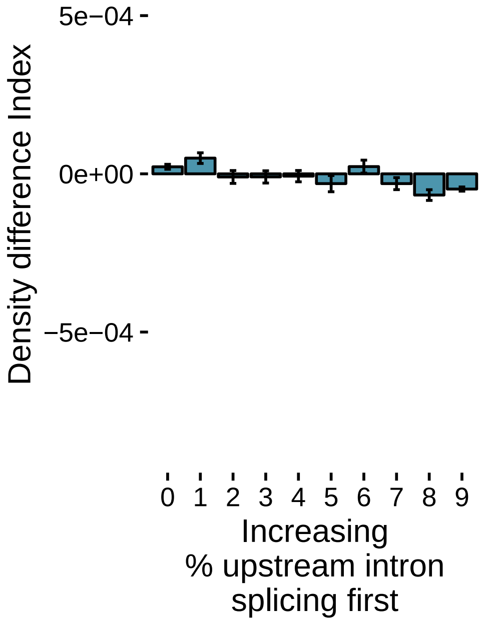

M069_0.6.txt

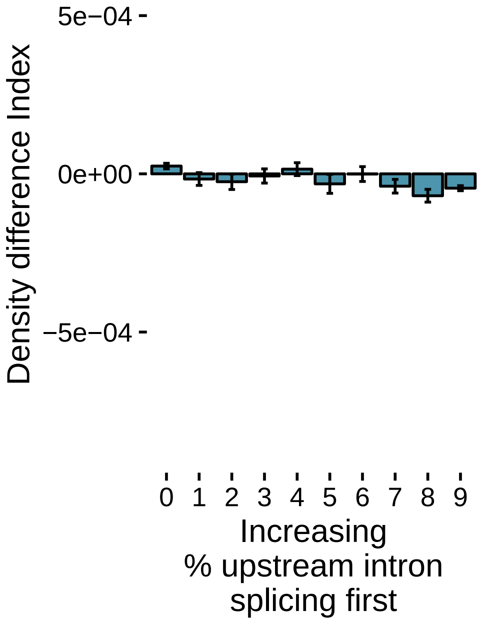

M070_0.6.txt

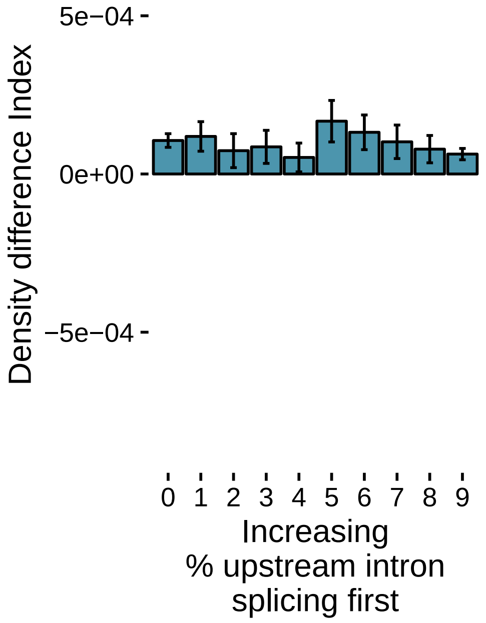

M071_0.6.txt

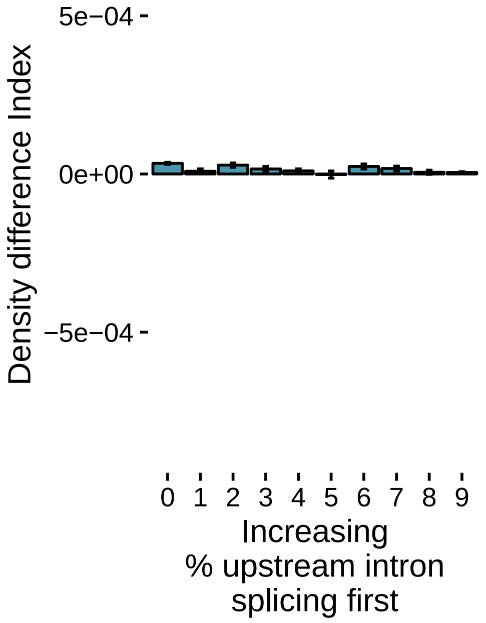

M072_0.6.txt

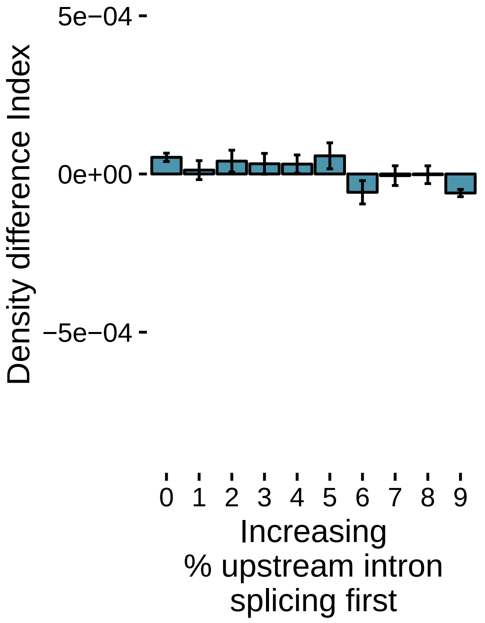

M073_0.6.txt

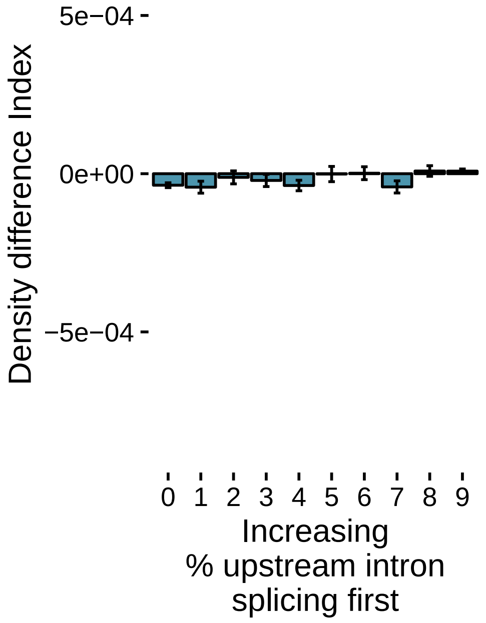

M074_0.6.txt

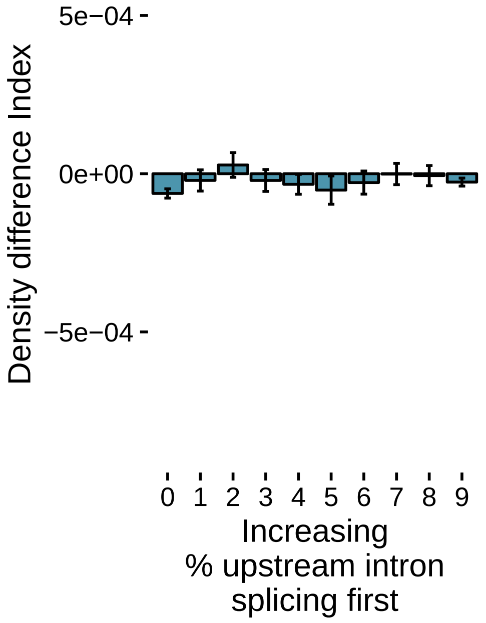

M075_0.6.txt

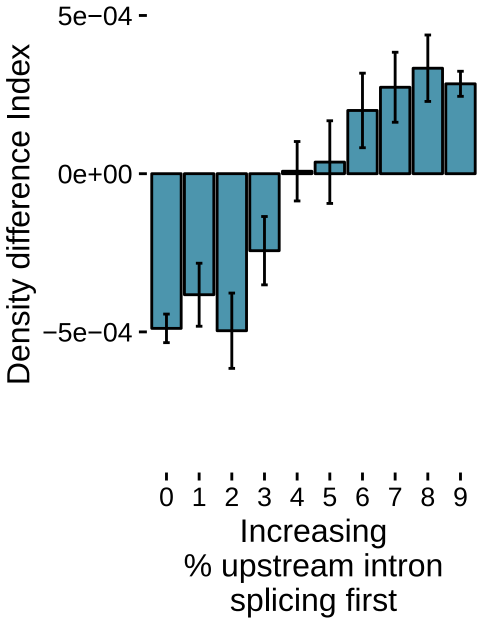

M077_0.6.txt

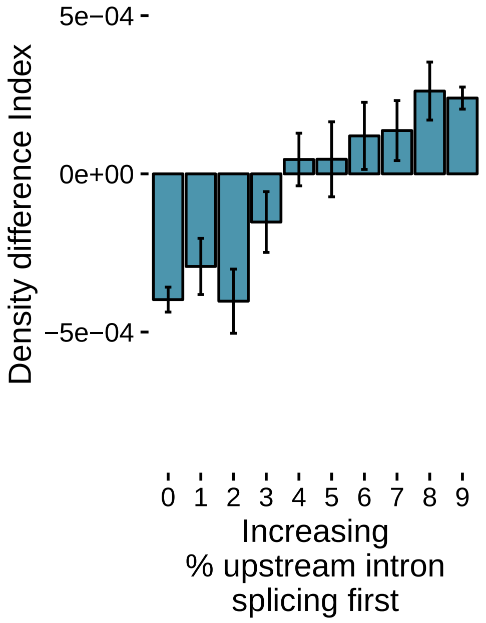

M079_0.6.txt

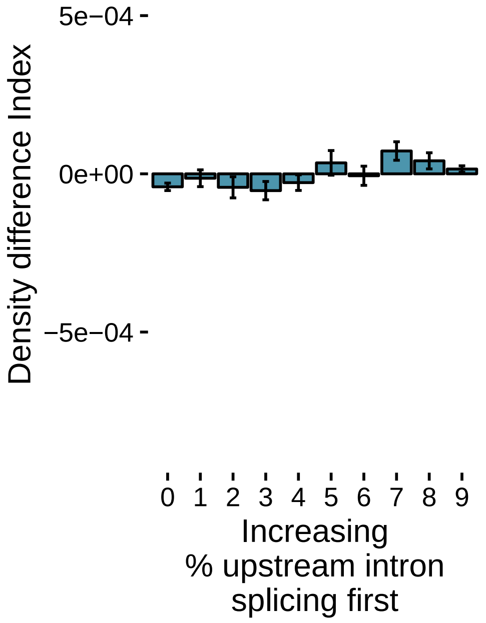

M081_0.6.txt

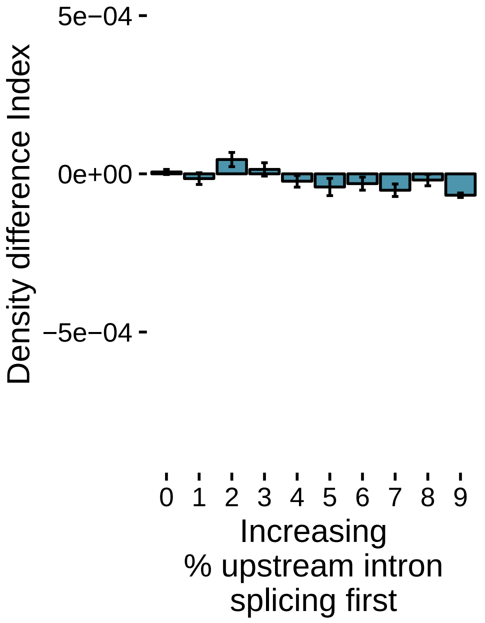

M082_0.6.txt

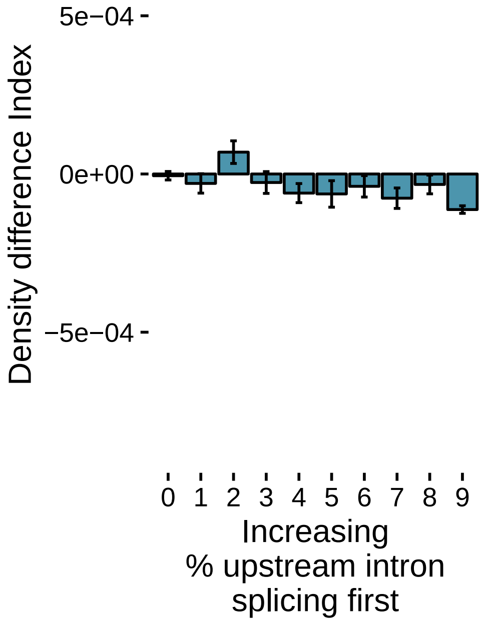

M083_0.6.txt

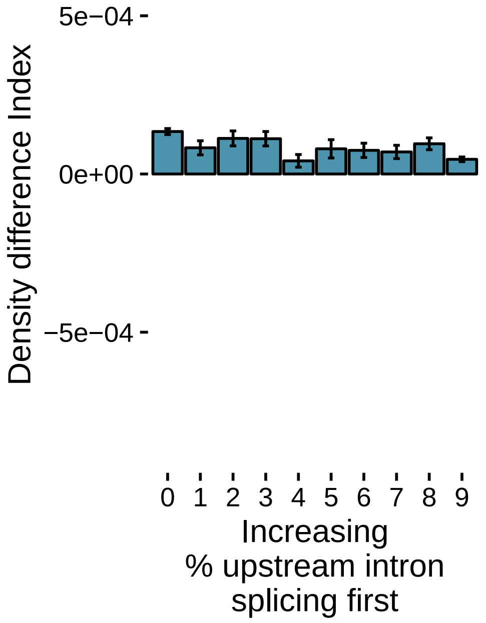

M085_0.6.txt

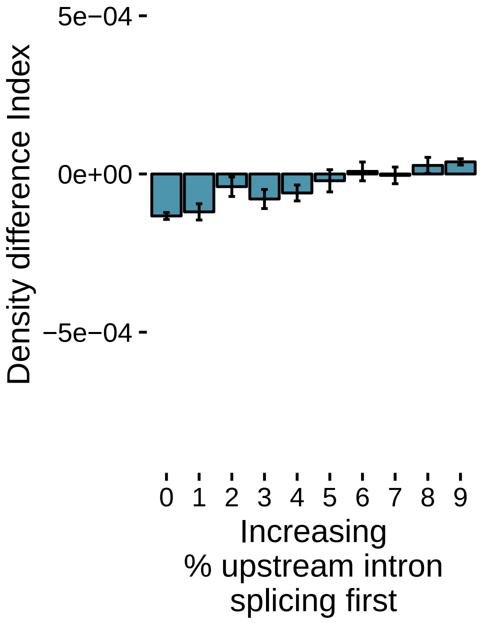

M086_0.6.txt

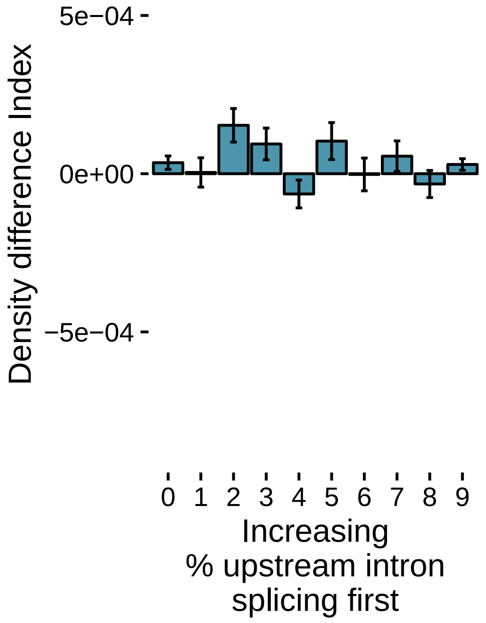

M087_0.6.txt

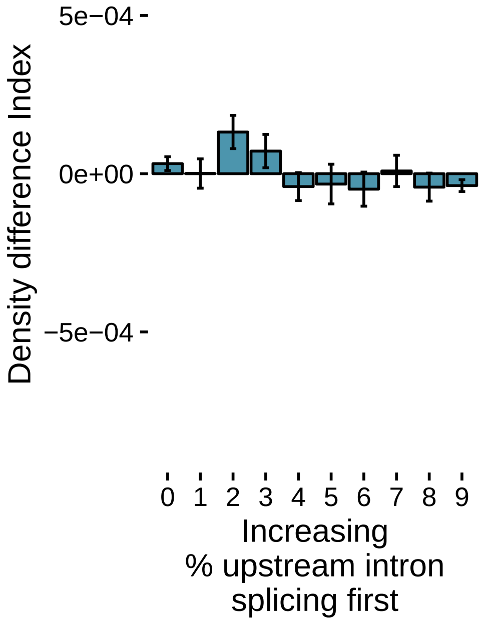

M088_0.6.txt

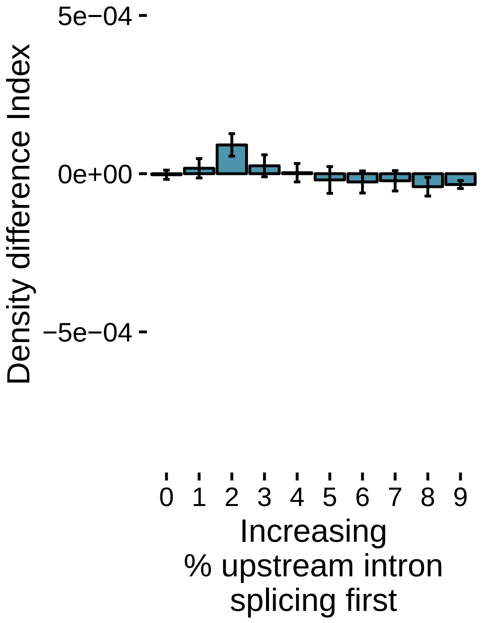

M089_0.6.txt

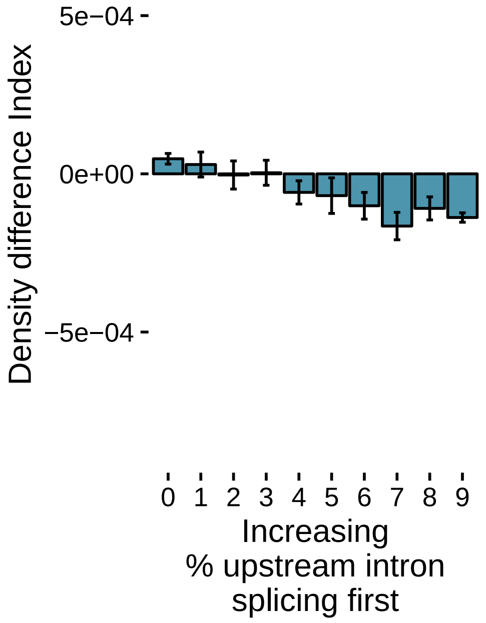

M102_0.6.txt

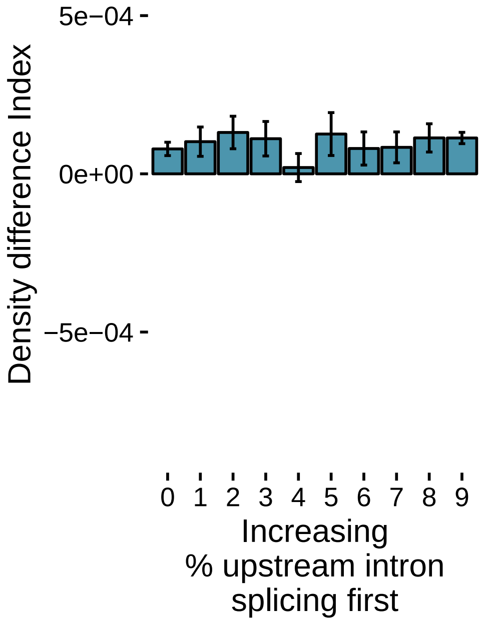

M103_0.6.txt

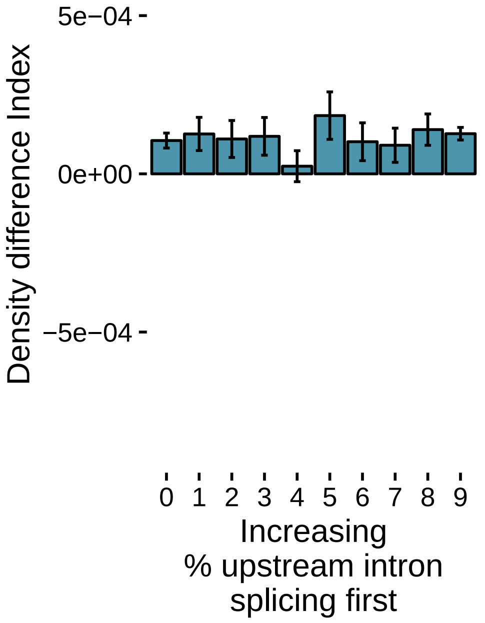

M104_0.6.txt

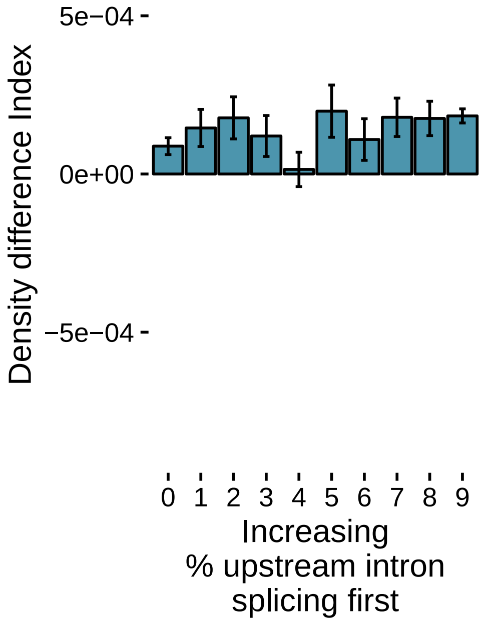

M105_0.6.txt

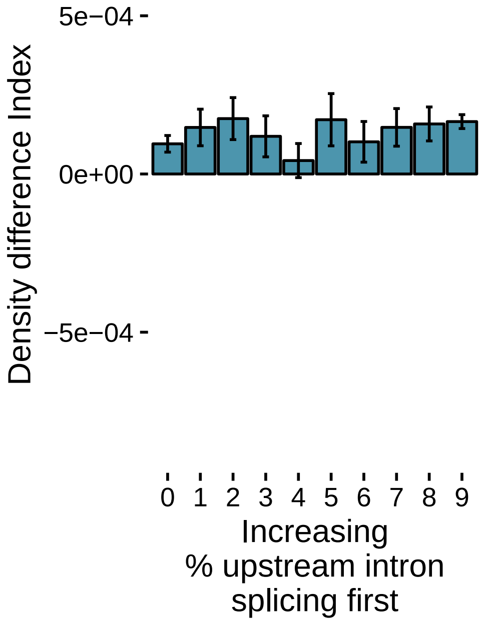

M106_0.6.txt

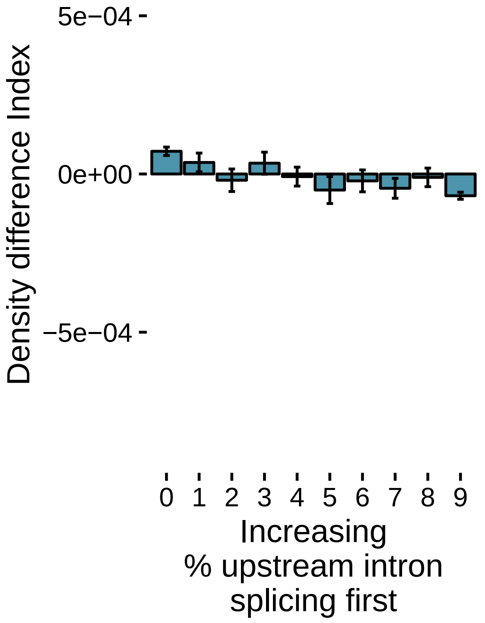

M108_0.6.txt

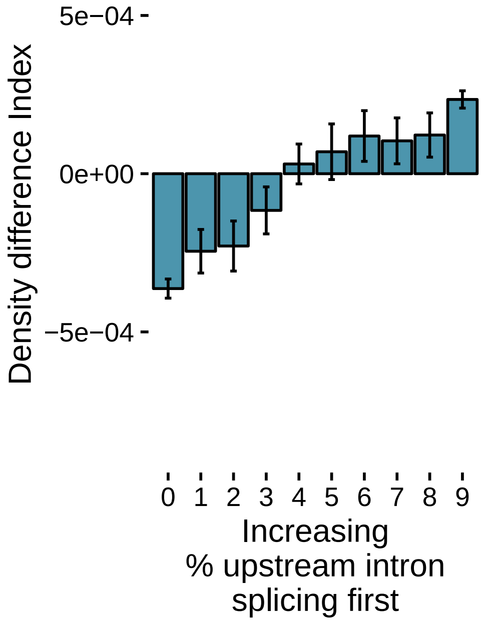

M109_0.6.txt

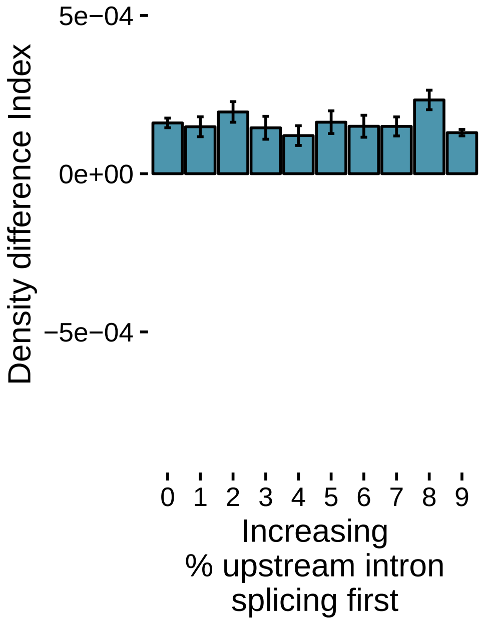

M111_0.6.txt

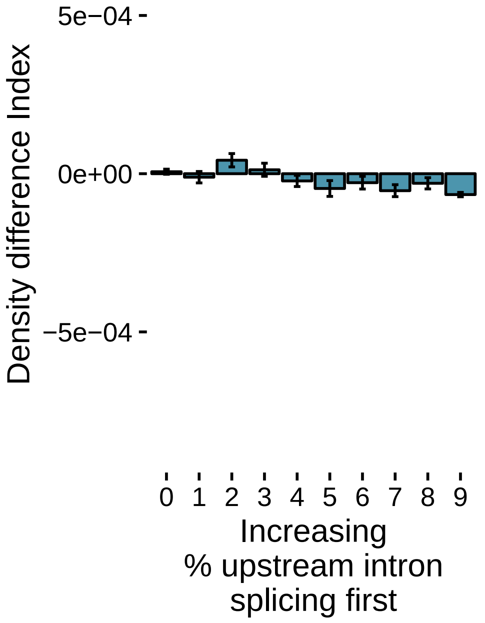

M112_0.6.txt

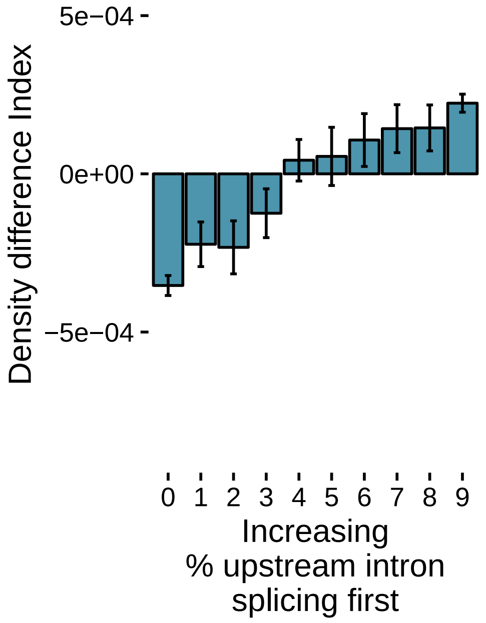

M118_0.6.txt

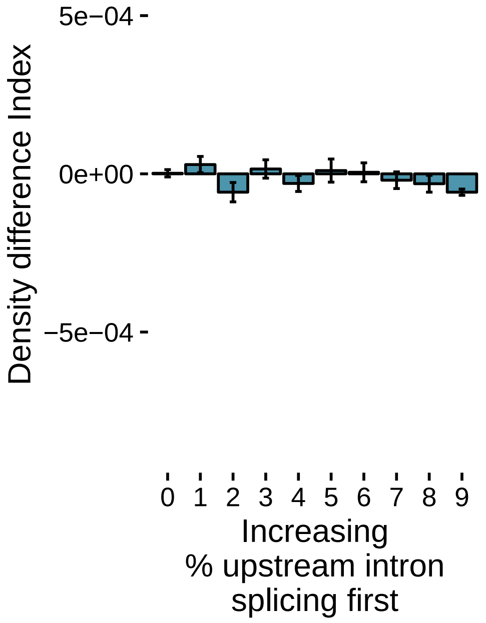

M120_0.6.txt

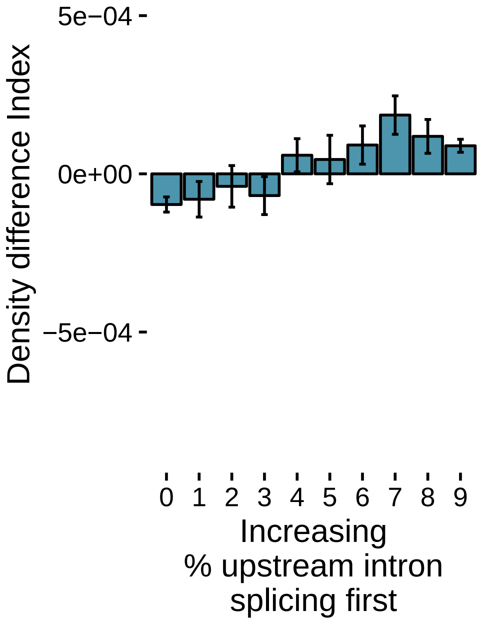

M121_0.6.txt

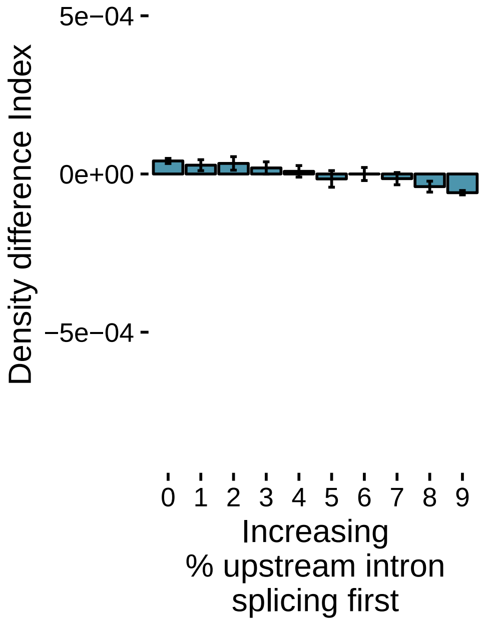

M122_0.6.txt

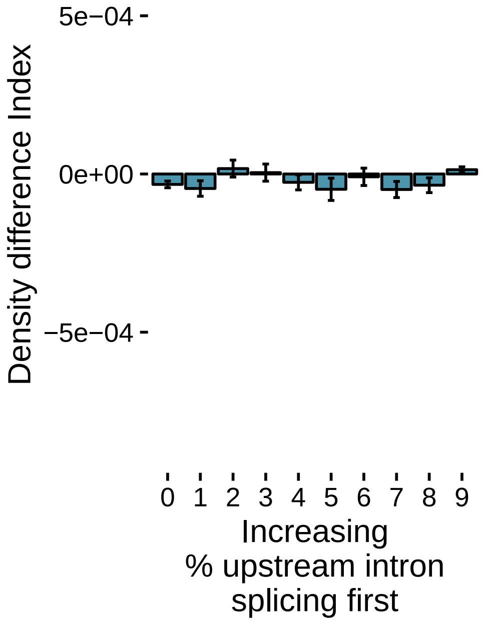

M124_0.6.txt

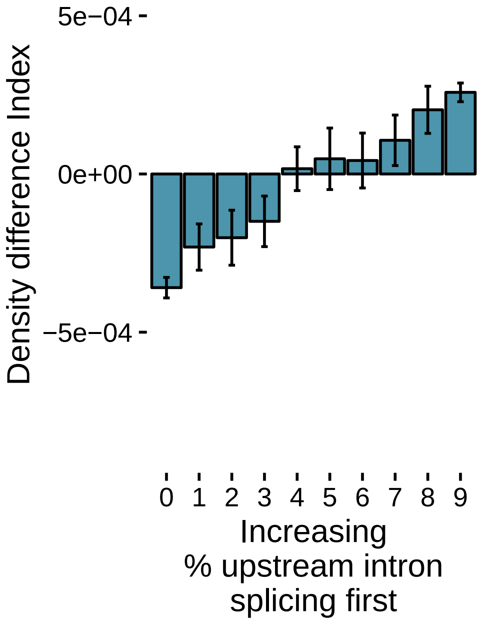

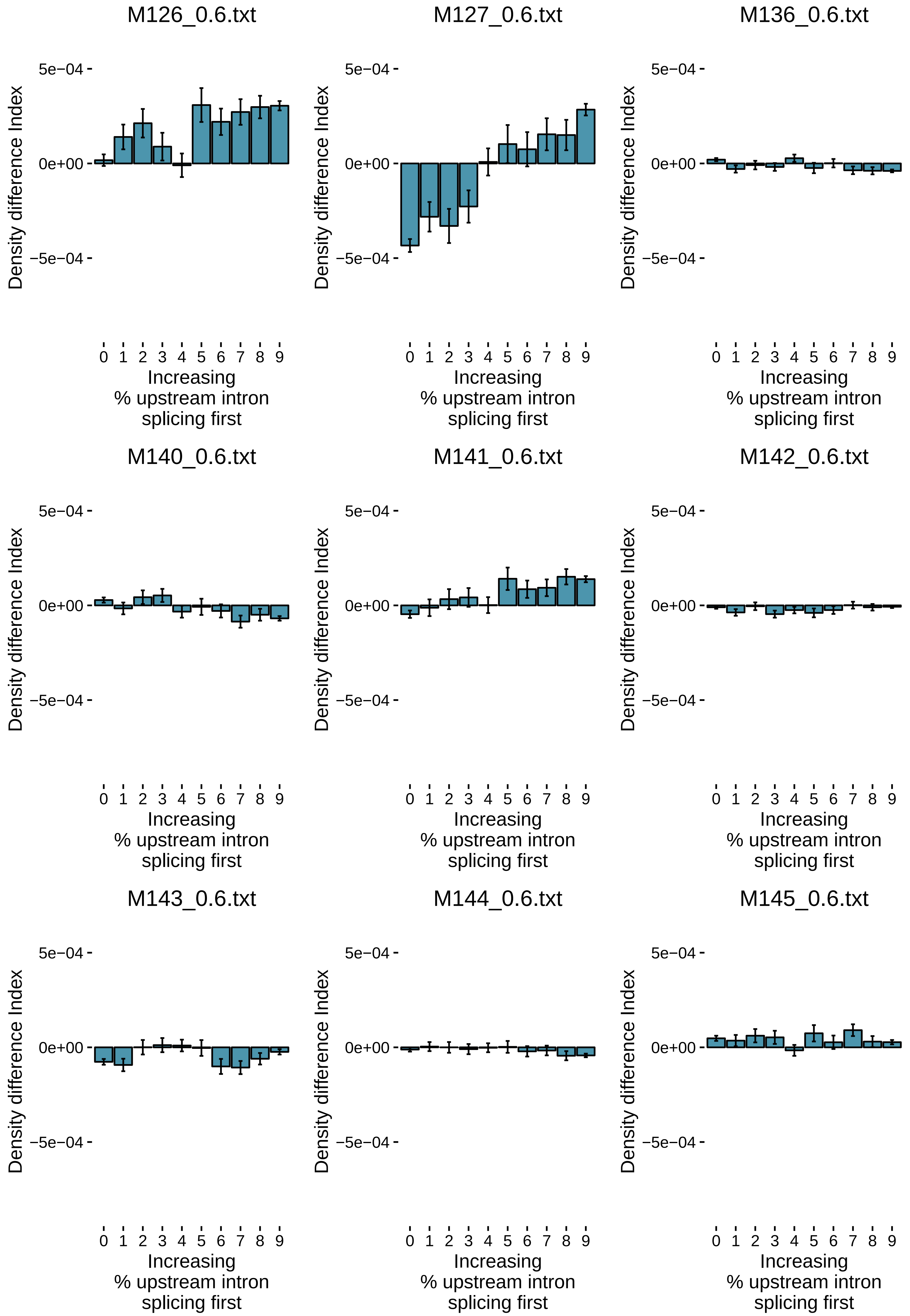
M126_0.6.txt
M127_0.6.txt
M136_0.6.txt
M140_0.6.txt
M141_0.6.txt
M142_0.6.txt
M143_0.6.txt
M144_0.6.txt
M145_0.6.txt
Density difference Index
5e−04
0e+00
−5e−04
0 1 2 3 4 5 6 7 8 9
Increasing
% upstream intron
splicing first

M146_0.6.txt

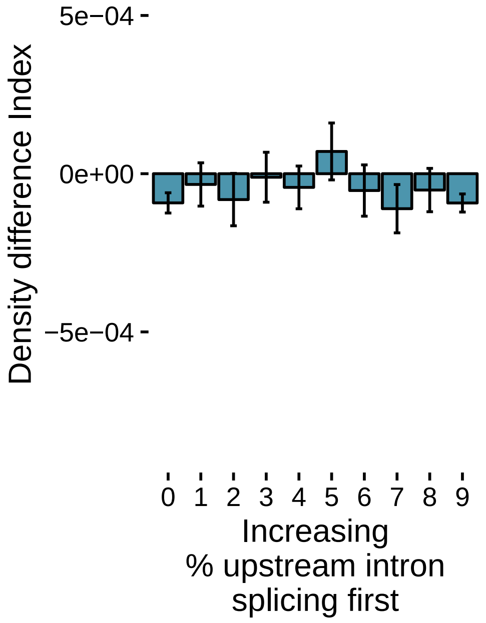

M147_0.6.txt

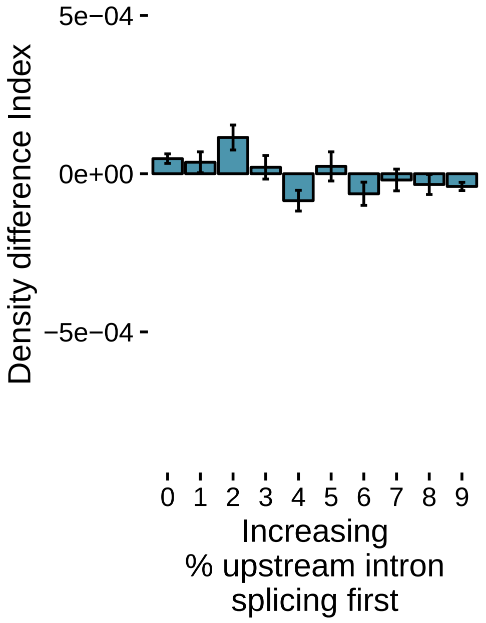

M148_0.6.txt

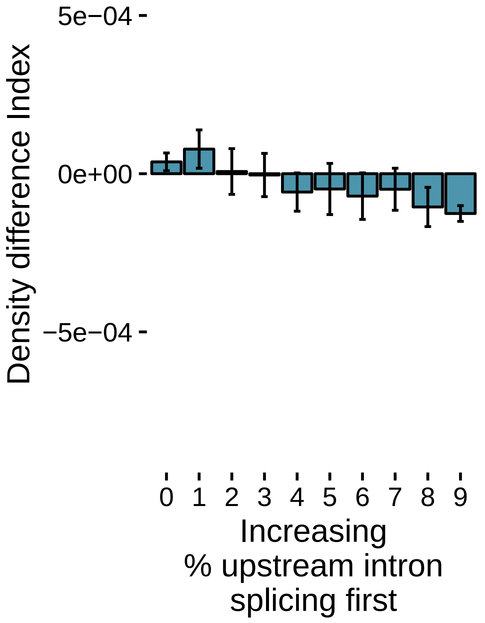

M149_0.6.txt

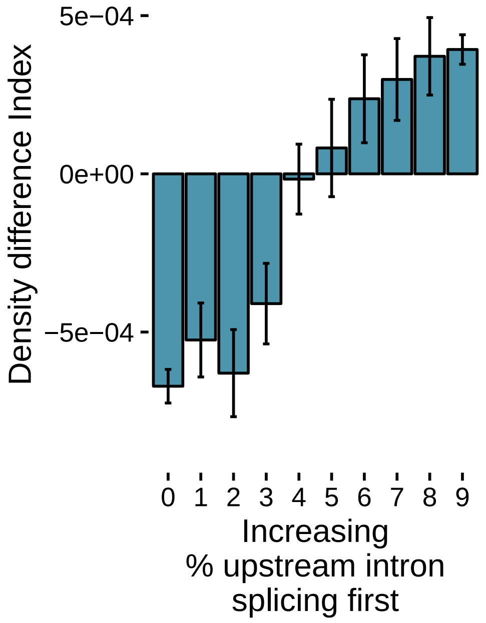

M150_0.6.txt

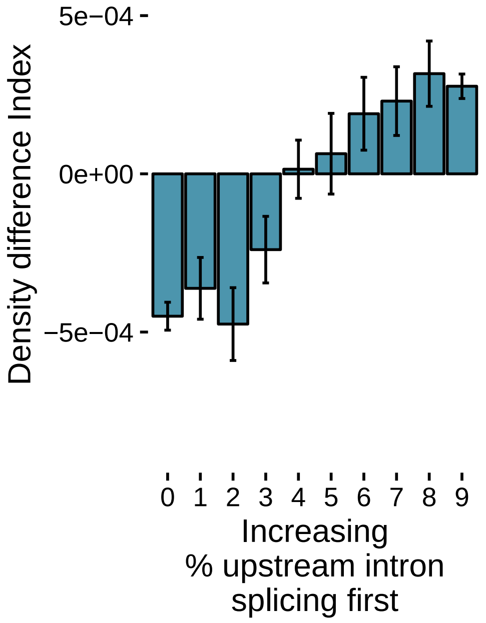

M151_0.6.txt

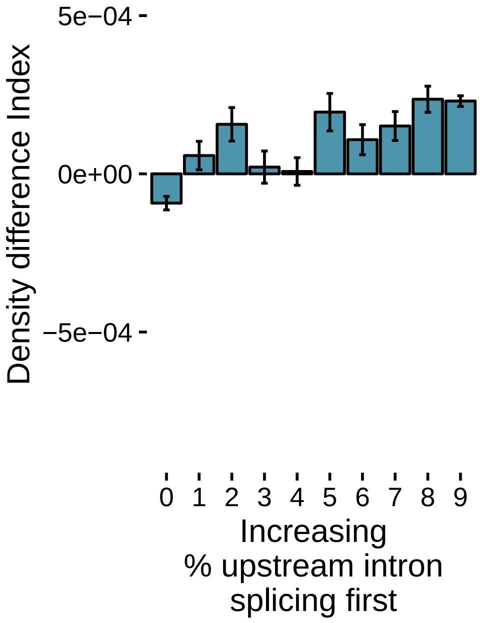

M152_0.6.txt

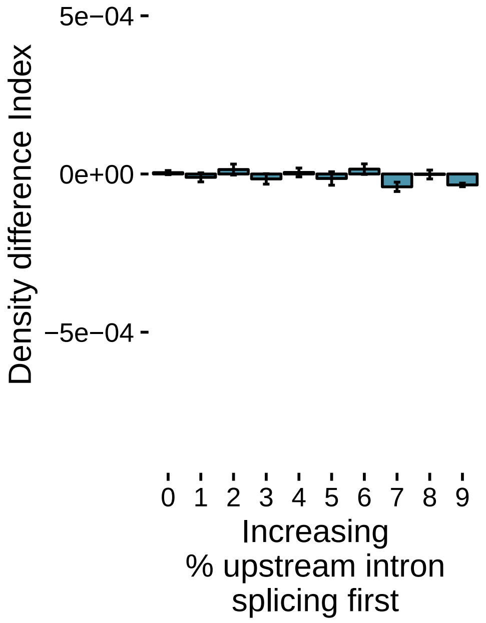

M153_0.6.txt

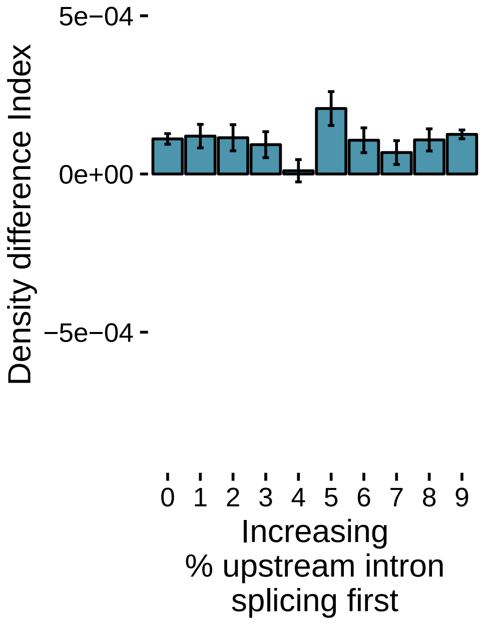

M154_0.6.txt

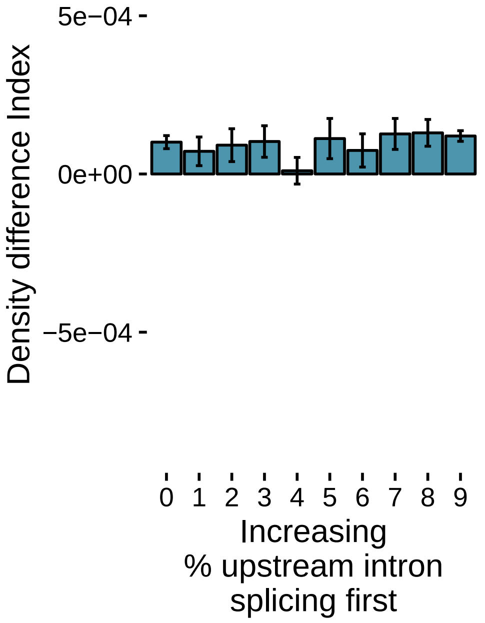

M155_0.6.txt

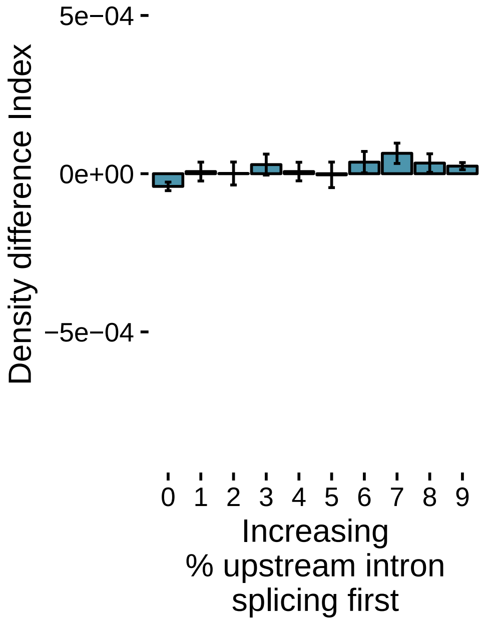

M156_0.6.txt

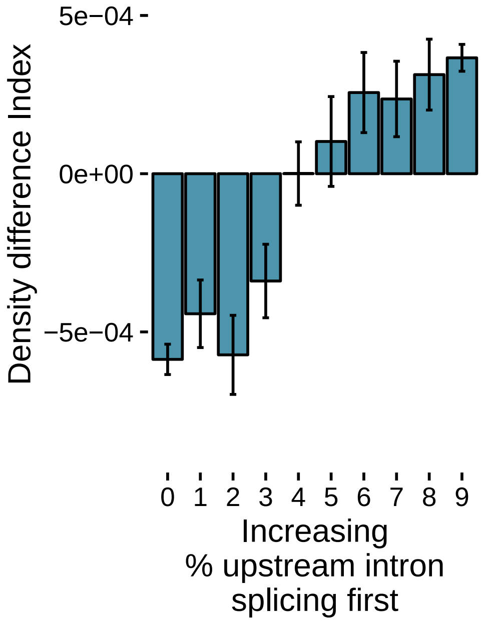

M157_0.6.txt

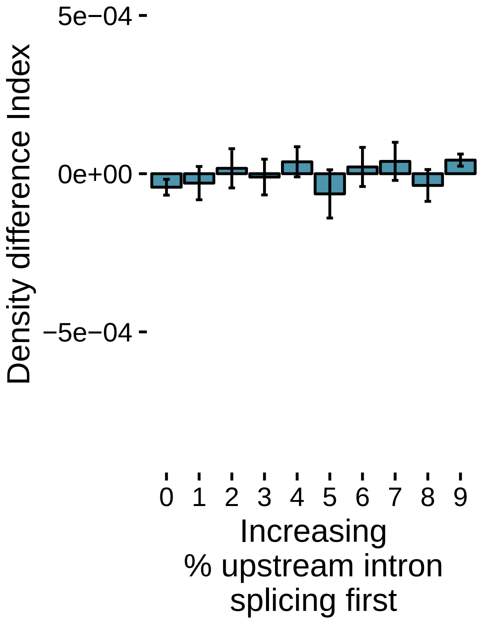

M158_0.6.txt

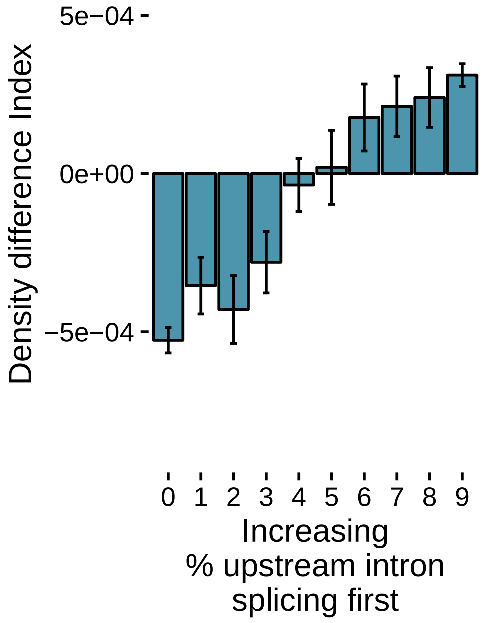

M159_0.6.txt

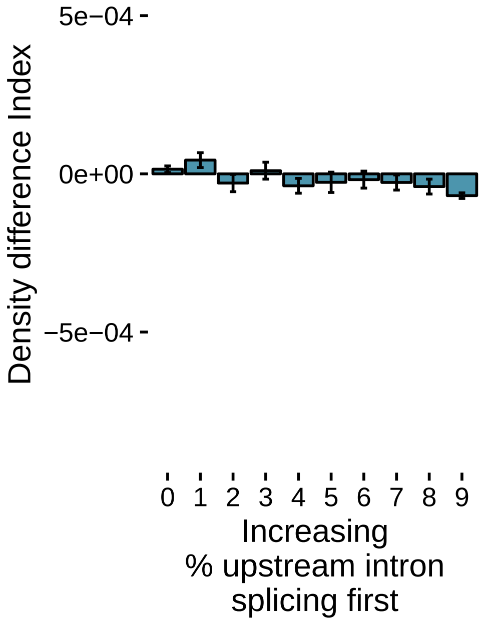

M160_0.6.txt

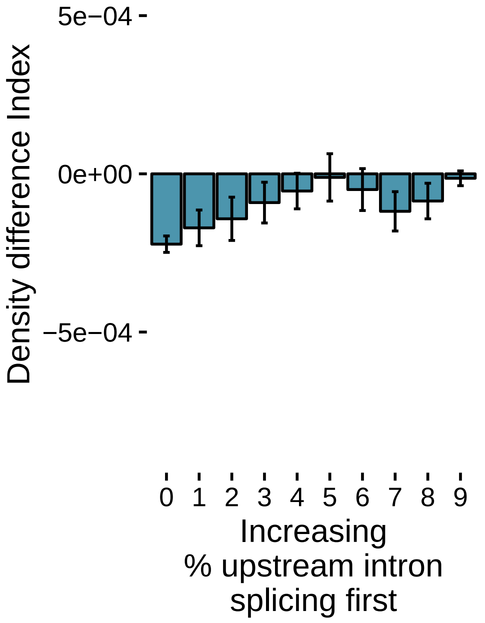

M161_0.6.txt

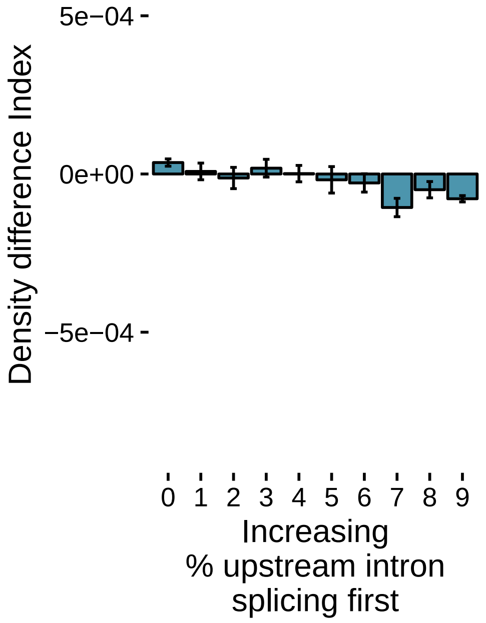

M162_0.6.txt

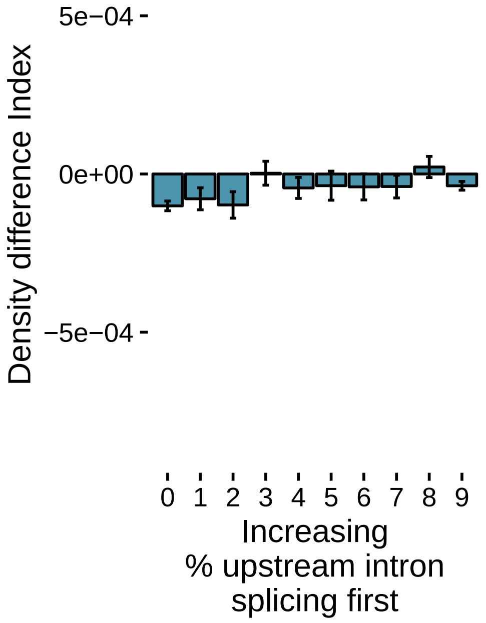

M163_0.6.txt

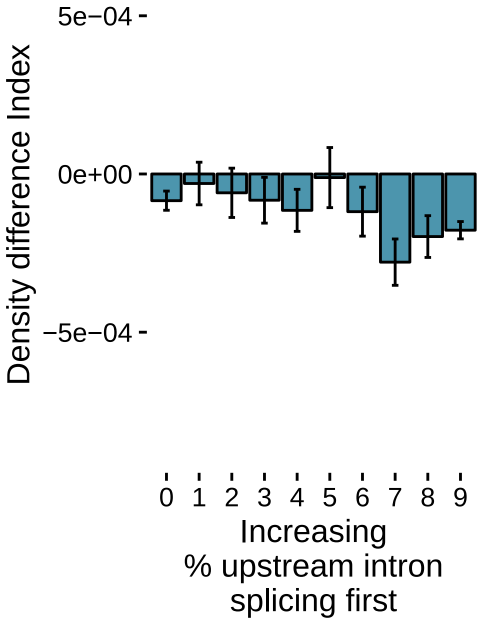

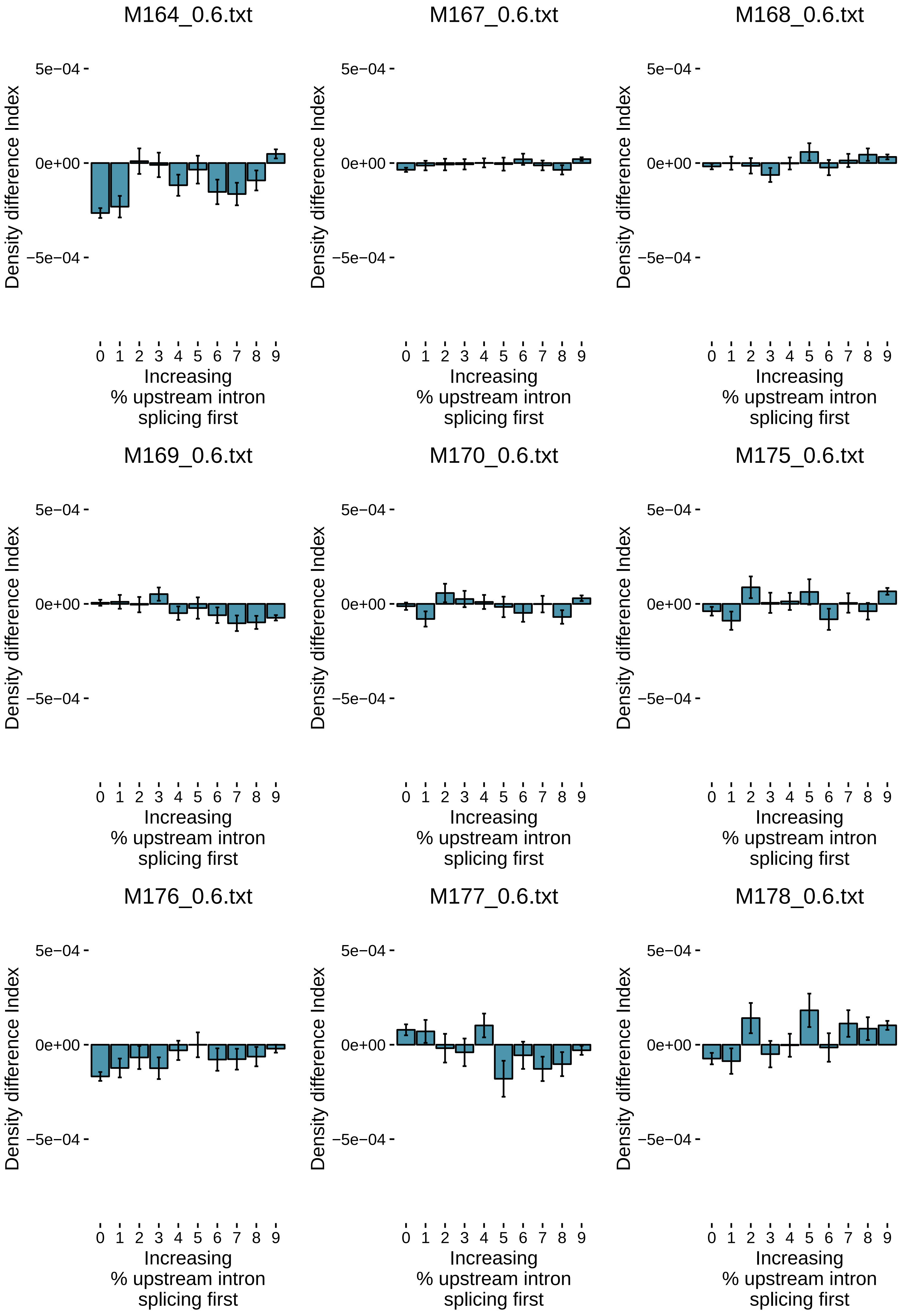
M164_0.6.txt
M167_0.6.txt
M168_0.6.txt
M169_0.6.txt
M170_0.6.txt
M175_0.6.txt
M176_0.6.txt
M177_0.6.txt
M178_0.6.txt
Density difference Index
5e−04
0e+00
−5e−04
0 1 2 3 4 5 6 7 8 9
Increasing
% upstream intron
splicing first

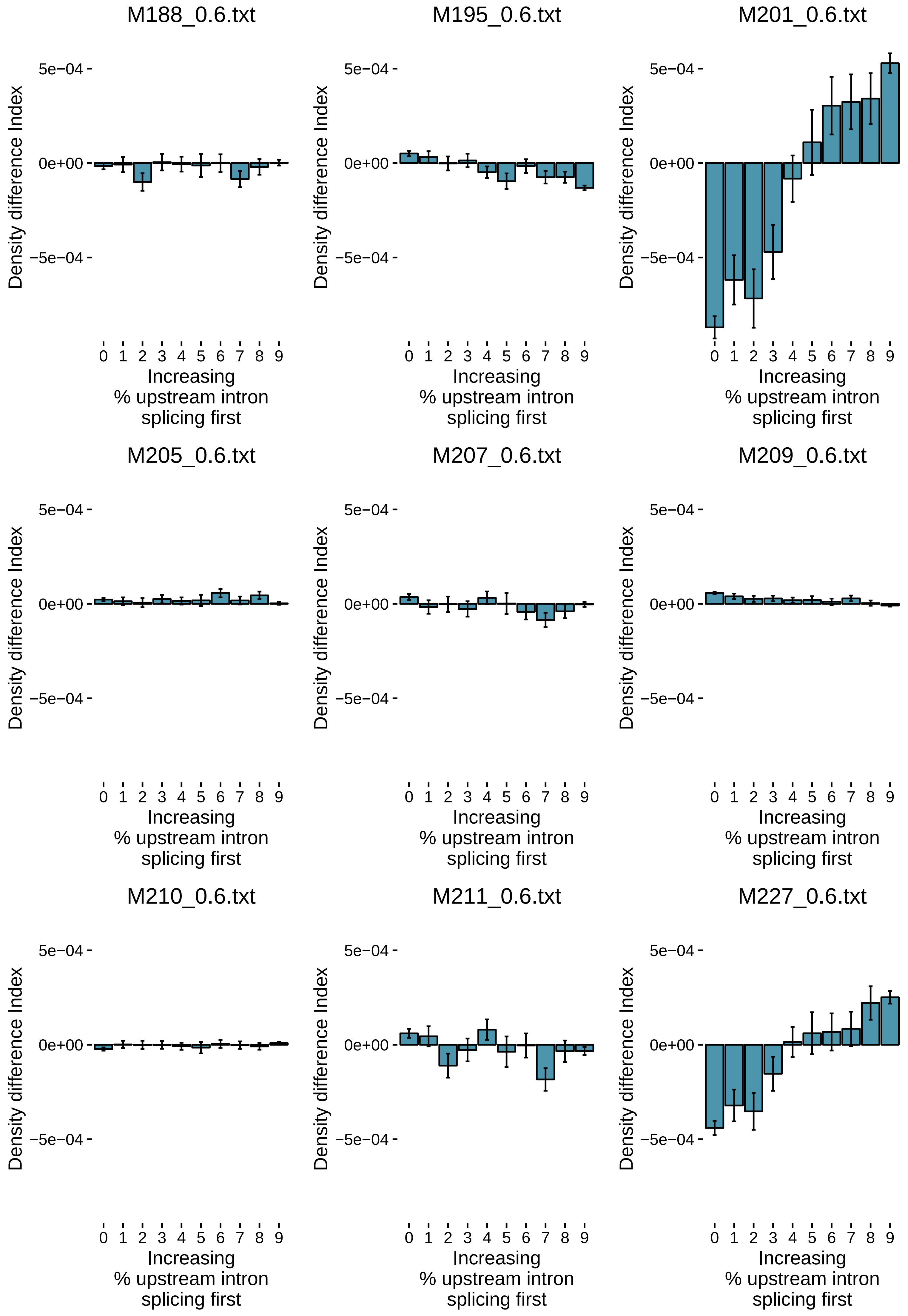
M188_0.6.txt
M195_0.6.txt
M201_0.6.txt
M205_0.6.txt
M207_0.6.txt
M209_0.6.txt
M210_0.6.txt
M211_0.6.txt
M227_0.6.txt
Density difference Index
5e−04
0e+00
−5e−04
0 1 2 3 4 5 6 7 8 9
Increasing
% upstream intron
splicing first

M228_0.6.txt

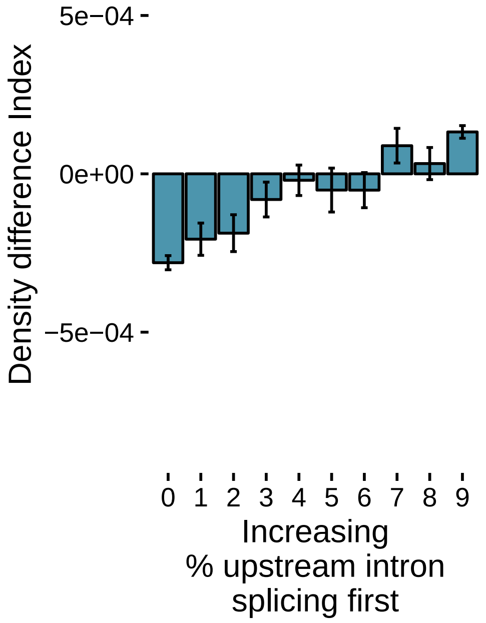

M229_0.6.txt

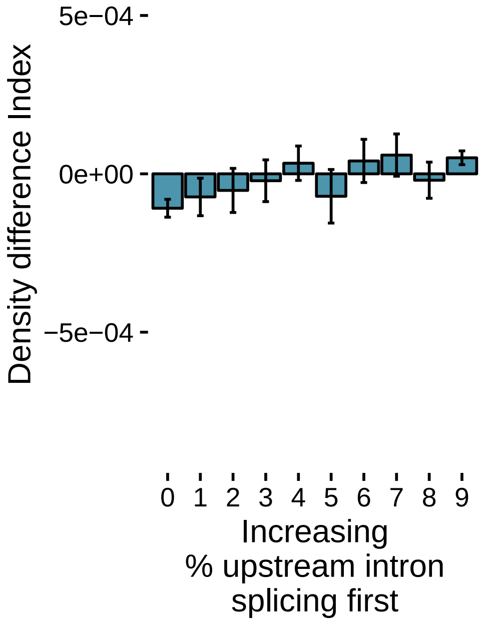

M231_0.6.txt

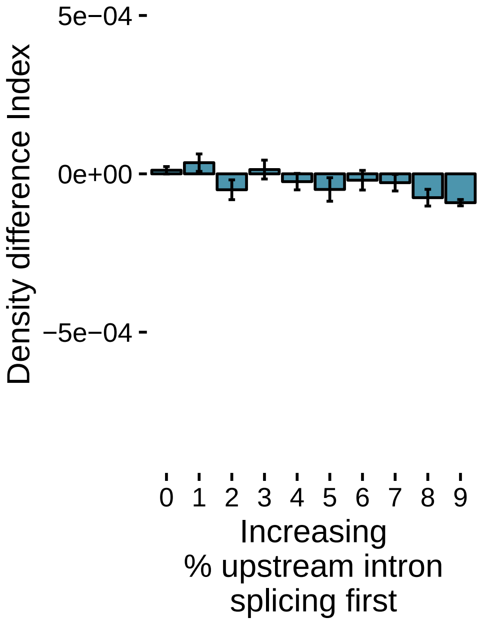

M232_0.6.txt

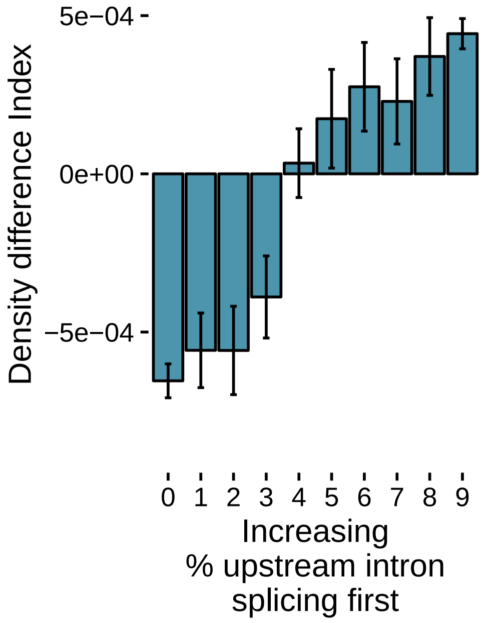

M234_0.6.txt

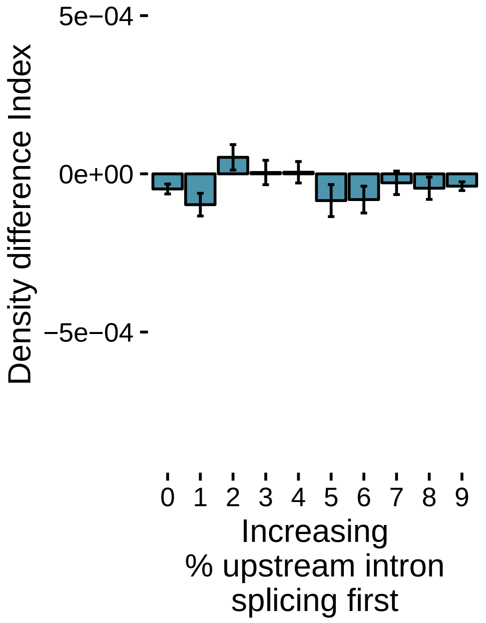

M235_0.6.txt

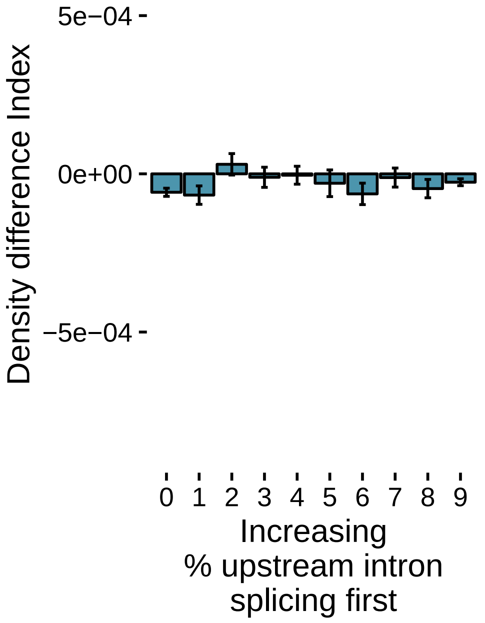

M236_0.6.txt

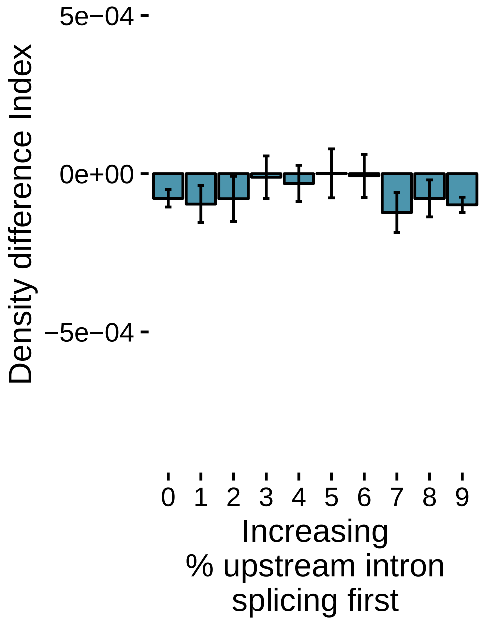

M238_0.6.txt

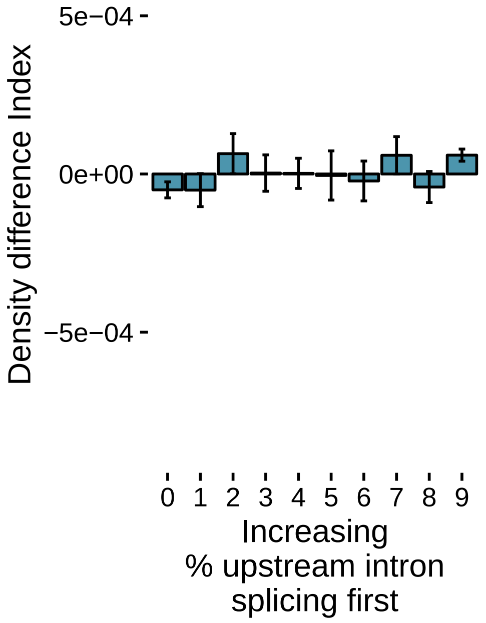

M240_0.6.txt

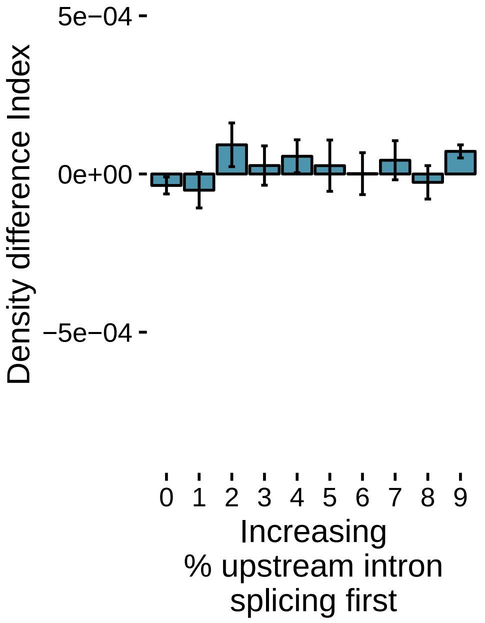

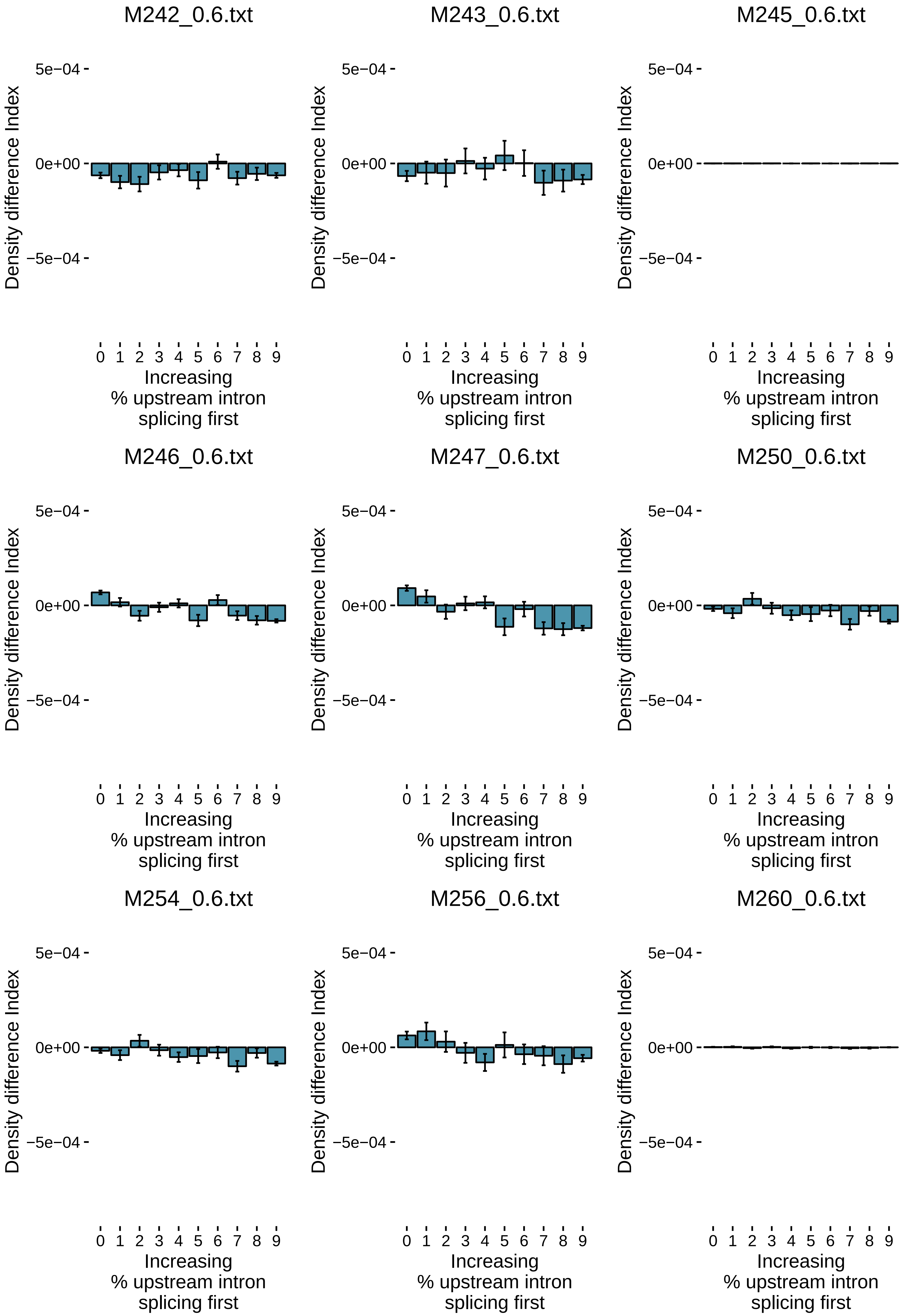
M242_0.6.txt
M243_0.6.txt
M245_0.6.txt
M246_0.6.txt
M247_0.6.txt
M250_0.6.txt
M254_0.6.txt
M256_0.6.txt
M260_0.6.txt
Density difference Index
5e−04
0e+00
−5e−04
0 1 2 3 4 5 6 7 8 9
Increasing
% upstream intron
splicing first

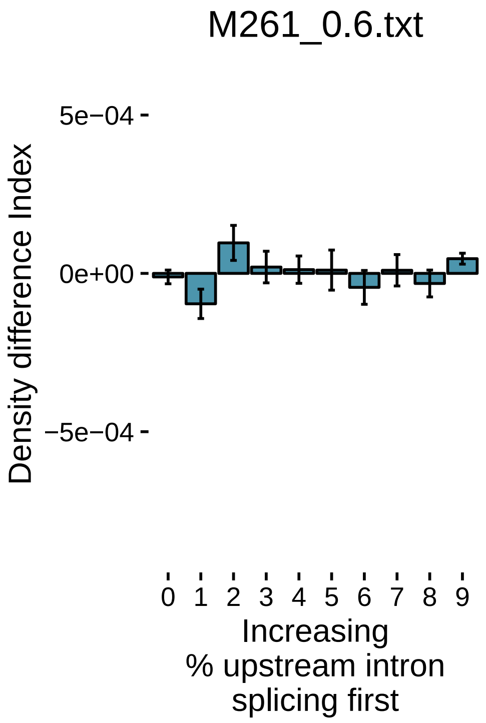
M261_0.6.txt
5e−04
0e+00
−5e−04
Density difference Index
0 1 2 3 4 5 6 7 8 9
Increasing
% upstream intron
splicing first

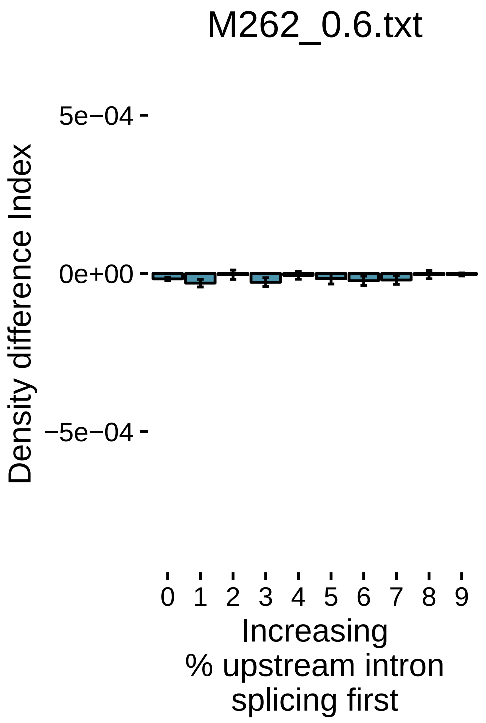
M262_0.6.txt
5e−04
0e+00
−5e−04
Density difference Index
0 1 2 3 4 5 6 7 8 9
Increasing
% upstream intron
splicing first

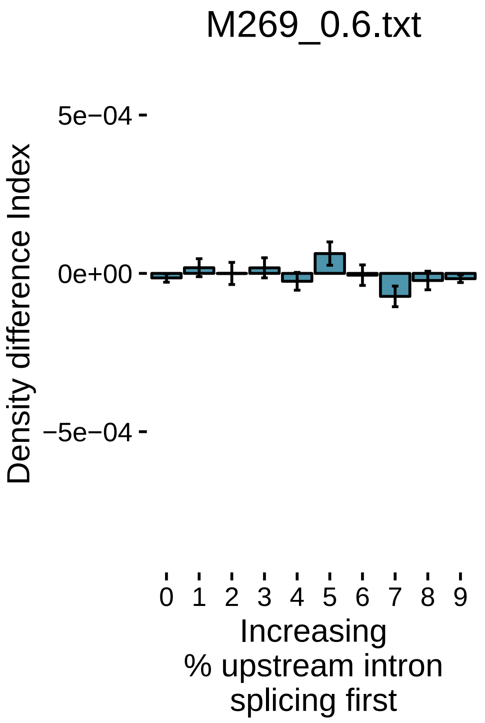
M269_0.6.txt
5e−04
0e+00
−5e−04
Density difference Index
0 1 2 3 4 5 6 7 8 9
Increasing
% upstream intron
splicing first

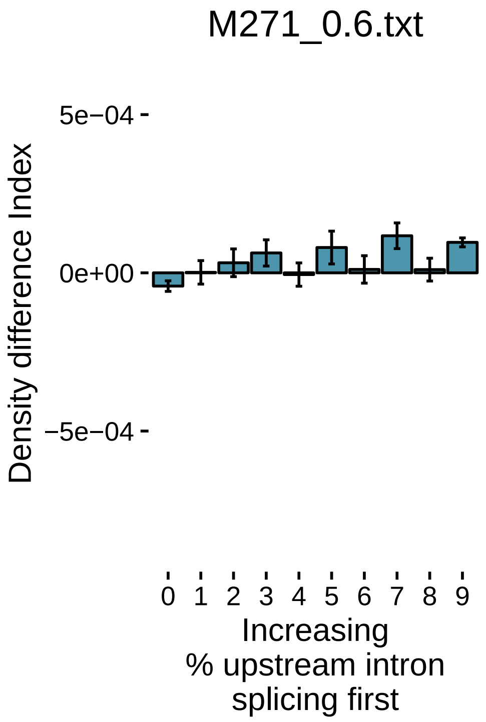
M271_0.6.txt
5e−04
0e+00
−5e−04
Density difference Index
0 1 2 3 4 5 6 7 8 9
Increasing
% upstream intron
splicing first

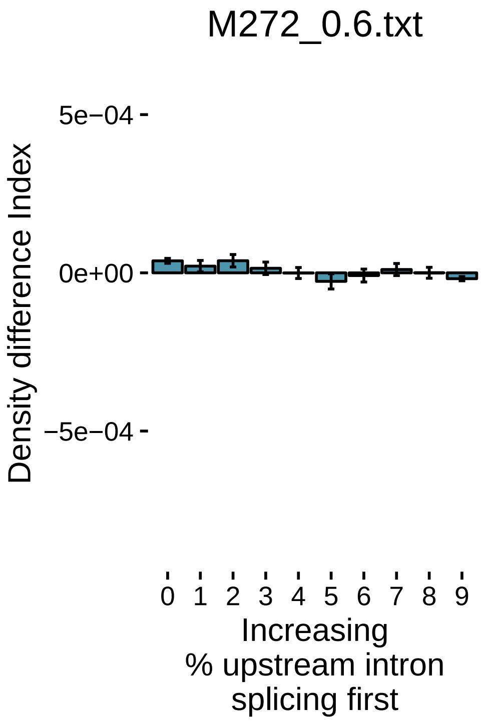
M272_0.6.txt
5e−04
0e+00
−5e−04
Density difference Index
0 1 2 3 4 5 6 7 8 9
Increasing
% upstream intron
splicing first

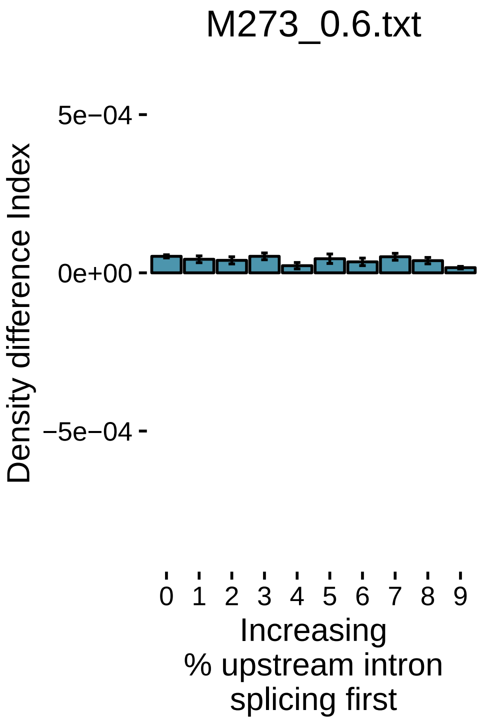
M273_0.6.txt
5e−04
0e+00
−5e−04
Density difference Index
0 1 2 3 4 5 6 7 8 9
Increasing
% upstream intron
splicing first

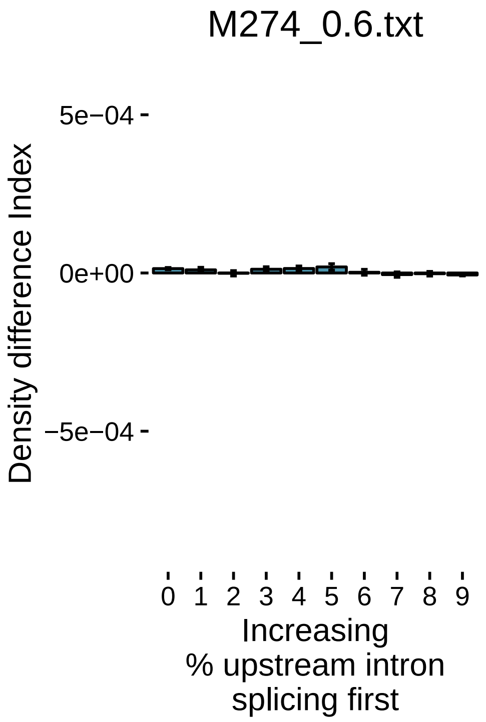
M274_0.6.txt
5e−04
0e+00
−5e−04
Density difference Index
0 1 2 3 4 5 6 7 8 9
Increasing
% upstream intron
splicing first

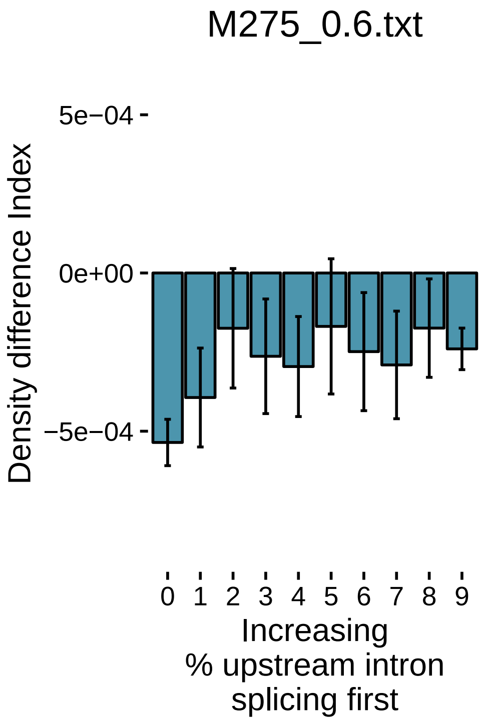
M275_0.6.txt
5e−04
0e+00
−5e−04
Density difference Index
0 1 2 3 4 5 6 7 8 9
Increasing
% upstream intron
splicing first

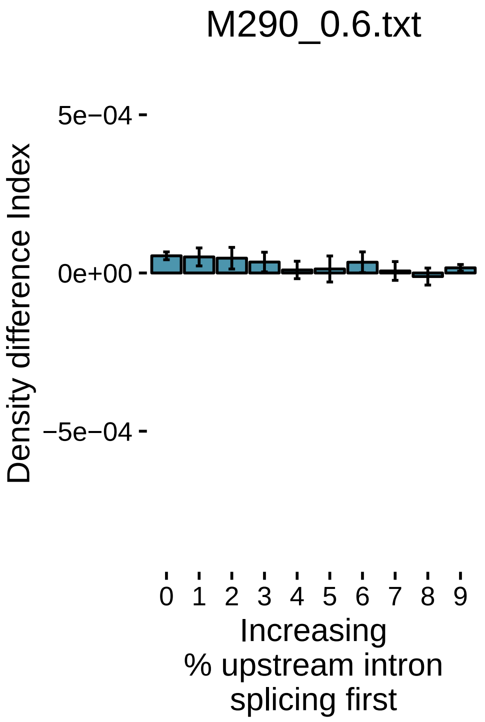
M290_0.6.txt
5e−04
0e+00
−5e−04
Density difference Index
0 1 2 3 4 5 6 7 8 9
Increasing
% upstream intron
splicing first

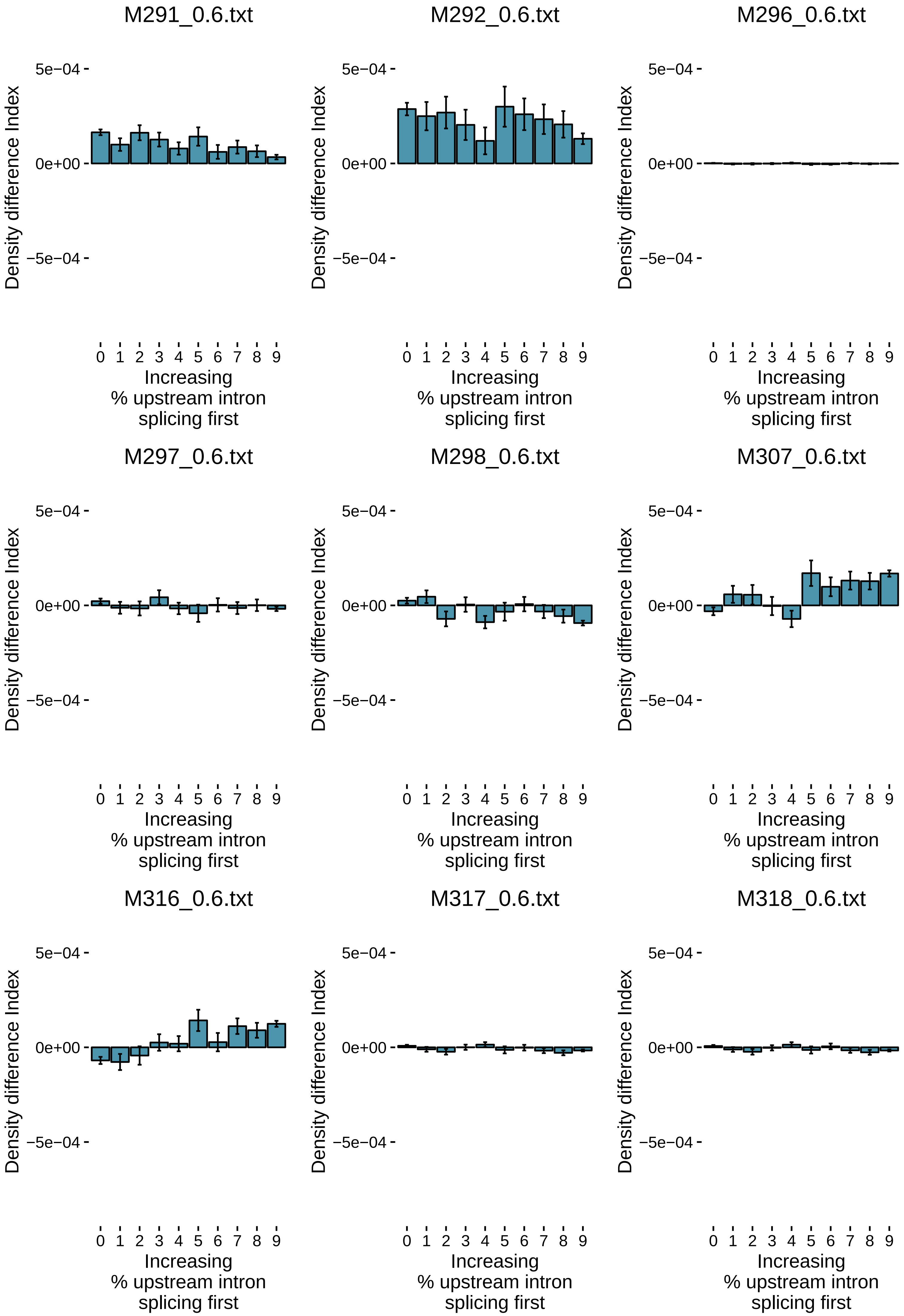
M291_0.6.txt
M292_0.6.txt
M296_0.6.txt
M297_0.6.txt
M298_0.6.txt
M307_0.6.txt
M316_0.6.txt
M317_0.6.txt
M318_0.6.txt
Density difference Index
5e−04
0e+00
−5e−04
0 1 2 3 4 5 6 7 8 9
Increasing
% upstream intron
splicing first

M319_0.6.txt

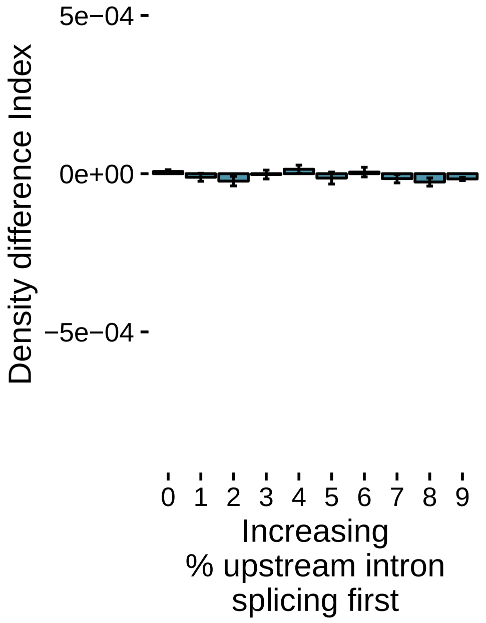

M320_0.6.txt

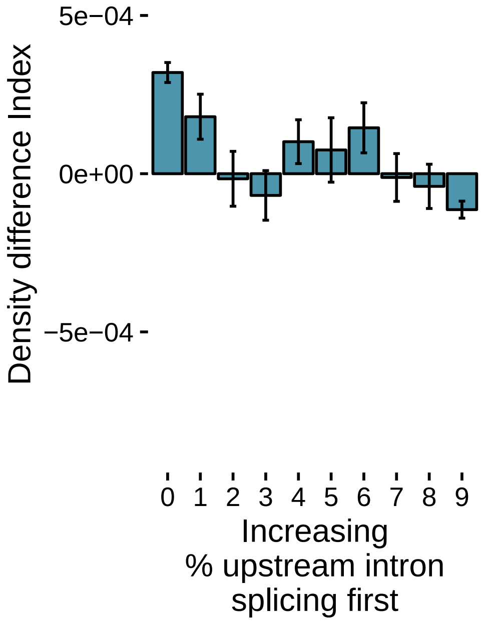

M323_0.6.txt

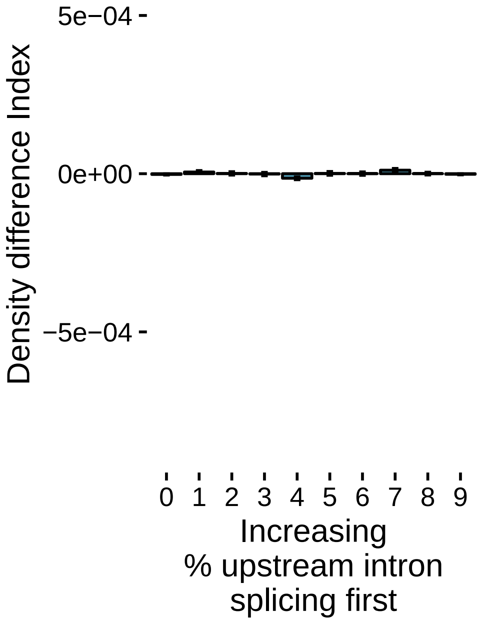

M325_0.6.txt

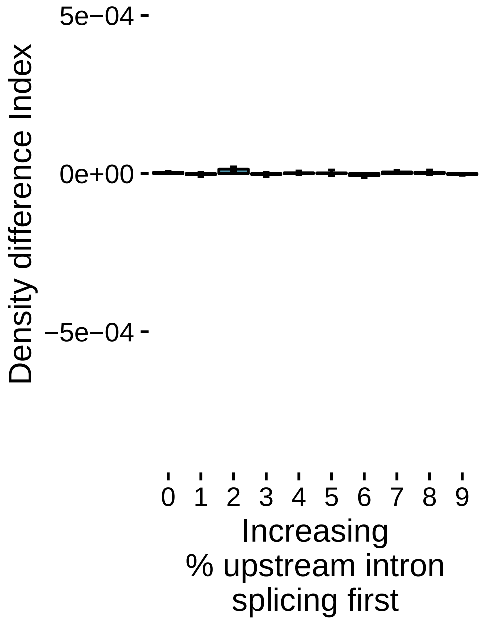

M328_0.6.txt

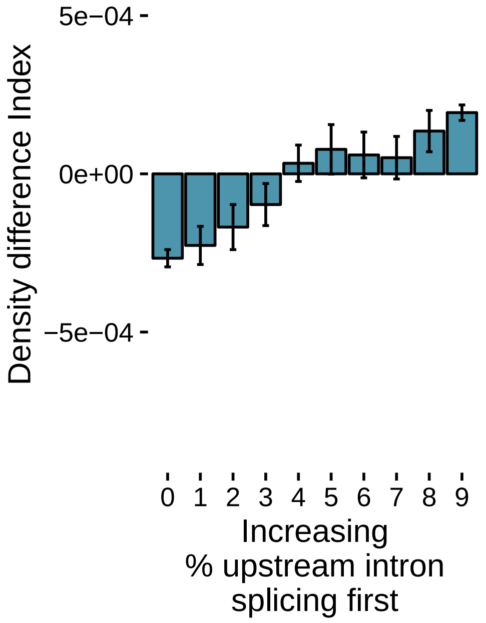

M329_0.6.txt

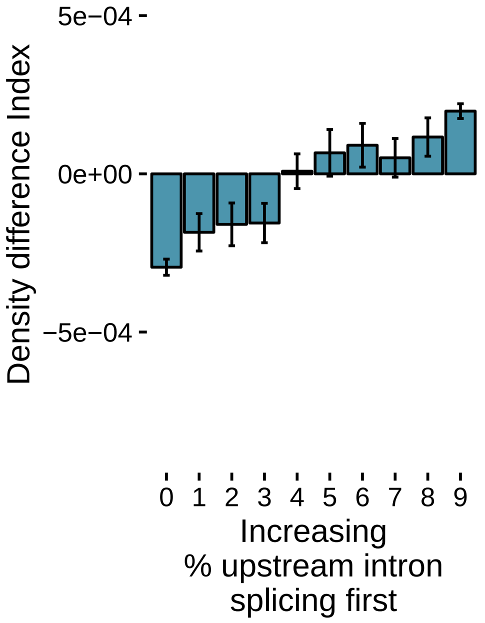

M330_0.6.txt

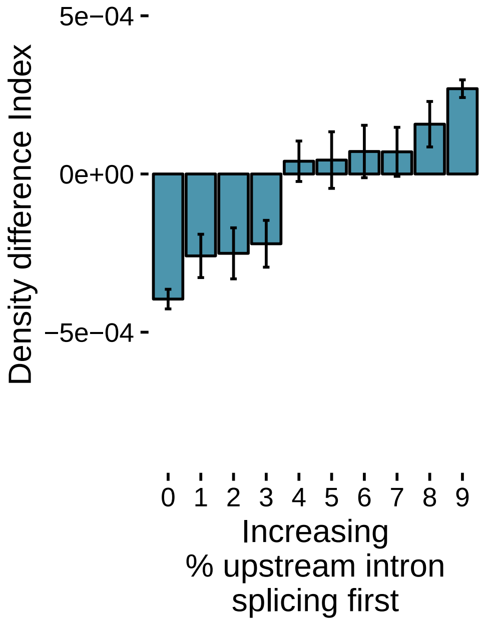

M331_0.6.txt

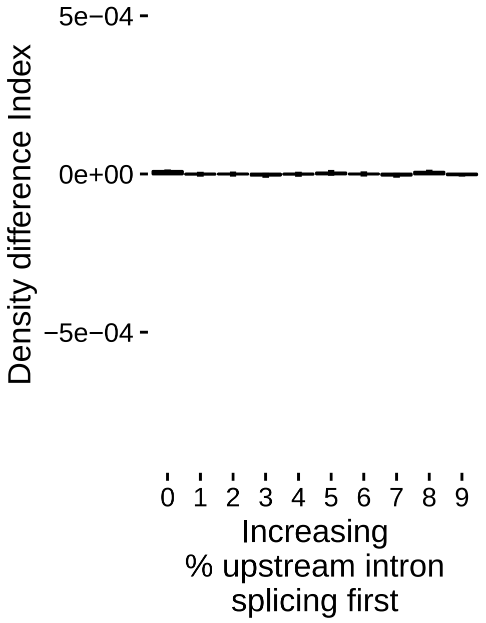

M332_0.6.txt

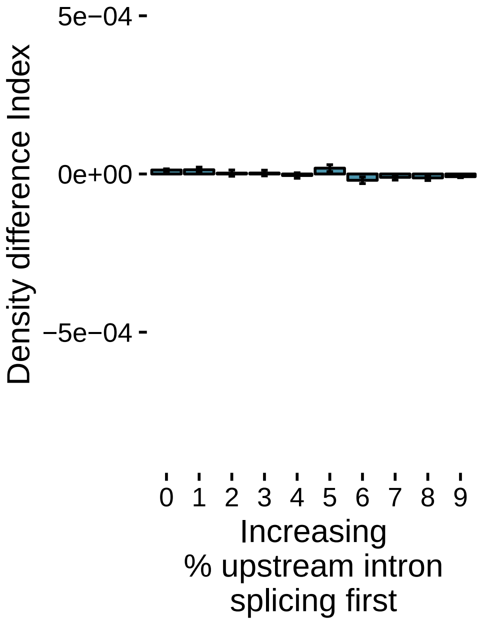

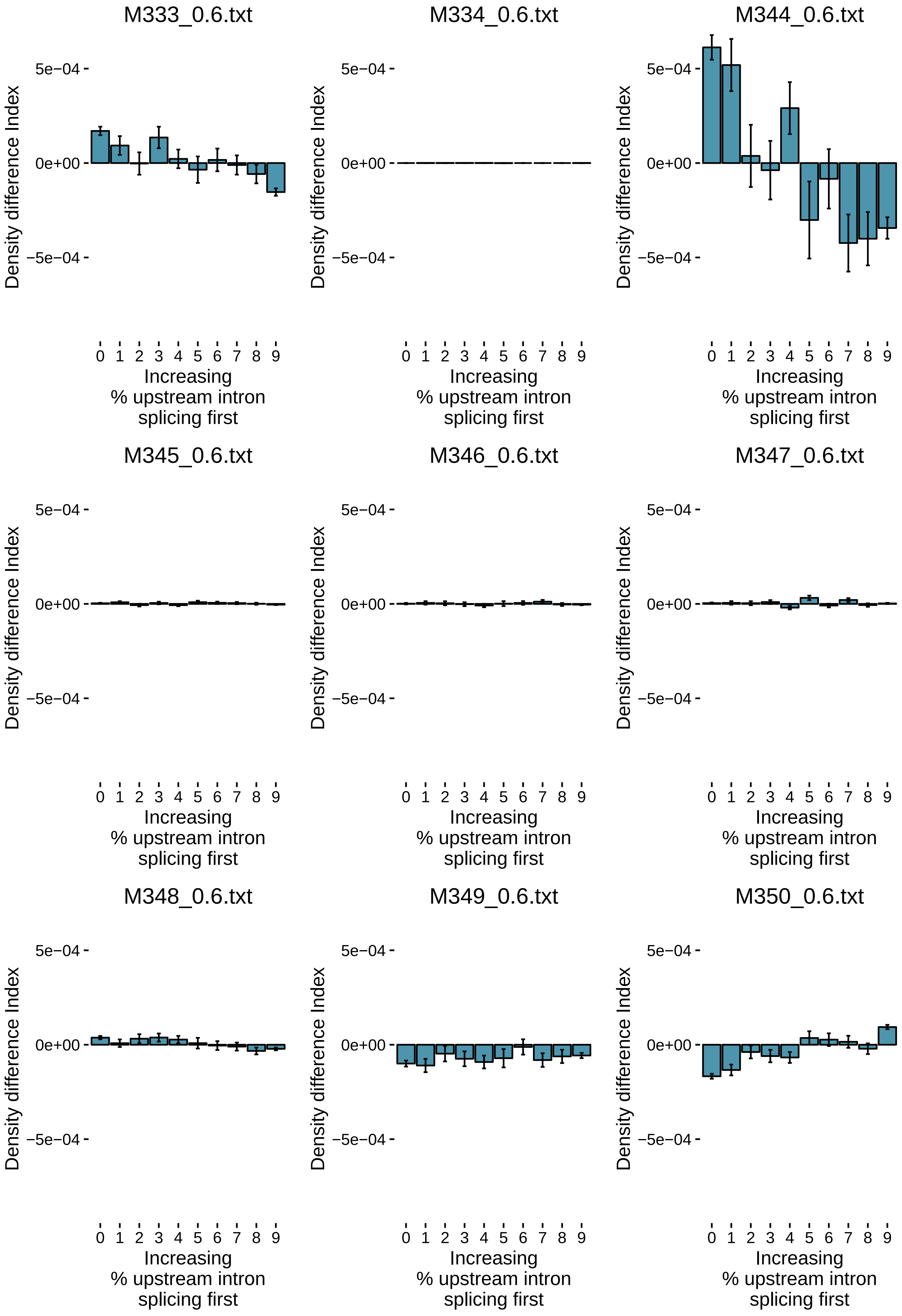
M333_0.6.txt
M334_0.6.txt
M344_0.6.txt
M345_0.6.txt
M346_0.6.txt
M347_0.6.txt
M348_0.6.txt
M349_0.6.txt
M350_0.6.txt
Density difference Index
5e−04
0e+00
−5e−04
0 1 2 3 4 5 6 7 8 9
Increasing
% upstream intron
splicing first

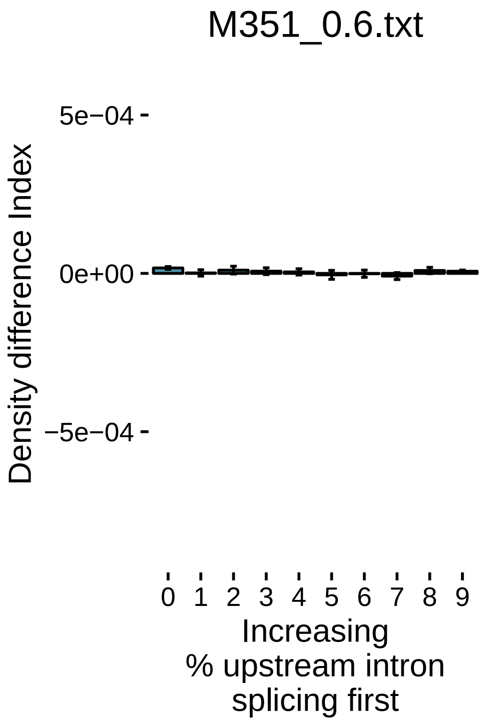
M351_0.6.txt
5e−04
0e+00
−5e−04
Density difference Index
0 1 2 3 4 5 6 7 8 9
Increasing
% upstream intron
splicing first

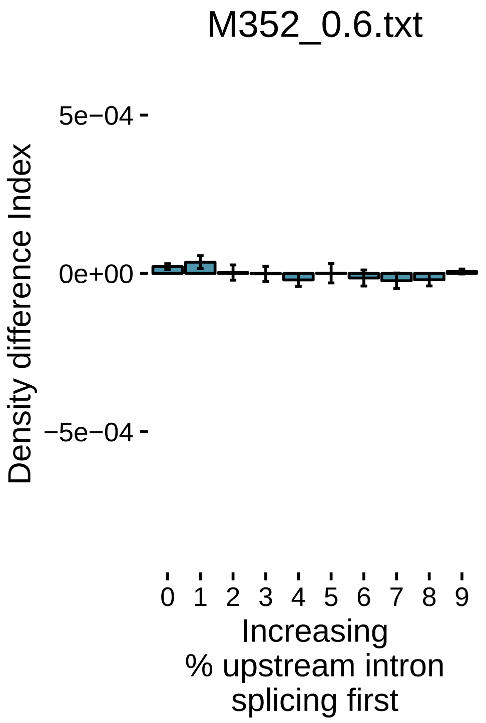
M352_0.6.txt
5e−04
0e+00
−5e−04
Density difference Index
0 1 2 3 4 5 6 7 8 9
Increasing
% upstream intron
splicing first

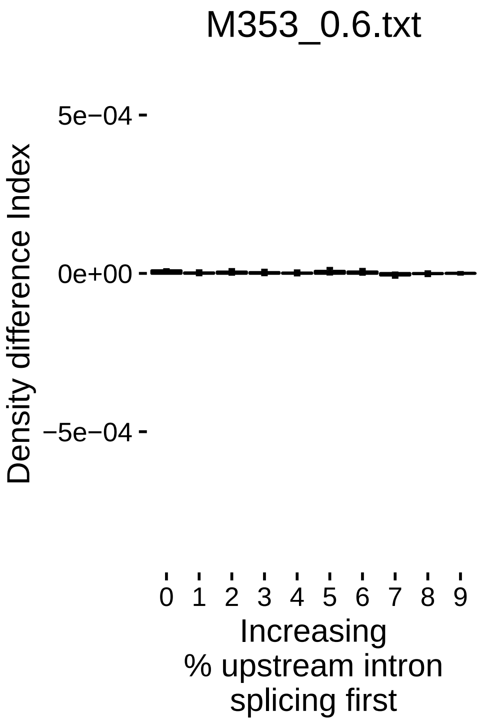
M353_0.6.txt
5e−04
0e+00
−5e−04
Density difference Index
0 1 2 3 4 5 6 7 8 9
Increasing
% upstream intron
splicing first

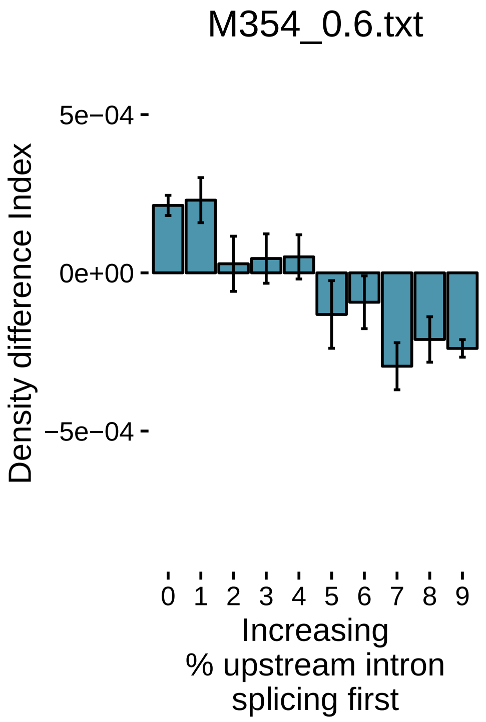
M354_0.6.txt
5e−04
0e+00
−5e−04
Density difference Index
0 1 2 3 4 5 6 7 8 9
Increasing
% upstream intron
splicing first

Supplementary Fig S9. The binding density difference profile of RBPs. All upstream/downstream intronic pairs were analyzed for difference in density of binding sites for 175 human RBPs in RNACompete dataset (http://hugheslab.ccbr.utoronto.ca/Main/HughesData). Pairs were ranked in the ascending order of the upstream intron splicing first and divided based on the range of the original data into ten bins of equal lengths. The density difference index was calculated by subtracting the density of a particular motif in the downstream intron from the density of the same motif in the upstream intron. Clear diagonal trend from bottom left to top right of a plot indicates a candidate activator of intron removal, diagonal trend from top left to bottom right indicates a candidate repressor of intron removal.

Paper	Author	Year	Gene	Introns	Order	Upstream/Total (Fairbrother data)	Agreement?
Order of Intron Removal during Splicing of Endogenous Adenine Phosphoribosyltransfera se and Dihydrofolate Reductase Pre-mRNA	Chasin	1993	APRT	1 vs. 2	upstream first	23/44 (0.52)	yes
Order of Intron Removal during Splicing of Endogenous Adenine Phosphoribosyltransfera se and Dihydrofolate Reductase Pre-mRNA	Chasin	1993	APRT	2 vs. 3	downstream first	6/24 (0.25)	yes
Order of Intron Removal during Splicing of Endogenous Adenine Phosphoribosyltransfera se and Dihydrofolate Reductase Pre-mRNA	Chasin	1993	APRT	3 vs. 4	upstream first	29/42 (0.69)	yes
Order of Intron Removal Influences Multiple Splice Outcomes, Including a Two-Exon Skip, in a COL5A1 Acceptor-Site Mutation That Results	Byers	2002	COL5A1	4 vs. 5	upstream first (in normal cells)	11/16 (0.69)	yes
Order of Intron Removal Influences Multiple Splice Outcomes, Including a Two-Exon Skip, in a COL5A1 Acceptor-Site Mutation That Results	Byers	2002	COL5A1	5 vs. 6	both possible	0/4 (0)	no contradiction
First come, first served revisited: Factors affecting the same alternative splicing event have different effects on the relative rates of intron removal	Kornblihtt	2010	FN1	32 vs. 33	downstream first	no data	no data

Redefinition of exon 7 in the COL1A1 gene of type I collagen by an intron 8 splice-donor-site mutation in a form of osteogenesis imperfecta: influence of intron splice order on outcome of splice-site mutation.	Byers	1999	COL1A1	5 vs. 6	unsure (both rapid)	0/51 (1)	no contradiction
Redefinition of exon 7 in the COL1A1 gene of type I collagen by an intron 8 splice-donor-site mutation in a form of osteogenesis imperfecta: influence of intron splice order on outcome of splice-site mutation.	Byers	1999	COL1A1	6 vs. 7	upstream first	42/51 (0.82)	**yes**
Redefinition of exon 7 in the COL1A1 gene of type I collagen by an intron 8 splice-donor-site mutation in a form of osteogenesis imperfecta: influence of intron splice order on outcome of splice-site mutation.	Byers	1999	COL1A1	7 vs. 8	downstream first (but both pathways observed)	17/53 (0.32)	**yes**
Redefinition of exon 7 in the COL1A1 gene of type I collagen by an intron 8 splice-donor-site mutation in a form of osteogenesis imperfecta: influence of intron splice order on outcome of splice-site mutation.	Byers	1999	COL1A1	8 vs. 9	downstream first	47/60 (0.78)	**no**

Outcome of donor splice site mutations accounting for congenital afibrinogenemia reflects order of intron removal in the fibrinogen alpha gene (FGA)	Neerman-Arbez	2003	FGA	1 vs. 2	downstream first	6/25 (0.24)	**yes**
Outcome of donor splice site mutations accounting for congenital afibrinogenemia reflects order of intron removal in the fibrinogen alpha gene (FGA)	Neerman-Arbez	2003	FGA	2 vs. 3	unsure	no data	no data
Outcome of donor splice site mutations accounting for congenital afibrinogenemia reflects order of intron removal in the fibrinogen alpha gene (FGA)	Neerman-Arbez	2003	FGA	3 vs. 4	upstream first	0/4 (0)	**no**

Supplementary Table 1. Comparison of order of intron removal reported in previous studies versus the conclusions drawn from partially spliced intermediates in RNAseq

C-a_forward	ATTAGCTAGCTGCCTTTCATGTTCTGCAAC
C-a_reverse	TAATGGATCCGGAACACAGTGCTTGGATAGA
C-b_forward	ATTAGCTAGCCGGCAATTGAGGCATTGACT
C-b_reverse	TAATGGATCCCCAGCCAAAAGCTGTAGGAG
C-c_forward	ATTAGCTAGCTGGTGATCGCAGTTCACAGT
C-c_reverse	TAATGGATCCGCTGCTGACTTATTAGGAAGGTC
C-d_forward	ATTAGCTAGCATCCAAGCACTGTGTTCCCC
C-d_reverse	TAATGGATCCTTGGGGACAATGAGACACTG
V-a_forward	ATTAGCTAGCGGCCTTACACCTTCTCAGCA
V-a_reverse	TAATGGATCCCTTTCCATAGGGCCAAGGAC
V-b_forward	ATTAGCTAGCCGGGAGTAGATAGGGCCATA
V-b_reverse	TAATGGATCCTTGATGCGGAAGGTGTCAT
V-c_forward	ATTAGCTAGCGAATTGTGGAGGAACCTGGA
V-c_reverse	TAATGGATCCACCCGTCCATCTTGCCAAG
V-d_forward	ATTAGCTAGCCTTTGAGGAGCGTGGGTC
V-d_reverse	TAATGGATCCCTCGGCATTGAGTAG
B-a_forward	ATTAGCTAGCTTGATCCAAAGGACCTGACC
B-a_reverse	TAATGGATCCCACTGCTACTTCCACCCCAT
B-b_forward	ATTAGCTAGCTGTGACCCCTTATCTGATGCT
B-b_reverse	TAATGGATCCGGACAGGCCGAAATCAGATA
B-c_forward	ATTAGCTAGCCAGTTGTATGGCGTCTGCAC
B-c_reverse	TAATGGATCCGGGAGAAAGGAAGGAAAGGGATA
B-d_forward	ATTAGCTAGCCAGGTTGCTTTCCACGTGG
B-d_reverse	TAATGGATCCAGATTTGCTGCTGAACTTGCT
B-e_forward	ATTAGCTAGCCAGCTCGAAACTGTTTGGTAAA
B-e_reverse	TAATGGATCCTGGAGGTTGCAAAGTGTGAAT
B-f_forward	ATTAGCTAGCTCAGGAAAGCTCAATCAGATACA
B-f_reverse	TAATGGATCCAGCCAGATGAGGCCT
B-g_forward	ATTAGCTAGCCACAAGCTCAGTAGGCTCCA
B-g_reverse	ATTAGCTAGCTAAGCCAAGATCGCGCCA

COL4A5 48_F_Nhe1	ATTAGCTAGCtgcctttcatgttctgcaac
COL4A5_49_R_BamH1	TAATGGATCCagaatcccatccctgaggac
COL4A5_49_F_Nhe1	ATTAGCTAGCtggtgatcgcagttcacagt
COL4A5_50_R_BamH1	TAATGGATCCccagccaaaagctgtaggag
COL4A5_50_F_Nhe1	ATTAGCTAGCatacaagtgcaggggcagaa

COL4A5_51_R_BamH1	TAATGGATCCttggggacaatgagacactg
VPS33B_8_F_Nhe1	ATTAGCTAGCggccttacaccttctcagca
VPS33B_9_R_BamH1	TAATGGATCCatgtccaatctctggccttc
VPS33B_9_F_Nhe1	ATTAGCTAGCgaattgtggaggaacctgga
VPS33B_10_R_BamH1	TAATGGATCCttgatgcggaaggtgtcat
VPS33B_10_F_Nhe1	ATTAGCTAGCgacagcactttgctcccaag
VPS33B_11_R_BamH1	TAATGGATCCctcggcattgagtag
BTK_14_F_Nhe1	ATTAGCTAGCttgatccaaaggacctgacc
BTK_15_R_BamH1	TAATGGATCCtccaggtattccatggcttc
BTK_15_F_Nhe1	ATTAGCTAGCcagttgtatggcgtctgcac
BTK_16_R_BamH1	TAATGGATCCggacaggccgaaatcagata
BTK_16_F_Nhe1	ATTAGCTAGCcagctcgaaactgtttggtaaa
BTK_17_R_BamH1	TAATGGATCCagatttgctgctgaacttgct
BTK_17_F_Nhe1	ATTAGCTAGCcacaagctcagtaggctcca
BTK_18_R_BamH1	TAATGGATCCagccagatgaggcct

SR_c50_spaceF	CAGAAATCAACGTTGAAATCAACGATACCAGAAAATGTGGATCTGATTG
SR_c50_spaceR	GGTATCGTTGATTTCAACGTTGATTTCTGTTACTGGTTCAAATTTATAAC
SR_V9_spaceF	CTGTGATCAACGTAGAAATCAACGGGCTGGGTCAGGGAGAACCT
SR_V9_spaceR	AGCCCGTTGATTTCTACGTTGATCACAGCATGTCCAAGGGCAG

HEATR8-TTC4	GAAGGTCTAGGTGAGCTTTTCCTGG
HEATR8-TTC4	TGGGCATACTCTGGGTACAGAAAGA
RAN	TTGAGAAGAAGTATGTAGGTATGTGCTGGA
RAN	CCCAAGGTGGCTGAAACGGA
DHX9	CTGGCCTTTGGTGTGTACCC
DHX9	GTCTTGGCTACTAAAAGGACAATTAACAGA
PPM1J	AAAGGACCTGGTAGAGATACTTCAGGAC
PPM1J	TCACTTCCTTCTGTGAAGACCAGCA
RABEP2	GTGCTGGAGGGTGAGTGTGT
RABEP2	CACAGGGAGGCTGGGAAGTC
CD19	TTCTCCAACGGTAACTTGGGGC
CD19	TAAGACTCAGCTGTGGCAGAAGAGA

NETO2	TACCTAGGAGGTATTTTAAATCCCATTCCA
NETO2	AACTGACAATCTGGAAGGAATACATGAAAG
CTU2	GACGCCGCTGGTCTGTGTTT
CTU2	GTGGCACTGTCTGCAAGCAG
SUPT5H	GCTCACCACGGTGTACGGGC
SUPT5H	CCGTGAGCCCCTGGGGA
RRAS	CGGCTGGACAGTGAGGGC
RRAS	GTGTCCAGGACTGCAGAGACAG

LS2_stitch_F	TTCGTCCTCAACGCTCTTCCGATCTGAAGGTCTAGGTGAGCTTTT
LS2_stitch_R	CATGCTCCTCCGTCAGAGCTTGGTTGGGCATACTCTGGGTA
LS3_stitch_F	TTCGTCCTCAACGCTCTTCCGATCTAAAGGACCTGGTAGAGATAC
LS3_stitch_R	CATGCTCCTCCGTCAGAGCTTGGTTCACTTCCTTCTGTGAAGA
LS4_stitch_F	TTCGTCCTCAACGCTCTTCCGATCTCTGGCCTTTGGTGTG
LS4_stitch_R	CATGCTCCTCCGTCAGAGCTTGGTGTCTTGGCTACTAAAAGGACAATTAACAGA
LS7_stitch_F	TTCGTCCTCAACGCTCTTCCGATCTTTGAGAAGAAGTATGTAGGT
LS7_stitch_R	CATGCTCCTCCGTCAGAGCTTGGTCCCAAGGTGGCTGAAA
LS9_Stitch_F	TTCGTCCTCAACGCTCTTCCGATCTGTGCTGGAGGGTGA
LS9_Stitch_R	CATGCTCCTCCGTCAGAGCTTGGTCACAGGGAGGCTGG
LS10_Stitch_F	TTCGTCCTCAACGCTCTTCCGATCTTTCTCCAACGGTAACTTGG
LS10_Stitch_R	CATGCTCCTCCGTCAGAGCTTGGTTAAGACTCAGCTGTGGCAGA
LS11_Stitch_F	TTCGTCCTCAACGCTCTTCCGATCTTACCTAGGAGGTATTTTAAA
LS11_Stitch_R	CATGCTCCTCCGTCAGAGCTTGGTAACTGACAATCTGGAAGGAATACATGAAAG
LS12_stitch_F	*TTCGTCCTCAACGCTCTTCCGATCTGACGCCGCTGGTCT*
LS12_stitch_R	CATGCTCCTCCGTCAGAGCTTGGTGTGGCACTGTCTGCA
LS14_stitch_F	TTCGTCCTCAACGCTCTTCCGATCTGCTCACCACGGTGTA
LS14_stitch_R	CTCCTCCGTCAGAGCTTGGTCCGTGAGCCCCTGGGGA
LS16_stitch_F	TTCGTCCTCAACGCTCTTCCGATCTCGGCTGGACAGTGA
LS16_stitch_R	CATGCTCCTCCGTCAGAGCTTGGTGTGTCCAGGACTGCAGAGA

MCOLN2_F	TTCGTCCTCAACGCTCTTCCGATCTAATTTTATCGgtaaa
MCOLN2_R	catgctcctCCGTCAGAGCTTGGTCTGTAAGAGCctgaa
EBNA1BP2_F	TTCGTCCTCAACGCTCTTCCGATCTAGACAGAGAGgtagg
EBNA1BP2_R	catgctcctCCGTCAGAGCTTGGTCATCCTGCAActgca
MFSD12_F	TTCGTCCTCAACGCTCTTCCGATCTCACACCCGTGgtcag
MFSD12_R	catgctcctCCGTCAGAGCTTGGTTCTCCGTGATctaga
SEMA4C_F	TTCGTCCTCAACGCTCTTCCGATCTCCTGCCCAAGgtgag

SEMA4C_R	catgctcctCCGTCAGAGCTTGGTCGATGAACTTctgcg
UBA5_F	TTCGTCCTCAACGCTCTTCCGATCTGAACAACCAGgtggg
UBA5_R	catgctcctCCGTCAGAGCTTGGTAGCACTCGGTctggg

Supplementary Table 2. List of all primer sequences used in study.

matrix_id	RBP(s)
M001_0.6.txt	A1CF
M002_0.6.txt	ANKHD1,ANKRD17,ENSG00000249536
M004_0.6.txt	BRUNOL4,CELF3
M012_0.6.txt	CPEB3,CPEB2
M013_0.6.txt	DAZAP1
M016_0.6.txt	FMR1
M017_0.6.txt	RBFOX2,RBFOX3
M019_0.6.txt	SRSF12,Fusip1,BX511012.1
M020_0.6.txt	FXR2
M021_0.6.txt	G3BP2
M022_0.6.txt	HNRNPA1,HNRNPA3,ENSG00000215492,ENSG00000231942
M023_0.6.txt	HNRNPA1L2,HNRNPA3,ENSG00000215492,ENSG00000231942
M024_0.6.txt	HNRNPA2B1,HNRNPA3,ENSG00000215492
M025_0.6.txt	HNRNPC
M026_0.6.txt	hnRNPK
M027_0.6.txt	HNRNPL,ENSG00000215042
M031_0.6.txt	ELAVL1,ELAVL3
M032_0.6.txt	IGF2BP2
M033_0.6.txt	KHDRBS3
M035_0.6.txt	LIN28A,LIN28B
M036_0.6.txt	MATR3
M037_0.6.txt	MBNL3,MBNL2,MBNL1
M040_0.6.txt	MSI1,MSI2
M042_0.6.txt	PABPC4,PABPC1L,ENSG00000250177
M043_0.6.txt	PCBP4,PCBP3,PCBP2
M044_0.6.txt	PPRC1
M046_0.6.txt	QKI
M047_0.6.txt	RBM28
M048_0.6.txt	CIRBP,RBM3
M049_0.6.txt	RBM38
M050_0.6.txt	RBM4B,RBM4,RBM4
M051_0.6.txt	RBM41
M052_0.6.txt	RBM46,RBM47
M053_0.6.txt	RBM5
M054_0.6.txt	RBM8A
M055_0.6.txt	RBMS2,RBMS3,ENSG00000213250
M056_0.6.txt	SRSF3
M061_0.6.txt	SAMD4A,SAMD4B
M062_0.6.txt	SART3
M065_0.6.txt	SRSF9
M068_0.6.txt	SNRNP70

M069_0.6.txt	SNRPA,SNRPB2
M070_0.6.txt	SRSF2,ENSG00000180771
M071_0.6.txt	SRSF7
M072_0.6.txt	SRSF9
M073_0.6.txt	STAR-PAP
M074_0.6.txt	TARDBP
M075_0.6.txt	TIA1
M077_0.6.txt	U2AF2
M079_0.6.txt	CELF3
M081_0.6.txt	CSDA,YB-1
M082_0.6.txt	YBX2,CSDA
M083_0.6.txt	ZC3H10
M085_0.6.txt	ZCRB1
M086_0.6.txt	SRSF12,Fusip1,BX511012.1
M087_0.6.txt	SRSF12,Fusip1,BX511012.1
M088_0.6.txt	SRSF12,Fusip1,BX511012.1
M089_0.6.txt	HNRNPL,ENSG00000215042
M102_0.6.txt	SRSF1
M103_0.6.txt	SRSF1
M104_0.6.txt	SRSF1
M105_0.6.txt	SRSF1
M106_0.6.txt	SRSF1
M108_0.6.txt	ELAVL1,ELAVL3
M109_0.6.txt	RBM4B,RBM4,RBM4
M111_0.6.txt	CSDA,YB-1
M112_0.6.txt	ELAVL1,ELAVL3
M118_0.6.txt	RBFOX2,RBFOX3
M120_0.6.txt	CPEB3
M121_0.6.txt	SRSF3
M122_0.6.txt	MEX3C,MEX3D,MEX3B
M124_0.6.txt	ELAVL3
M126_0.6.txt	SRSF4,SRSF6
M127_0.6.txt	ELAVL1,ELAVL3
M136_0.6.txt	SNRPB2
M140_0.6.txt	ENOX1,ENOX2
M141_0.6.txt	ESRP2,ESRP1
M142_0.6.txt	RBM42
M143_0.6.txt	RBMS2,RBMS1,ENSG00000213250
M144_0.6.txt	PABPC1L,PABPC3
M145_0.6.txt	RBM5
M146_0.6.txt	PABPC1,PABPC1L,ENSG00000250177
M147_0.6.txt	CNOT4

M148_0.6.txt	PABPN1,PABPN1L
M149_0.6.txt	CPEB3,CPEB4
M150_0.6.txt	RALY
M151_0.6.txt	HNRNPH2,HNRNPH1,HNRNPF
M152_0.6.txt	FXR1
M153_0.6.txt	LIN28A,LIN28B
M154_0.6.txt	SRSF1
M155_0.6.txt	ZNF638
M156_0.6.txt	TIA1
M157_0.6.txt	CELF3,BRUNOL5
M158_0.6.txt	HNRNPCL1
M159_0.6.txt	A2BP1,RBFOX2,RBFOX3
M160_0.6.txt	KHDRBS1
M161_0.6.txt	RBM6
M162_0.6.txt	PABPC5
M163_0.6.txt	IGF2BP3
M164_0.6.txt	RBMS2,RBMS3,ENSG00000213250
M167_0.6.txt	MSI1,MSI2
M168_0.6.txt	SFPQ,PSPC1
M169_0.6.txt	hnRNPLL
M170_0.6.txt	RBM38
M175_0.6.txt	RBM24,RBM38
M176_0.6.txt	KHDRBS2
M177_0.6.txt	PCBP4,PCBP1,PCBP3
M178_0.6.txt	BRUNOL6,CELF3
M188_0.6.txt	PCBP4,PCBP3
M195_0.6.txt	SF3B4
M201_0.6.txt	SF3B4
M205_0.6.txt	SF3B4
M207_0.6.txt	PCBP4,PCBP3
M209_0.6.txt	RBM45
M210_0.6.txt	HNRPDL
M211_0.6.txt	PCBP4,PCBP3
M227_0.6.txt	PTBP1,PTBP2,ROD1
M228_0.6.txt	PTBP1,PTBP2,ROD1
M229_0.6.txt	CELF3
M231_0.6.txt	EIF2S1
M232_0.6.txt	ELAVL1,ELAVL3
M234_0.6.txt	RBM47
M235_0.6.txt	RBM47
M236_0.6.txt	HNRNPR,SYNCRIP
M238_0.6.txt	RBM38

M240_0.6.txt	RBM38
M242_0.6.txt	HNRNPR,SYNCRIP
M243_0.6.txt	HNRNPR,SYNCRIP
M245_0.6.txt	NCL
M246_0.6.txt	RBMY1F,RBMXL2,RBMXL3,RBMXL1,RBMY1J,RBMY1A1,RBMY1E,RBMY1B,RBMY1D
M247_0.6.txt	RBMY1F,RBMXL2,RBMXL3,RBMXL1,RBMY1J,RBMY1A1,RBMY1E,RBMY1B,RBMY1D
M250_0.6.txt	CSDA
M254_0.6.txt	CSDA
M256_0.6.txt	ACO1
M260_0.6.txt	CSDA
M261_0.6.txt	SF3B4
M262_0.6.txt	QKI
M269_0.6.txt	ZFP36,ZFP36L2,ZFP36L1
M271_0.6.txt	HNRNPA1,HNRNPA3,ENSG00000215492,ENSG00000231942
M272_0.6.txt	SRSF1
M273_0.6.txt	SRSF1
M274_0.6.txt	SRSF2,ENSG00000180771
M275_0.6.txt	PABPC1,PABPC1L,ENSG00000250177
M290_0.6.txt	EIF4B
M291_0.6.txt	EIF4B
M292_0.6.txt	EIF4B
M296_0.6.txt	IGF2BP1
M297_0.6.txt	RBFOX2,RBFOX3
M298_0.6.txt	A2BP1,RBFOX2,RBFOX3
M307_0.6.txt	NONO
M316_0.6.txt	FUS,TAF15
M317_0.6.txt	SNRPA,SNRPB2
M318_0.6.txt	SNRPA,SNRPB2
M319_0.6.txt	SNRPA,SNRPB2
M320_0.6.txt	MBNL3,MBNL2,MBNL1
M323_0.6.txt	NOVA2,ENSG00000248163,ENSG00000249644
M325_0.6.txt	NOVA2,ENSG00000248163,ENSG00000249644
M328_0.6.txt	ELAVL2,ELAVL3
M329_0.6.txt	ELAVL2,ELAVL3
M330_0.6.txt	ELAVL2,ELAVL3
M331_0.6.txt	SRSF7
M332_0.6.txt	SRSF2,ENSG00000180771
M333_0.6.txt	SRSF9
M334_0.6.txt	SRSF4,SRSF6
M344_0.6.txt	RBMX,RBMY1F,RBMXL2,RBMXL3,RBMXL1,RBMY1J,RBMY1E,RBMY1B,RBMY1D
M345_0.6.txt	SNRPA,SNRPB2
M346_0.6.txt	SNRPA,SNRPB2

M347_0.6.txt	SNRPA,SNRPB2
M348_0.6.txt	SNRPA,SNRPB2
M349_0.6.txt	PABPC1,PABPC1L,ENSG00000250177
M350_0.6.txt	ZFP36,ZFP36L2,ZFP36L1
M351_0.6.txt	SRSF1
M352_0.6.txt	SRSF2,ENSG00000180771
M353_0.6.txt	SRSF7
M354_0.6.txt	YTHDC1

Supplemental Table 3. Table of motifs associated with each RNA binding protein.

Supplementary Methods

General RNA-seq processing

The analysis was performed as described in the Methods section of the main document, and shown graphically in Fig2A and FigS2. ENCODE paired end reads of 200 nt transcribed from the nonpoly A fraction of nuclear or total cellular RNA were aligned using TopHat. Using combination of custom Perl scripts, samtools, and bedtools, found pairs containing at least one spliced exon and intersected with bed of all introns (ucsc hg19 database) to select for alignment with one unspliced intron. Introns that contained additional splicing events within were discarded to prevent counting alt splicing events as splicing intermediates. All analyses were done only for intron pairs that had at least 10 intermediate reads

Additional Notes on the Simulation

The analysis was performed as described in the Methods section of the main document. Paired end, RNA-seq reads from the ENCODE dataset capable of identifying a spliced intermediate mapped to approximately 100,000 introns. For each intron pair identified in this manner with a total read number greater than 10, the number of informative reads at that intron pair were recorded for use in the simulation. The process of co-transcriptional splicing was simulated using published transcription and splicing rates and variances (Singh and Padgett, 2009). A random number generator weighted by the variance-estimated distribution assigned a single transcription rate for the synthesis of the intron/exon/intron/exon region necessary to observe an order of splicing. The average transcription rate was 3.87 kb/min. Each intron was assigned a splicing rate. The length-independent value of 0.169 intron/min was the finding of (Singh and Padgett, 2009). Each intron pair was transcribed to create an equal number of reads observed in the data. Splicing was allowed to occur following the synthesis of both 5'ss and 3'ss of the intron. The simulation was performed in MATLAB. Simulated paired end reads were generated. Data analysis on simulated data was performed through the analysis pipeline developed for the observed data. Simulations and analysis were repeated ensuring no alternatively spliced transcripts were included. Constitutive introns were defined as unique pairs that did not share 5'ss and 3'ss with any other annotated intron in UCSC known gene annotation. Restricting the analysis to constitutively spliced introns had no effect on the observed data or simulation. Figure S1 includes simulation on the constitutive set and includes an example of varying parameters (i.e. in this case, variance is ten fold higher than observed). Increasing the variance of splicing rates results in more always towards upstream introns splicing first. All scripts, intron lists, simulation counts and binned data utilized histogram data can be found at: **http://fairbrother.biomed.brown.edu/data/Order**

Supplementary Files Simulation (Figure 2)

get_pairs_for_simulation.pl
matlab_sim_10x_var_hist_points.txt
matlab_sim_10x_var_output.txt
matlab_sim_observed_var_hist_points.txt
matlab_simulation_observed_var_output.txt
new_pair_list_for_simulation.txt
unique_inrons_consecutive_pairs_030315.txt

Additional Notes on the Analysis of Exon Skipping

The analysis was performed as described in the Methods section of the main document. Using the list of introns from UCSC, all introns where both splice sites exactly matched the 3' splice site from one intron and the 5' splice site from the next consecutive intron. The transcript containing the extra exon was then used for "before", "after", and "internal" splicing events. For skipping of two exons, both splice sites were required to exactly match one intron and the second consecutive intron from a transcript. The transcript containing the two internal exons was used to determine splicing percentages for each event.

Supplementary Files Exon Skipping (Figure 5)

double_skipped_exon_internal_trios.txt
double_skipped_exons_after.txt
double_skipped_exons_before.txt
get_intron_before_skip.txt
get_pairs_from_consecutive_introns.pl
get_skipped_exons.pl
get_trios_for_single_skips.pl
intersect_trios_single_intron.pl
single_skipped_exon_internal.txt
single_skipped_exons_after.txt
single_skipped_exons_before.txt
skipped_exons_from_pair_file.pl

Additional notes on Analysis of First, Internal and Last exons.

The analysis was performed as described in the Methods section of the main document. Annotated (UCSC known genes) intron number in transcripts was utilized to identify first and last introns. For any pair that contained the first and second introns in a gene, this was counted as a "first" pair. Pairs containing the second-to-last and last introns in a gene were called "last" pairs. "Middle" pairs were

those that did not fit into either category above. Transcripts containing fewer than 3 introns were excluded from this analysis.

Supplementary Files First, Internal, Last Introns (Figure 5)

first_introns_s…air_counts.txt
last_intron_scores.txt
middle_intron_scores.txt

Additional Notes on Analysis of Alt SS Usage.

The analysis was performed as described in the Methods section of the main document. Using all transcripts from UCSC, alternate 5' splice sites were determined when multiple introns had an identical 3' splice site and different 5' splice sites. To ensure usage of the distal site was not counted as a partial splicing event, the 5' splice site that gave the shortest intron was chosen to be used in the pair analysis. Alternate 3' splice sites were determined from introns sharing a 5' splice site with different 3' splice sites, and again the shortest intron was used.

Supplementary Files Alternate 3'ss and 5'ss (Figure 5)

after_alt_3prime_scores_only.txt
after_alt_3prime.txt
after_alt_5prime_scores_only.txt
after_alt_5prime.txt
alt_3prime_introns_shortest_sorted_by_3.bed
alt_3prime_introns_shortest.bed
alt_5prime_introns_shortest_sorted_by_3.bed
alt_5prime_introns_shortest.bed
alt_splicing_introns_forward_3prime_shortest.bed
alt_splicing_introns_forward_5prime_shortest.bed
alt_splicing_introns_reverse_3prime_shortest.bed
alt_splicing_introns_reverse_5prime_shortest.bed
before_alt_3prime_scores_only.txt
before_alt_3prime.txt
before_alt_5prime_scores_only.txt
before_alt_5prime.txt
collapse_ss_counts_alt.pl
forward_intron_pairs_by_3.txt
forward_intron_pairs_by_5.txt
forward_intron_pairs.txt
get_alt_numbers_and_genes.pl
get_alt_splice_sites.pl
get_introns_after_alt.pl
get_introns_before_alt.pl
get_introns_matching_ss.pl
get_introns_sharing_ss.pl
get_pairs_from_introns.pl
reverse_intron_pairs_by_3.txt
reverse_intron_pairs_by_5.txt
reverse_intron_pairs.txt
revised_pairs
temp_pair_counts_by_first_3prime.txt
temp_pair_counts_by_second_3prime.txt
temp_pair_counts_by_second_5prime.txt

Additonal Notes on the Length

Length of annotated introns was determined, and the difference in length between the introns in each pair was determined by subtracting the downstream intron's length from the upstream intron's length, so that a negative number represented a longer downstream intron and a positive number represented a longer upstream intron. Intron pairs were then binned according to their length difference. For each bin, the fraction of pairs where the downstream intron spliced first at least 95% of

the time was determined and then plotted. Splicing percentage histograms were also created for each bin.

Supplementary Files for Exon Length (Figure 6)

- all_splicing_pair_counts_…g_length_differences.txt
- bins_by_diff
- intron_differences_and_scores.txt
- intron_lengths_scores_by_second_length.txt
- intron_lengths_scores.txt
- pair_counts_min_10_intron_lenghths.txt

Additional notes on Intron Circles

The analysis was performed as described in the Methods section of the main document. A list of known circles generated from Illumina Body Map 2.0 seq data was used to find circles that contained at least 2 exons in the data set. To determine splicing order for before the circles, the beginning coordinate of the circle was matched to the internal exon of an upstream/downstream pair for that exon, matching it to the last (downstream) coordinate of the upstream intron. For after the circles, the end coordinate of the circle was matched to the first (upstream) coordinate of a downstream intron in a pair. Histograms were then generated for both the "before" pairs and the "after" pairs.

Supplementary Files for Circles (Figure 6)

- all_circles.txt
- get_splicing_from_after_circles.pl
- get_splicing_from_circles.pl
- splicing_after_circles_data.txt
- splicing_after_circles_minus.txt
- splicing_after_circles_plus.txt
- splicing_after_circles_unique.txt
- splicing_after_circles.txt
- splicing_before_circles_a.txt
- splicing_before_circles_percent_downstream.txt
- splicing_before_circles_plus_unique.txt
- splicing_before_circles_plus.txt
- splicing_before_circles_unique.txt
- splicing_before_circles.txt
- unique_circles_minus_unique_start_for_after.txt
- unique_circles_minus_unique_start.txt
- unique_circles_minus.txt
- unique_circles_plus_unique_start_for_after.txt
- unique_circles_plus_unique_start.txt
- unique_circles_plus.txt
- unique_circles.txt
